# Supplementary material for: Design, Synthesis, Electronic Properties, and X-ray Structural Characterization of Various Modified Electron-Rich Calixarene Derivatives and Their Conversion to Stable Cation Radical Salts
Source: Molecules. 2022 Sep 14;27(18):5994. doi: 10.3390/molecules27185994 (PMC9501969; doi:10.3390/molecules27185994)
Supplement: Supplementary file 1 [file molecules-27-05994-s001.zip › molecules-1891595-supplementary.pdf]

SUPPORTING INFORMATION

Design, Synthesis, Electronic Properties and X-ray Structural Characterization of Various Modified Electron-Rich Calixarene Derivatives and Their Conversion to Stable Cation Radical Salts.

Table of Contents

Preparation and UV of Mono-Cation Radicals Using $MA^{+\bullet}$ and/or $MB^{+\bullet}$. **Figures S1 and S2**	page S2
Preparation and UV of Mono-Cation Radicals for the Model (M) Using $Nap^{+\bullet}$. **Figure S3**	page S3
$^{1}H/^{13}C$ NMR spectra and analytical data **Figures S4-S15**	page S6-S17
X-ray structural data for A **Figure S16**	page S18
X-ray structural data for B **Figure S17**	page S19
X-ray structural data for C **Figure S18**	page S20
X-ray structural data for D **Figure S19**	page S21
X-ray structural data for $[E]^{+\cdot}$ $SbCl_6$ **Figure S20**	page S22
X-ray[3] structural data for E **Figure S21**	page S23
X-ray[3] structural data for $[E,NO]^{+}$ $]SbCl_6^{-}$ **Figure S22**	page S23
References	page S156

Preparation of Mono-Cation Radicals Using $MA^{+\bullet}$ and/or $MB^{+\bullet}$.

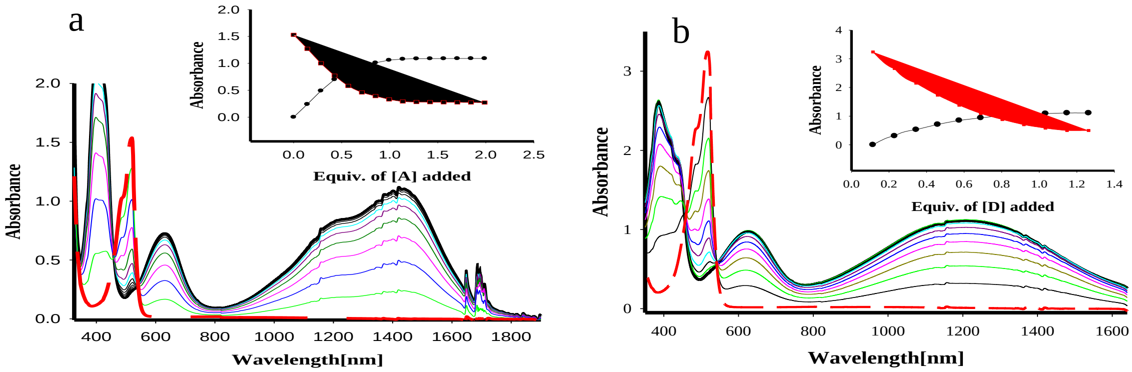

Figure S1. (a) Left is Spectral changes upon the reduction of 2.1 x 10^{-4} M **$MA^{+\bullet}$** (red dashed line) by incremental addition of 8.8 x 10^{-3} M **A** to **$A^{+\bullet}$** (black solid line) in dichloromethane at 22 °C. Inset: A plot of depletion of absorbance of **$MA^{+\bullet}$** (squares, monitored at 516 nm) and an increase of the absorbance of **$A^{+\bullet}$** (circles, monitored at 1420 nm) against the equivalent of added **A**. (b) Right is Spectral changes upon the reduction of 1.9 x 10^{-4} M **$MA^{+\bullet}$** (red dashed line) by incremental addition of 4.1 x 10-3 M **D** to **$D^{+\bullet}$** (black solid line) in dichloromethane at 22 °C. Inset: A plot of depletion of absorbance of **$MA^{+\bullet}$** (squares, monitored at 516 nm) and an increase of the absorbance of **$D^{+\bullet}$** (circles, monitored at 1200 nm) against the equivalent of added **D**.

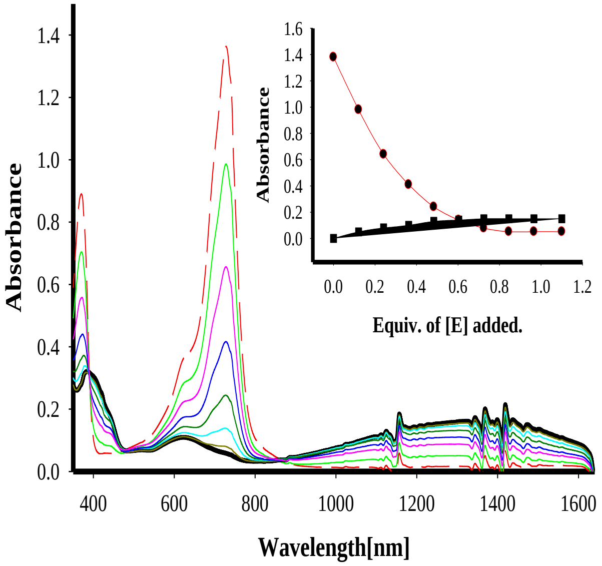

Figure S2. Spectral changes upon the reduction of 4.5 x 10^{-5} M **$MB^{+\bullet}$** (red dashed line) by incremental addition of 3.38 x 10^{-3} M **E** to **E**+• (black solid line) in dichloromethane at 22 °C. Inset: A plot of depletion of absorbance of **$MB^{+\bullet}$** (circles, monitored at 728 nm) and an increase of the absorbance of **$E^{+\bullet}$** (squares, monitored at 1300 nm) against the equivalent of added **E**.

Preparation of Mono-Cation Radicals for the Model (M) Using Nap$^{+\bullet}$.

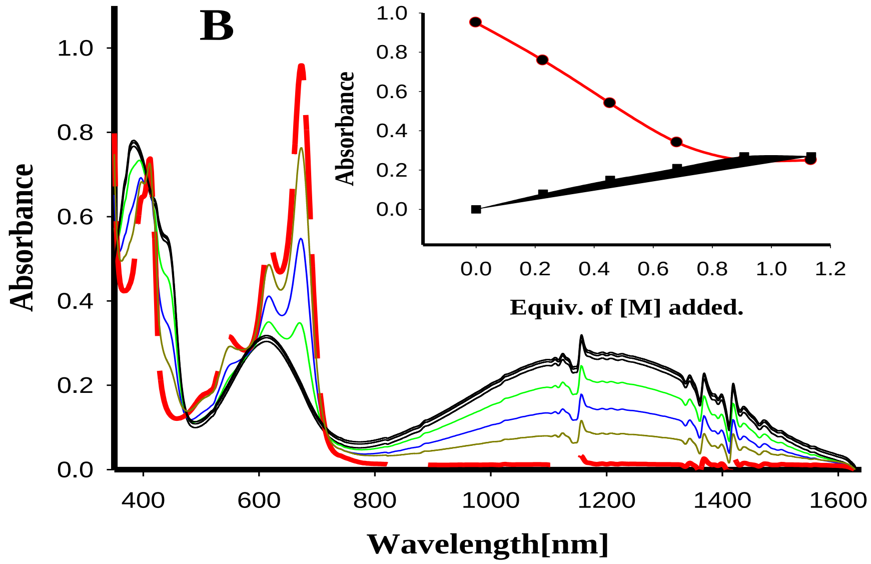

Figure S3. Right is Spectral changes upon the reduction of 1.1 x 10^{-4} M **Nap**$^{+\cdot}$ (red dashed line) by an incremental addition of 9.1 x 10^{-3} M **M** to its **M**$^{+\cdot}$ (black solid line) in dichloromethane at 22 °C. Inset: A plot of depletion of absorbance of **Nap**+• (squares, monitored at 672 nm) and an increase of the absorbance of **M**$^{+\bullet}$ (circles, monitored at 1190 nm) against the equivalent of added **M**.

General procedure for the preparation of NO complexes of various calixarene derivatives. Isolation of [5,11,17,23-*tetrakis*(2,5-dimethoxy-4-methylphenyl)-25,26,27,28-tetrapropoxycalix[4]arene/NO]$^+$ (1,3-alternate conformer) (equation S1).

Eq. S1

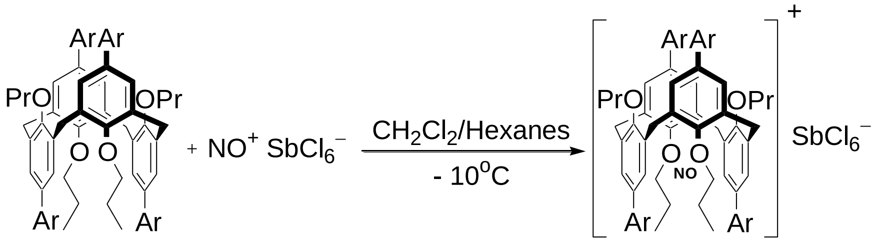

To a pale yellow solution of NO$^+$ $SbCl_6^-$ (20 mg, 0.054 mmol) in dichloromethane (15 mL) at 22 °C was added (colorless) crystalline **E** (70 mg, 0.06 mmol). The solution immediately took on a dark blue coloration. The dark-colored mixture was stirred at –10 °C for 5 min and an aliquot was removed and diluted with anhydrous dichloromethane. The quantitative UV-vis spectral analysis of the blue solution reveled the appearance of an intense absorption band with λ_{max} = 690 nm and a molar extinction coefficient ε_{690} = 3000 M^{-1} cm^{-1}. The remaining blue solution was carefully layered with dry hexanes (10 mL) and the sample was carefully placed in the refrigerator and left undisturbed for several days.

Binding of Nitric Oxide (NO) to Various Modified Calixarene Donors. The solution of mono-cation radical salts of the calixarene ethers B, C, and E were prepared using triethyloxonium hexachloroantimonate or MB$^{+\cdot}$ as 1-electron oxidant, or by an equivalent electrochemical method. The ready availability of these calixarene cation-radical salts, with electron-deficient cavities

owing to the electronic coupling between charge-bearing aryl moieties and calixarene cores, now allows us to directly examine the spectral and structural changes attendant upon association of nitric oxide as follows.

UV-Vis Spectroscopy. When a dichloromethane solution of the isolated monoarylcalixarene ether cation radical [$B^{+\bullet}$ $SbCl_6^-$] (λ_{max} = 1460, 630, and 386 nm, ε_{1460} = 5600 M^{-1} cm^{-1}) was exposed to gaseous nitric oxide (NO) at 22 °C, the green color was immediately replaced by a dark blue coloration, and the UV-vis spectral analysis of the resulting solution showed a characteristic absorption spectrum with a broad absorption band at λ_{max} = 590 nm (ε_{590} = 8860 M^{-1} cm^{-1}). Note that the observed (UV-vis) spectrum of $[B/NO]^+$ association is characteristically similar to that observed for $[X/NO]^+$ complex of *t*-butylcalixarene tetrapropyl ether (λ_{max} = 569 nm, ε_{569} = 5600 M^{-1} cm^{-1})[1].

In another experiment[2], a solution of B was added to a solution of NO^+ $SbCl_6^-$ in dichloromethane in a Schlenk flask under an argon atmosphere. The solution immediately takes on a dark-blue coloration and the solution was stable at room temperature for several days. The (UV-vis) spectral analysis of the blue-colored solution shows an absorption spectrum (λ_{max} = 590 nm) identical to that obtained above by exposure of $B^{+\bullet}$ to gaseous NO. Moreover, a quantitative infrared spectral analysis of the blue solution confirmed that the N–O stretching band at 1910 cm^{-1} in $[B/NO]^+$ is characteristically close to that observed for free nitric oxide (1876 cm^{-1}).[3] [Note that the spectral characteristics of cation radical of $B^{+\bullet}$ and the $[B/NO]^+$ association, prepared from different nitrosonium salts (such as NO^+ BF_4^- or PF_6^-) are identical to those obtained with NO^+ $SbCl_6^-$.

Thus, a dual mode of preparation of $[B/NO]^+$ from $B^{+\bullet}$ and NO or B and NO^+, which is accompanied by dramatic color changes, can be formulated as follows (Equation S2).

Eq. S2

B$^{+\bullet}$ 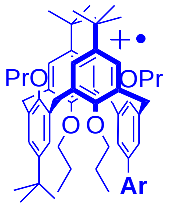

+ NO $\xrightarrow{22\ ^{o}C}$ [**B**/NO]$^+$ $\xleftarrow{22\ ^{o}C}$ **B** + NO^+

In a similar vein, the cation-radical salts of calixarene ether C and E capture gaseous nitric oxide quantitatively at room temperatures. It is noteworthy that although the (UV-vis) absorption spectra of the cation radical of parent calixarene $X^{+\bullet}$ (λ_{max} = 440 nm and a weak broad band extending beyond 1100 nm) is dramatically different from those of the arylated calixarenes B, C, and E, the observed spectra of the corresponding NO-bonded complexes displayed a characteristically similar broad absorption band for all $[Calix/NO]^+$ complexes. Such an observation suggests a similar penetration (or binding) of NO within the cylindrical calixarene cavities which are more or less similar in various calixarene derivatives of 1,3-alternate conformer.

The high affinity of $Calix^{+\cdot}$ toward NO (K_{NO} > 10^8 M^{-1}) [Binding constant was too large to be determined directly and thus was estimated using Venus fly trap (K_{ass} = 3 x 10^6 M^{-1} for NO binding) according to a competition method[1] allowed the ready isolation of single crystals[2] of blue $[E/NO]^+$ from a mixture of dichloromethane and hexanes at 0 °C.

Experimental Section

Materials.

t-Butylcalix[4]arene, *p*-toluenesulfonyl chloride, bromine, treiethyloxonium hexachloroantimonate, *bis*(triphenylphosphine)palladium dichloride, aluminum chloride, cesium carbonate, barium oxide, barium hydroxide, iodomethane, iodopropane, methylethyl ketone (Aldrich), *N,N*-dimethylformamide (Fisher), phenol (Mallinckrodt), *N*-bromosuccinimide (Avocado), sulfuric acid, chloroform, methanol, hexanes, ethyl acetate and acetic acid were commercially available, and were used as received unless otherwise specified. Anhydrous tetrahydrofuran (THF) was prepared by refluxing the commercial tetrahydrofuran (Aldrich) over lithium tetrahydroaluminate under an argon atmosphere for 24 hours followed by distillation. It was stored under an argon atmosphere in a Schlenk flask equipped with a Teflon valve fitted with Viton O-rings. Dichloromethane (Aldrich) was repeatedly stirred with fresh aliquots of conc. sulfuric acid (~10 % by volume) until the acid layer remained colorless. After separation it was washed successively with water, aqueous sodium bicarbonate, water, and saturated aqueous sodium chloride and dried over anhydrous calcium chloride. The dichloromethane was distilled twice from P_2O_5 under an argon atmosphere and stored in a Schlenk flask equipped with a Teflon valve fitted with Viton O-rings. The hexanes and toluene were distilled from P_2O_5 under an argon atmosphere and then refluxed over calcium hydride (~12 hrs). After distillation from CaH_2, the solvents were stored in Schlenk flasks under argon atmosphere.

Preparation of *n*-propyl tosylate. *n*-Propyl tosylate was synthesized according to a literature procedure.[4]A solution of 1-propanol (6.2 mL, 83 mmol) in dry pyridine (60 mL) was cooled to –5 ^{o}C in an ice-salt bath. Purified *p*-toluenesulfonyl chloride (17.45 g, 91.5 mmol) was added all at once and the suspension was stirred with cooling in an ice-salt bath until all *p*-toluenesulfonyl chloride has dissolved. After keeping the reaction mixture at 0 ^{o}C for another 2 h, water (110 mL) was slowly added with swirling and cooling so that the temperature did not rise above +5 ^{o}C. The aqueous reaction mixture was extracted with chloroform (3 x 60 mL) and the combined chloroform extracts were washed successively with ice-cold dilute sulfuric acid (5 N, 2 x 50 mL), water (2 x 50 mL) and saturated sodium bicarbonate solution (3 x 50 mL). The chloroform layer was dried over anhydrous magnesium sulfate, filtered, and evaporated under reduced pressure to give almost pure *n*-propyl tosylate which was stored in the fridge. Yield: 10.6 g (54%); ^{1}H NMR ($CDCl_3$), δ: 0.89 (t, 3H), 1.66 (sextet, 2H), 2.45 (s, 3H), 3.98 (t, 2H), 7.36 (d, 4H), 7.77 (d, 4H). GC-Ms: *m/z* 214 (M^+) calcd for $C_{10}H_{14}O_3S$.

4-Bromo-2,5-dimethoxytoluene. 4-Bromo-2,5-dimethoxytoluene was synthesized according to a literature procedure[5], and was purified by recrystallization from ethanol. Yield: 96%; mp 92- 94 ^{o}C (lit[5] mp 92-93 ^{o}C; ^{1}H NMR (CDCl3), δ: 2.21 (s, 3H), 3.8 (s, 3H), 3.85 (s, 3H), 6.75 (s, 1H), 6.75 (s, 1H); GC-Ms: *m/z* 231 (M^+) calcd for $C_9H_{11}BrO_2$.

Calixarene Derevatives – NMR

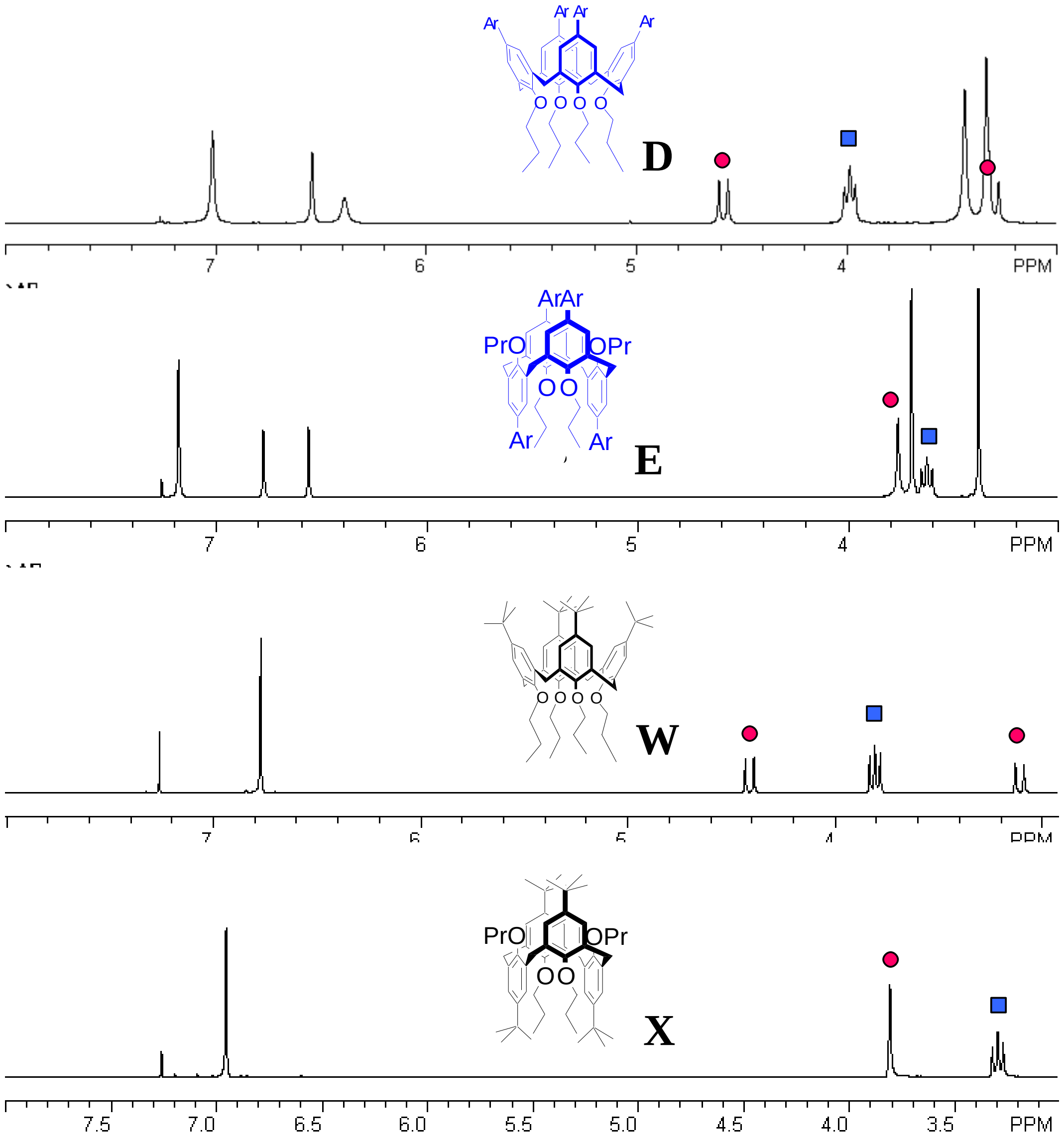

Figure S4: The partial 1H NMR spectra of Calixarene ether **D**, **E**, **W** and **X** in $CDCl_3$ at room temperature, ● -methylenes; ■ OCH_2 of propoxy group.

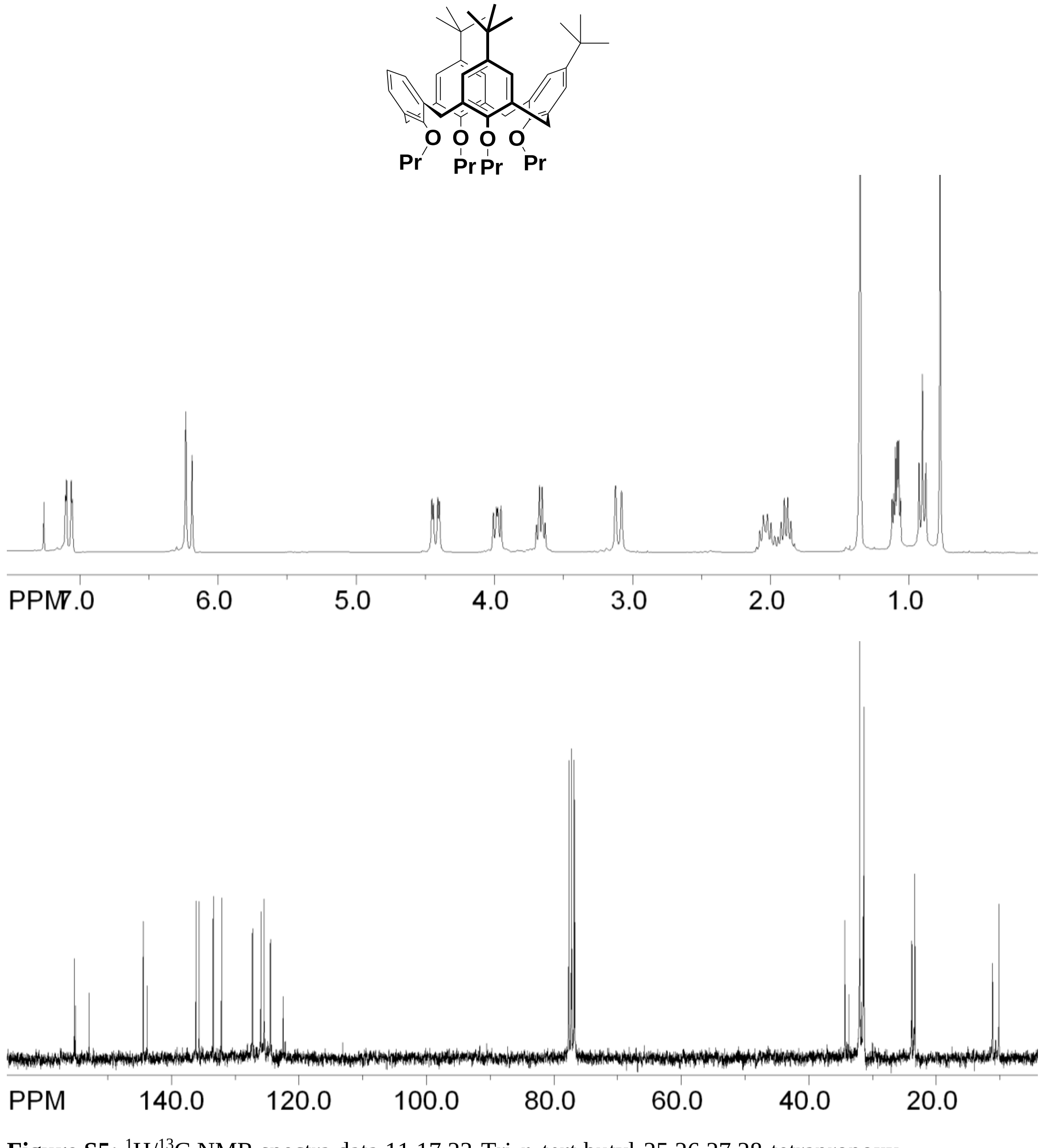

Figure S5: ^{1}H/^{13}C NMR spectra data 11,17,23-Tri-*p*-*tert*-butyl-25,26,27,28-tetrapropoxy calix[4]arene (5) in $CDCl_3$.

^{1}H NMR ($CDCl_3$) δ: 0.77 (s, 9H), 0.90 (t, *J* = 7.5 Hz, 6H), 1.08 (m, 6H), 1.35 (s, 18H), 1.89 (sextet, *J* = 7.08 Hz, 4H), 2.04 (sextet, *J* = 7.5 Hz, 4H), 3.08 (s, 2H), 3.12 (s, 2H), 3.65 (t, *J* = 6.8 Hz, 2H), 3.67 (t, *J* = 6.8 Hz, 2H), 3.98 (m, 4H), 4.35 (d, *J* = 3.6 Hz, 2H), 4.45 (d, *J* = 3.6 Hz, 2H), 6.19 (s, 2H), 6.23 (s, 3H), 7.05 (d, *J* = 2.3 Hz, 2H), 7.09 (d, *J* = 2.3 Hz, 2H); ^{13}C NMR ($CDCl_3$) δ: 10.17, 11.14, 11.19, 23.37, 23.79, 32.88, 31.35, 31.48, 31.48, 32.03, 33.73, 34.34, 76.68, 77.20, 77.33, 12.46, 124.47, 125.46, 125.97, 127.28, 132.15, 133.45, 135.65, 136.15, 143.82, 144.42, 152.89, 155.07, 155.22

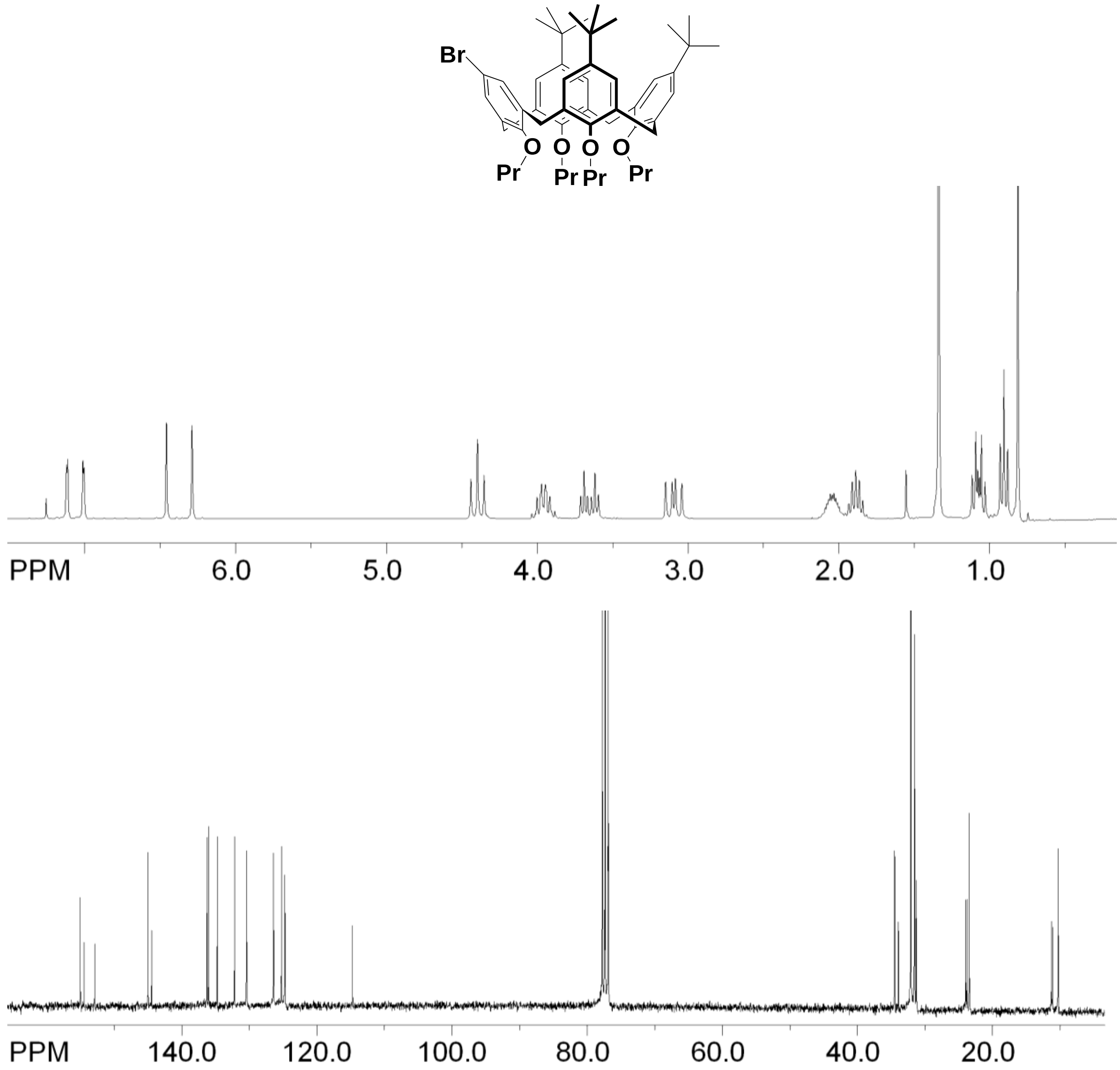

Figure S6: $^{1}H/^{13}C$ NMR spectra data of 5-Bromo-11,17,23-tri-*p*-*tert*-butyl-25,26,27,28-tetrahydroxy calyx[4]arene (6) in $CDCl_3$.

^{1}H NMR ($CDCl_3$) δ: 0.81 (s, 9H), 0.91 (t, *J* = 7.53 Hz, 6H), 1.07 (m, 6H), 1.33 (s, 18H), 1.89 (sextet, *J* = 6.7 Hz, 4H), 2.04 (m, 4H), 3.06 (m, 4H), 3.62 (t, *J* = 7.05 Hz, 2H), 3.69 (t, *J* = 6.9 Hz, 2H), 3.96 (m, 4H), 4.39 (t, *J* = 13.14 Hz, 4H), 6.29 (s, 2H), 6.45 (s, 2H), 7.00 (d, *J* = 2.25 Hz, 2H), 7.11 (d, *J* = 2.25 Hz, 2H); ^{13}C NMR ($CDCl_3$) δ: 10.27, 11.09, 11.26, 23.43, 23.76, 23.97, 31.27, 31.54, 32.08, 33.92, 34.47, 114.69, 124.71, 125.22, 126.39, 130.37, 132.16, 134.72, 135.97, 136.24, 144.44, 144.97, 152.80, 154.45, 154.98.

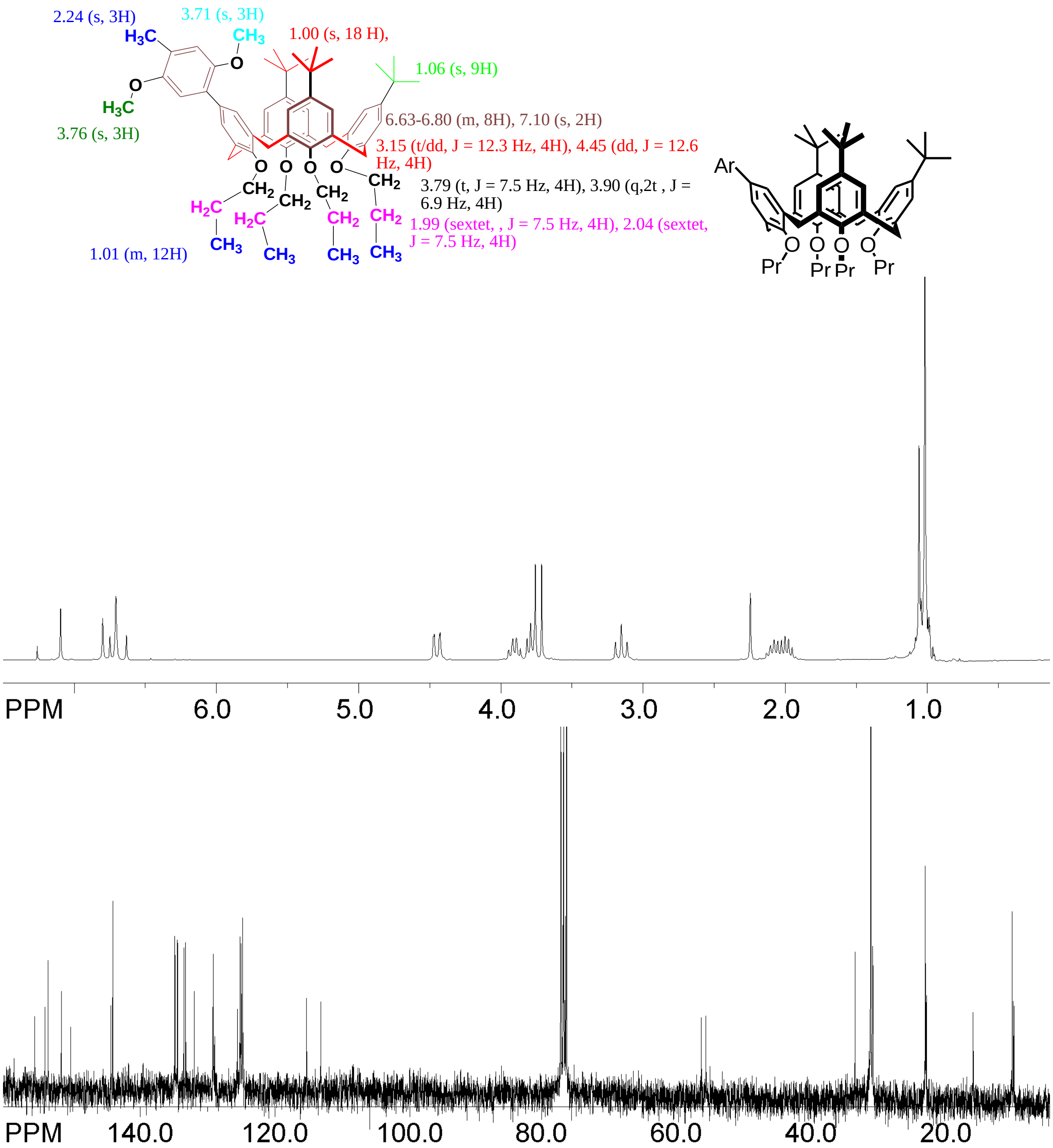

Figure S7: $^{1}H/^{13}C$ NMR spectra data of 5-(2,5-Dimethoxytoluene)-11,17,23-tri-*p*-*tert*-butyl-25,26,27,28-tetrahydroxy calix[4]arene (Cone Conformer) (A) in $CDCl_3$.

^{1}H NMR ($CDCl_3$) δ: 1.00 (s, 18 H), 1.01 (m, 12H), 1.06 (s, 9H), 1.99 (sextet, , *J* = 7.5 Hz, 4H), 2.04 (sextet, *J* = 7.5 Hz, 4H), 2.24 (s, 3H), 3.15 (t, *J* = 12.3 Hz, 4H), 3.71 (s, 3H), 3.76 (s, 3H), 3.79 (t, *J* = 7.5 Hz, 4H), 3.90 (q, *J* = 6.9 Hz, 4H), 4.45 (dd, *J* = 12.6 Hz, 4H), 6.63-6.80 (m, 8H), 7.10 (s, 2H); ^{13}C NMR ($CDCl_3$) δ 10.49, 10.59, 10.77, 16.55, 23.47, 23.59, 23.66, 31.39, 31.69, 31.76, 34.04, 56.15, 56.57, 56.83, 77.01,77.20, 77.32. 113.23, 115.33, 124.80, 125.01, 125.19, 125.57, 128.97, 129.19, 131.97, 133.26, 133.52, 134.67, 134.85, 144.07, 144.35, 150.28, 151.69, 153.66, 154.09, 155.62.

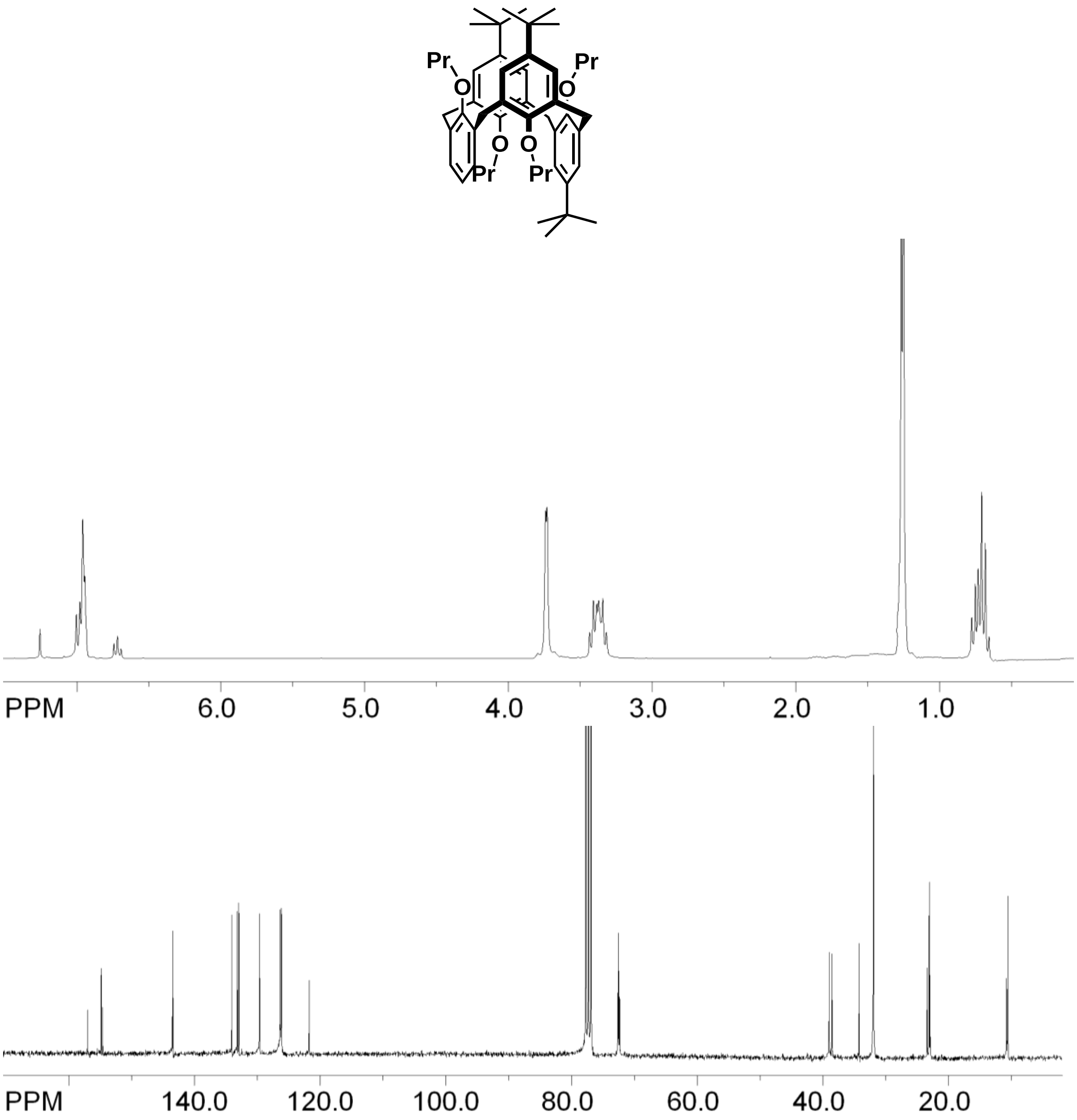

Figure S8: $^{1}H/^{13}C$ NMR spectra data of 11,17,23-Tri-*p*-*tert*-butyl-25,26,27,28-tetrapropoxycalix[4]arene(1,3-Alternate Conformer) (7)) in $CDCl_3$.

^{1}H NMR ($CDCl_3$), δ: 0.70 (m, 12H), 1.11-1.33 (m, 8H), 1.25 (s, 18H), 1.27 (s, 9H), 3.38 (m, 4H), 3.73 (s, 4H), 3.74 (s, 4H), 6.72 (t, *J* = 7.5 Hz, 1H), 6.94-7.00 (m, 8H); ^{13}C NMR ($CDCl_3$) δ: 10.48, 10.52, 10.73, 22.92, 23.01, 23.33, 31.85, 31.89, 34.15, 34.16, 38.51, 38.92, 72.40, 72.45, 72.51, 125.15, 126.03, 126.11, 126.28, 129.56, 132.83, 132.88, 133.11, 133.98, 143.34, 143.41, 154.52, 154.75, 154.95.

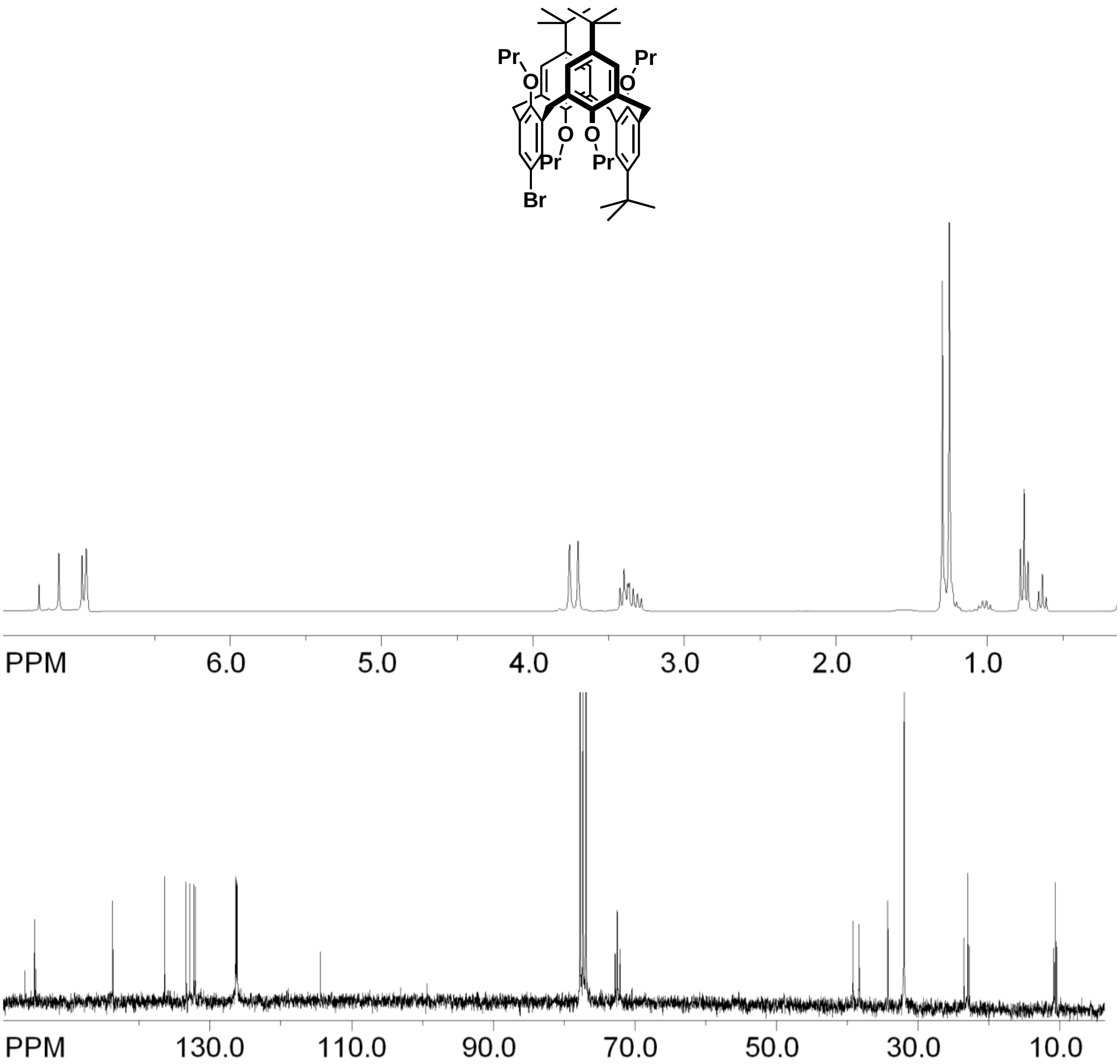

Figure S9: $^{1}H/^{13}C$ NMR spectra data of 5-Bromo-11,17,23-tri-*p*-*tert*-butyl-25,26,27,28-tetrahydroxy calix[4]arene (1,3-alternate Conformer) (8) in $CDCl_3$.

^{1}H NMR ($CDCl_3$) δ: 0.64 (t, *J* = 7.59 Hz, 3H), 0.76 (t, *J* = 7.41 Hz, 9H), 1.02 (sextet, *J* = 7.83 Hz, 2H), 1.25 (s, 18H), 1.28 (m, 6H), 1.36 (s, 9H), 3.36 (m, 8H), 3.70 (s, 4H), 3.76 (s, 4H), 6.97 (s, 4H), 6.98 (s, 2H), 7.13 (s, 2H); ^{13}C NMR ($CDCl_3$) δ: 10.36, 10.57, 10.75, 22.58, 22.88, 23.12, 31.85, 31.89, 34.15, 34.19, 38.23, 39.15, 72.02, 72.37, 72.57, 114.26, 126.06, 126.18, 126.26, 131.96, 132.19, 132.74, 133.26, 136.31, 143.64, 154.32, 154.65, 155.42

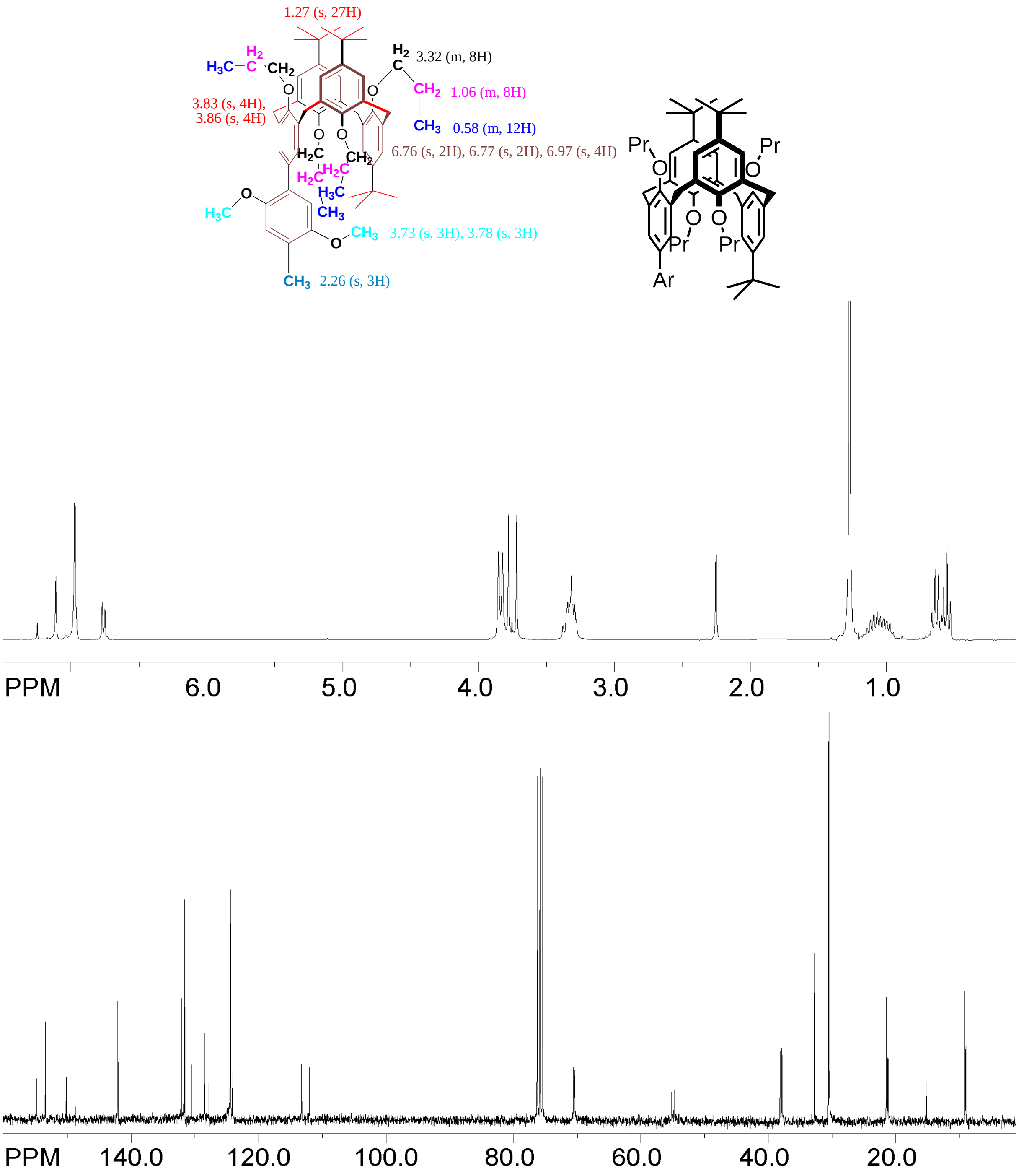

Figure S10: $^{1}H/^{13}C$ NMR spectra data of 5-(2,5-Dimethoxytoluene)-11,17,23-tri-*p*-*tert*-butyl-25,26,27,28-tetrahydroxy calix[4]arene (1,3-Alternate Conformer) (B) in $CDCl_3$.
δ: 0.58 (m, 12H), 1.06 (m, 8H), 1.27 (s, 27H), 2.26 (s, 3H), 3.32 (m, 8H), 3.73 (s, 3H), 3.78 (s, 3H), 3.83 (s, 4H), 3.86 (s, 4H), 6.76 (s, 2H), 6.77 (s, 2H), 6.97 (s, 4H), 7.11 (s, 2H); ^{13}C NMR ($CDCl_3$) δ 10.37, 10.43, 10.56, 16.58, 22.56, 22.66, 22.85, 31.88, 34.16, 39.25, 39.52, 56.19, 56.55, 71.75, 71.82, 71.91, 113.40, 114.65, 125.49, 125.79, 129.20, 129.85, 131.96, 132.97, 133.08, 133.53, 143.50, 150.22, 151.59, 154.87, 156.25.

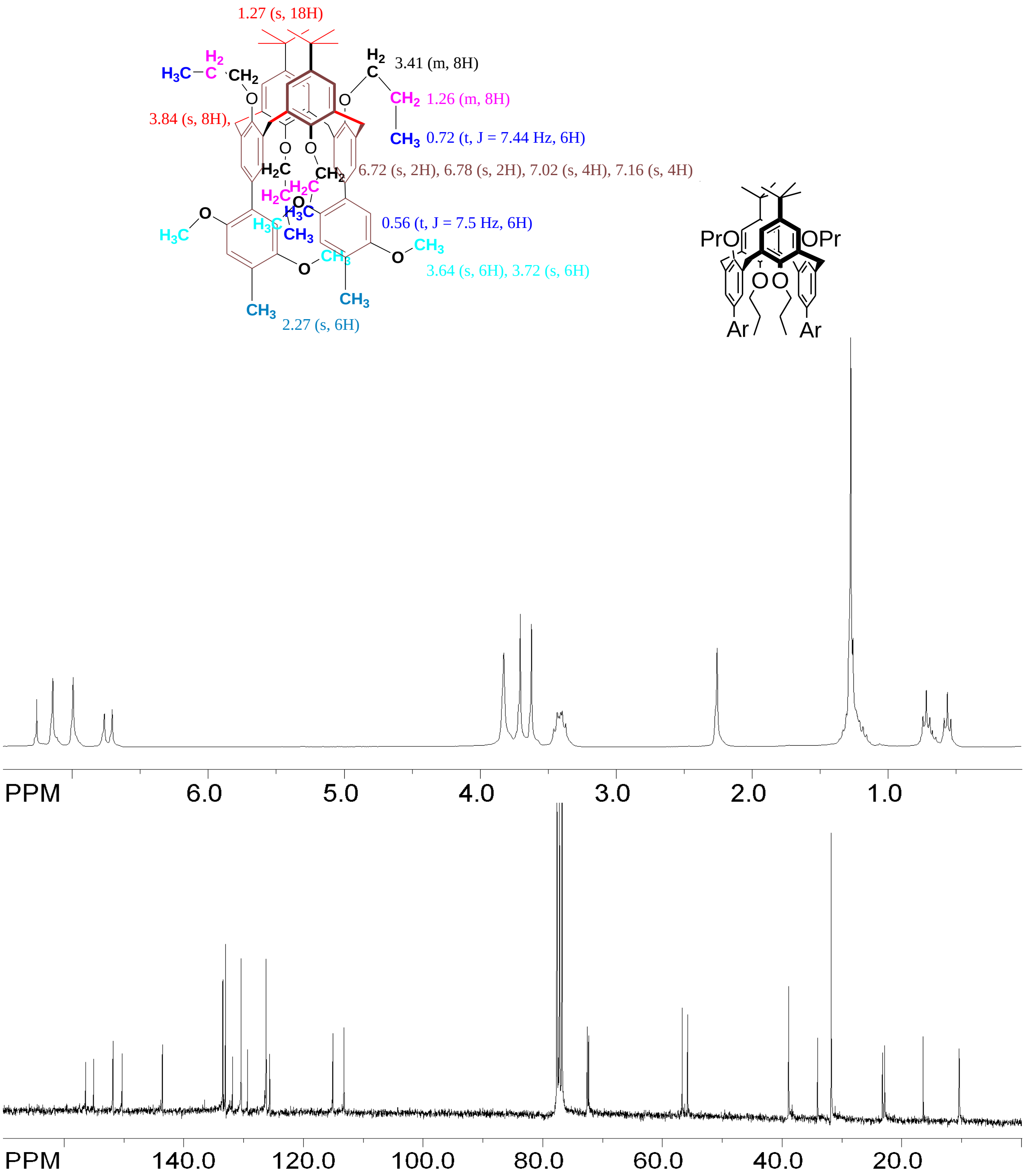

Figure S11: $^{1}H/^{13}C$ NMR spectra data of 11,23-*Bis*(2,5-dimethoxy-4-methylphenyl)-5,17-di-*tert*-butyl-25,26,27,28-tetrapropoxycalix[4]arene (1,3-Alternate Conformer) (C) in $CDCl_3$.

^{1}H NMR ($CDCl_3$) δ: 0.56 (t, *J* = 7.5 Hz, 6H), 0.72 (t, *J* = 7.44 Hz, 6H), 1.26 (m, 8H), 1.27 (s, 18H), 2.27 (s, 12H), 3.41 (m, 8H), 3.64 (s, 6H), 3.72 (s, 6H), 3.84 (s, 8H), 6.72 (s, 2H), 6.78 (s, 2H), 7.02 (s, 4H), 7.16 (s, 4H); ^{13}C NMR ($CDCl_3$) δ: 10.18, 10.23, 22.68, 23.01, 31.6, 33.85, 38.71, 55.58, 56.52, 72.13, 72.36, 112.99, 114.87, 125.41, 126.02, 129.13, 130.19, 131.63, 132.79, 133.24, 143.34, 150.11, 151.62, 154.83, 156.18.

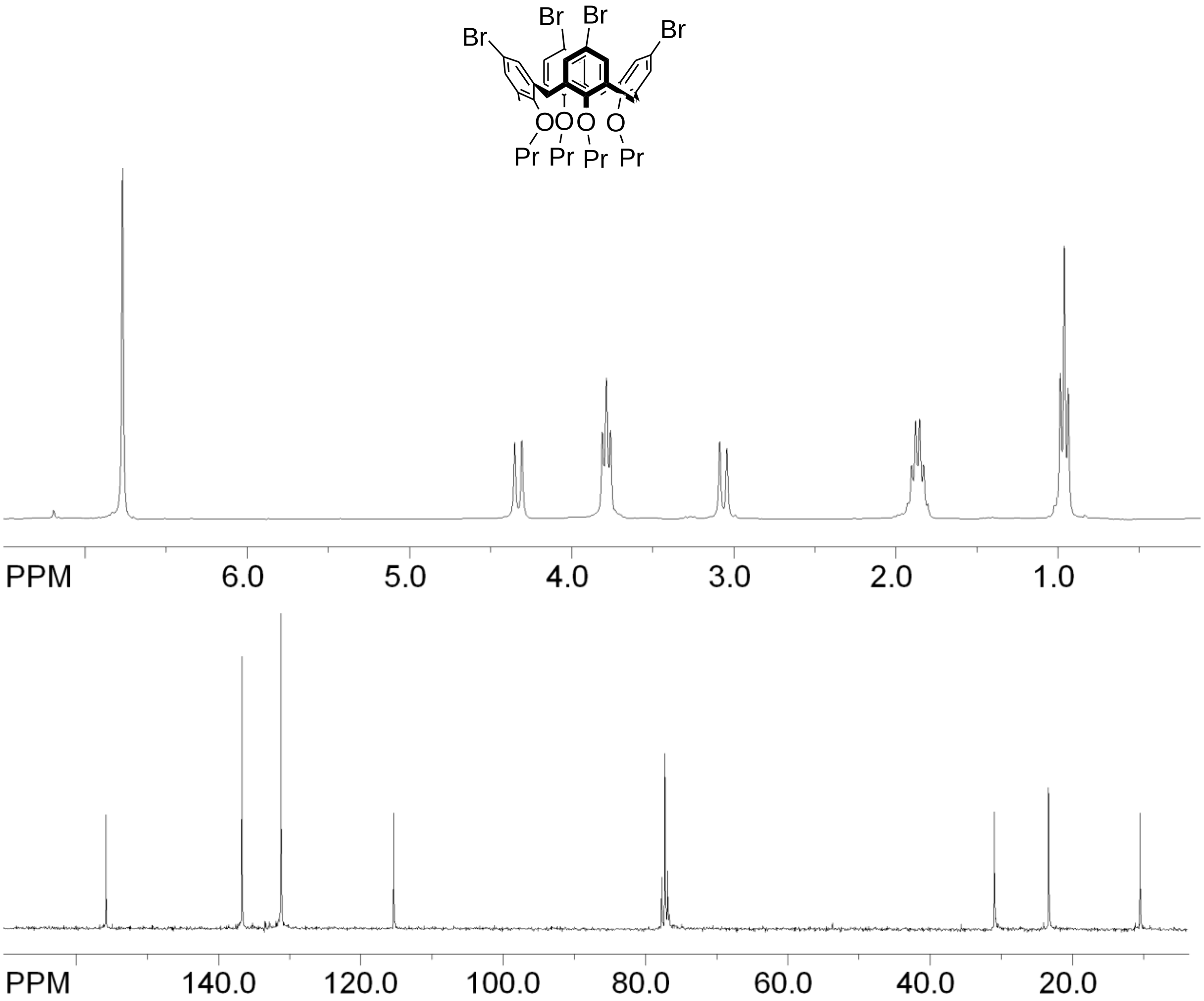

Figure S12: $^{1}H/^{13}C$ NMR spectra data of 5,11,17,23-Tetrabromo-25,26,27,28-tetrapropoxycalix[4]arene (Cone Conformer) (15) in $CDCl_3$.

^{1}H NMR ($CDCl_3$) δ: 0.95 (t, *J* = 7.41 Hz, 12H), 1.91 (sextet, *J* = 7.41 Hz, 8H), 3.10 (d, *J* = 13.4 Hz, 4H), 3.85 (t, *J* = 7.41 Hz, 8H), 4.37 (d, *J* = 13.4 Hz, 4H), 6.81 (s, 8H); ^{13}C NMR ($CDCl_3$) δ: 11.07, 23.82, 31.36, 77.23, 114.97, 130.66, 136.11, 155.02.

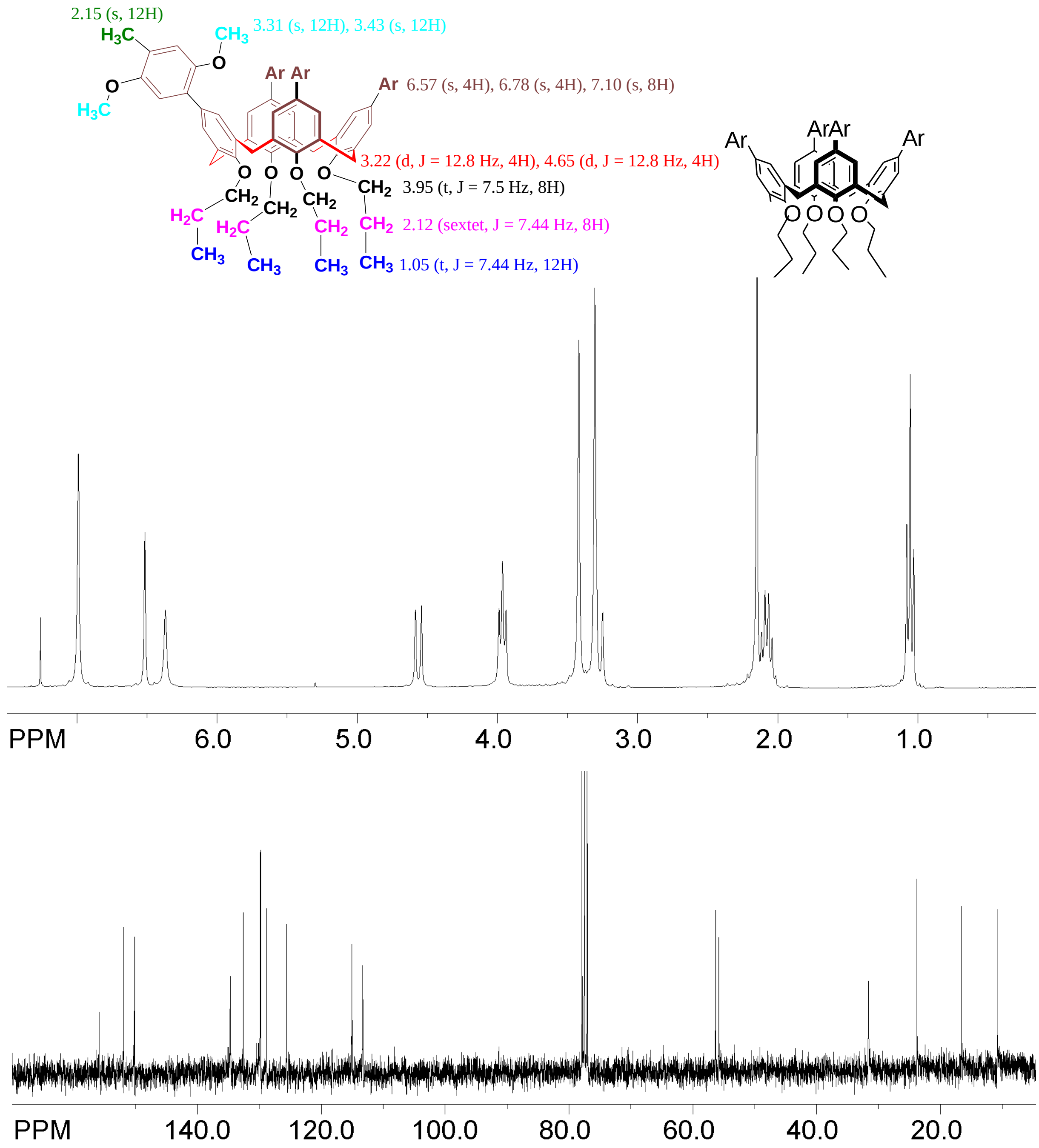

Figure S13: $^{1}H/^{13}C$ NMR spectra data of 5,11,17,23-*tetrakis*(2,5-Dimethoxy-4-methylphenyl)-25,26,27,28-tetrapropoxycalix [4]arene (Cone Conformer) (D) in $CDCl_3$.

^{1}H NMR ($CDCl_3$) δ: 1.05 (t, J = 7.44 Hz, 12H), 2.12 (sextet, J = 7.44 Hz, 8H), 2.15 (s, 12H), 3.22 (d, 4H), 3.31 (s, 12H), 3.43 (s, 12H), 3.95 (t, J = 7.5 Hz, 12H), 4.65 (d, J = 12.8 Hz, 4H), 6.57 (s, 4H), 6.78 (s, 4H), 7.10 (s, 8H); ^{13}C ($CDCl_3$) δ: 11.20, 16.92, 24.50, 31.79, 55.73, 56.25, 112.67, 114.42, 124.90, 128.09, 131.83, 133.90, 149.22, 150.98, 155.46.

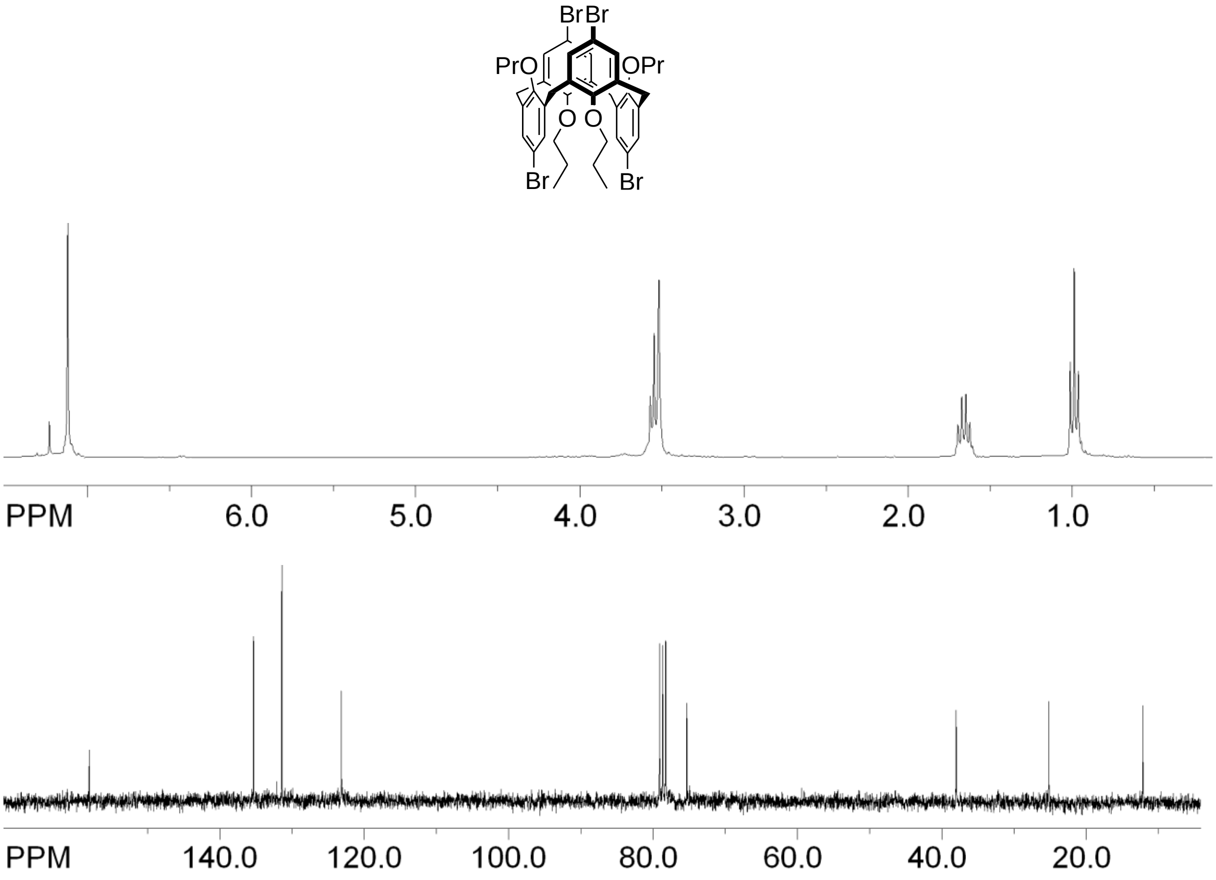

Figure S14: $^{1}H/^{13}C$ NMR spectra data of 5,11,17,23-Tetrabromo-25,26,27,28-tetrapropoxy calix[4]arene (1,3-Alternate Conformer) (17) in $CDCl_3$.

^{1}H NMR ($CDCl_3$) δ: 0.95 (t, *J* = 7.41 Hz, 12H), 1.91 (sextet, *J* = 7.41 Hz, 8H), 3.10 (d, *J* = 13.4 Hz, 4H), 3.85 (t, *J* = 7.41 Hz, 8H), 4.37 (d, *J* = 13.4 Hz, 4H), 6.81 (s, 8H); ^{13}C NMR ($CDCl_3$) δ: 11.07, 23.82, 31.36, 77.23, 114.97, 130.66, 136.11, 155.02.

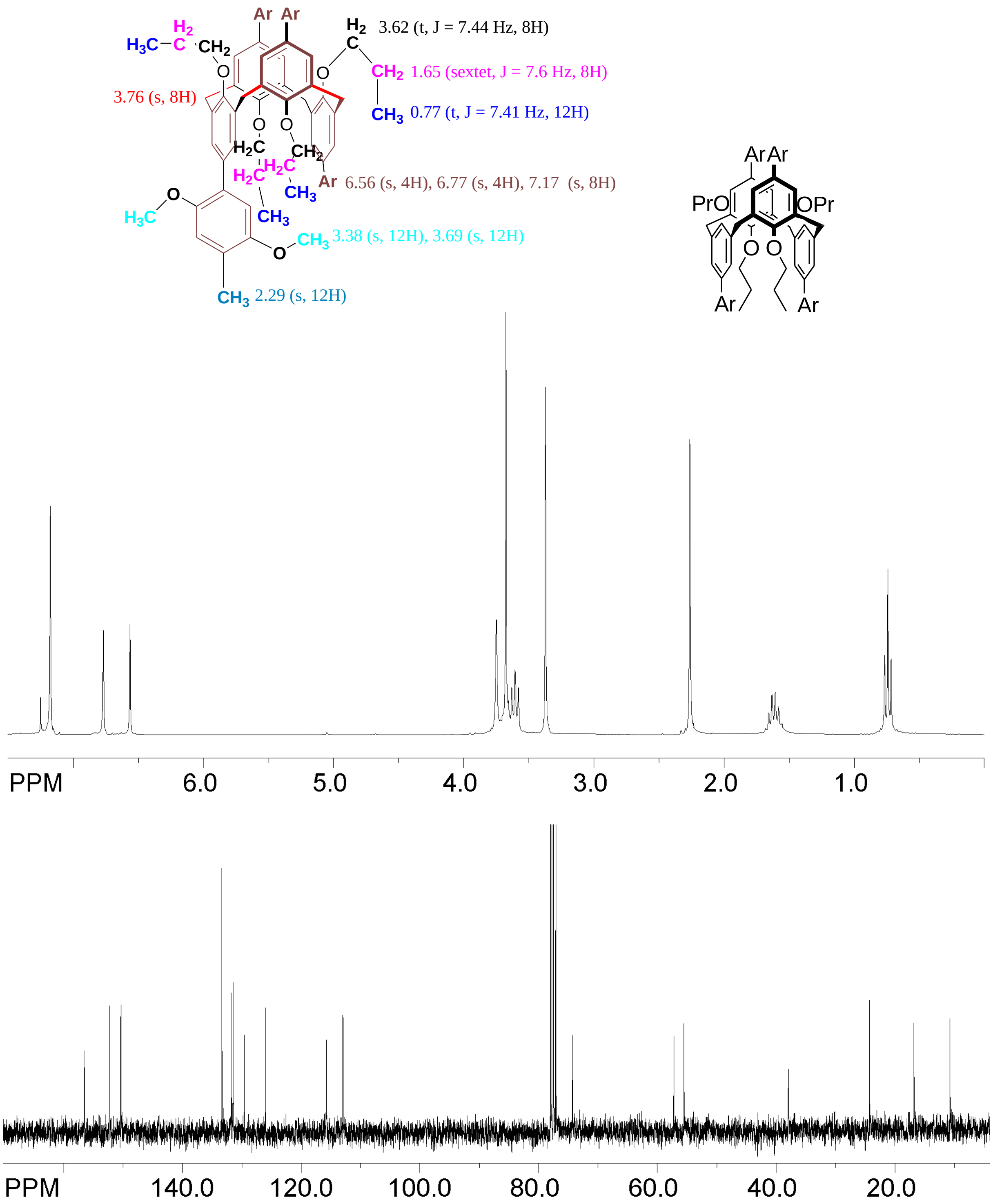

Figure S15: $^1H/^{13}C$ NMR spectra data of 25,26,27,28-tetrapropoxycalix[4]arene (1,3-Alternate Conformer) (E) in $CDCl_3$.

^1H NMR ($CDCl_3$) δ: 0.77 (t, *J* = 7.41 Hz, 12H), 1.65 (sextet, *J* = 7.6 Hz, 8H), 2.29 (s, 12H), 3.38 (s, 12H), 3.62 (t, *J* = 7.44 Hz, 8H), 3.69 (s, 12H), 3.76 (s, 8H), 6.56 (s, 4H), 6.77 (s, 4H), 7.17 (s, 8H); ^{13}C NMR ($CDCl_3$), δ: 11.05, 17.07, 24.50, 37.99, 55.41, 57.12, 74.02, 112.29, 115.03, 125.21, 128.73, 130.65, 130.93, 132.51, 149.34, 151.20, 155.46.

Calixarene – X-Ray

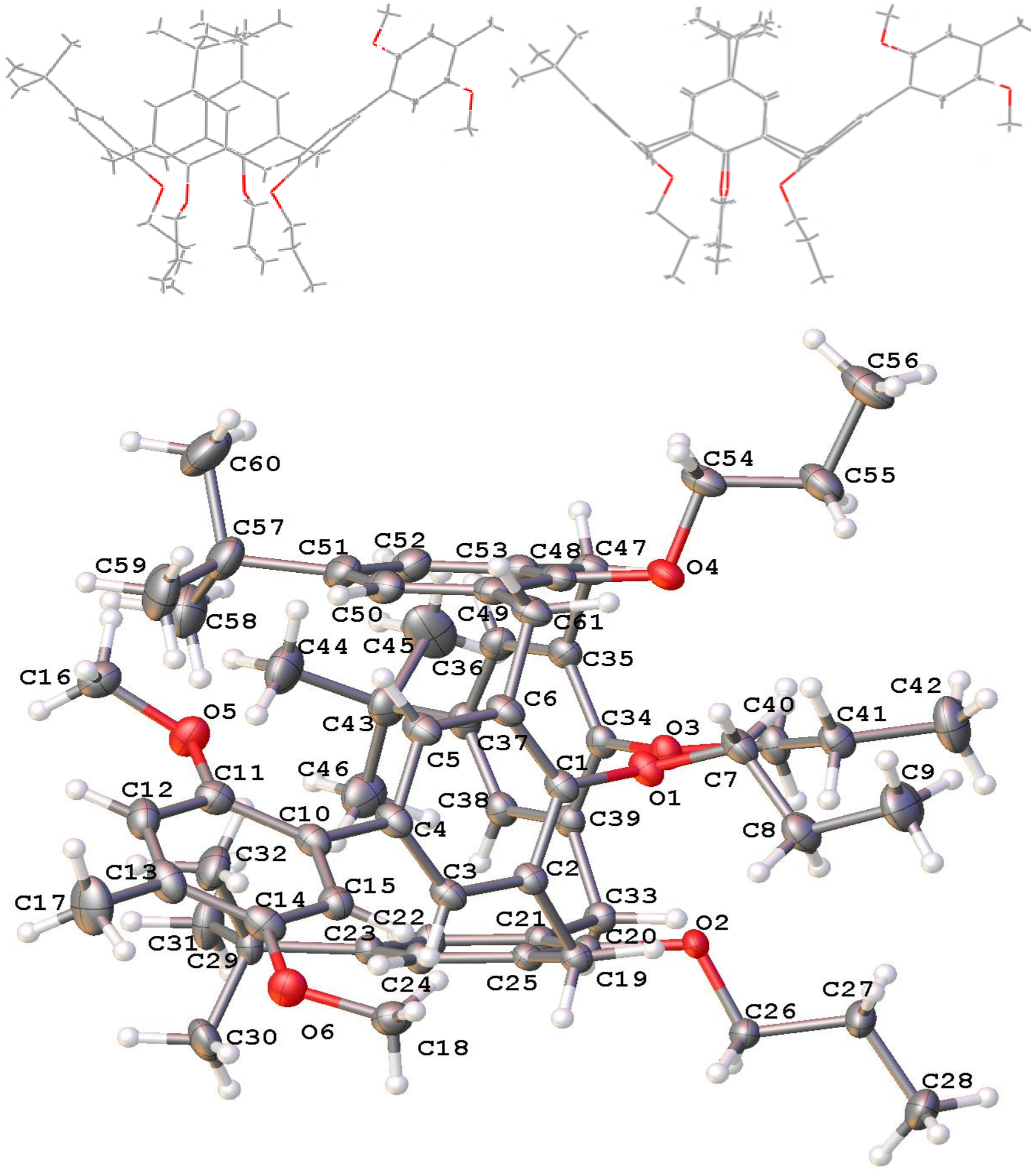

Figure S16: Crystal data and structure refinement for raj_o. 5-(2,5-Dimethoxytoluene)-11,17,23-tri-*p*-*tert*-butyl-25,26,27,28-tetrahydroxy calix[4]arene. (Cone Conformer) (A)

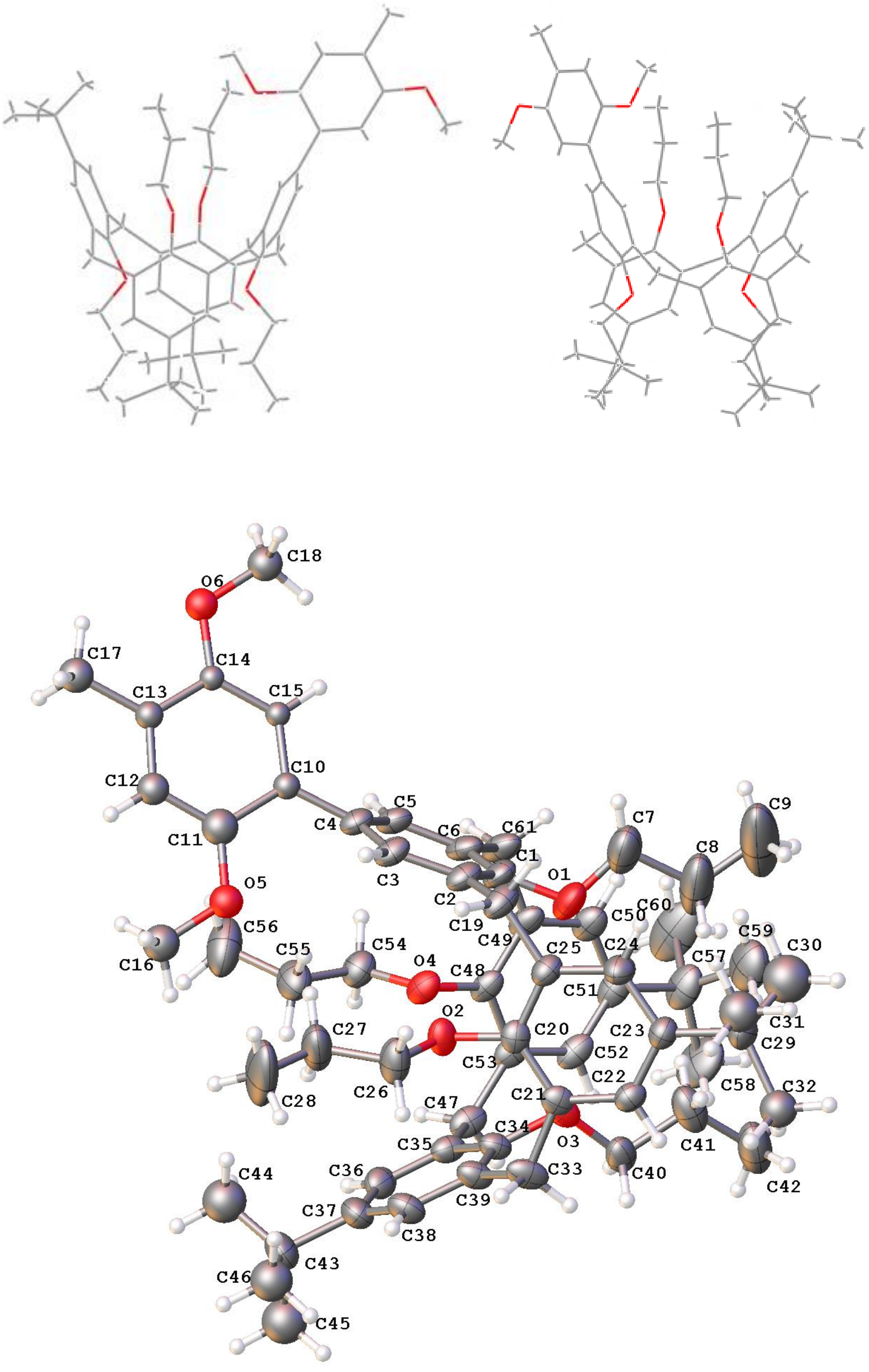

Figure S17: Crystal data and structure refinement for raj_s. 5-(2,5-Dimethoxytoluene)-11,17,23-tri-*p*-*tert*-butyl-25,26,27,28-tetrahydroxy calix[4]arene (1,3-Alternate Conformer) (B).

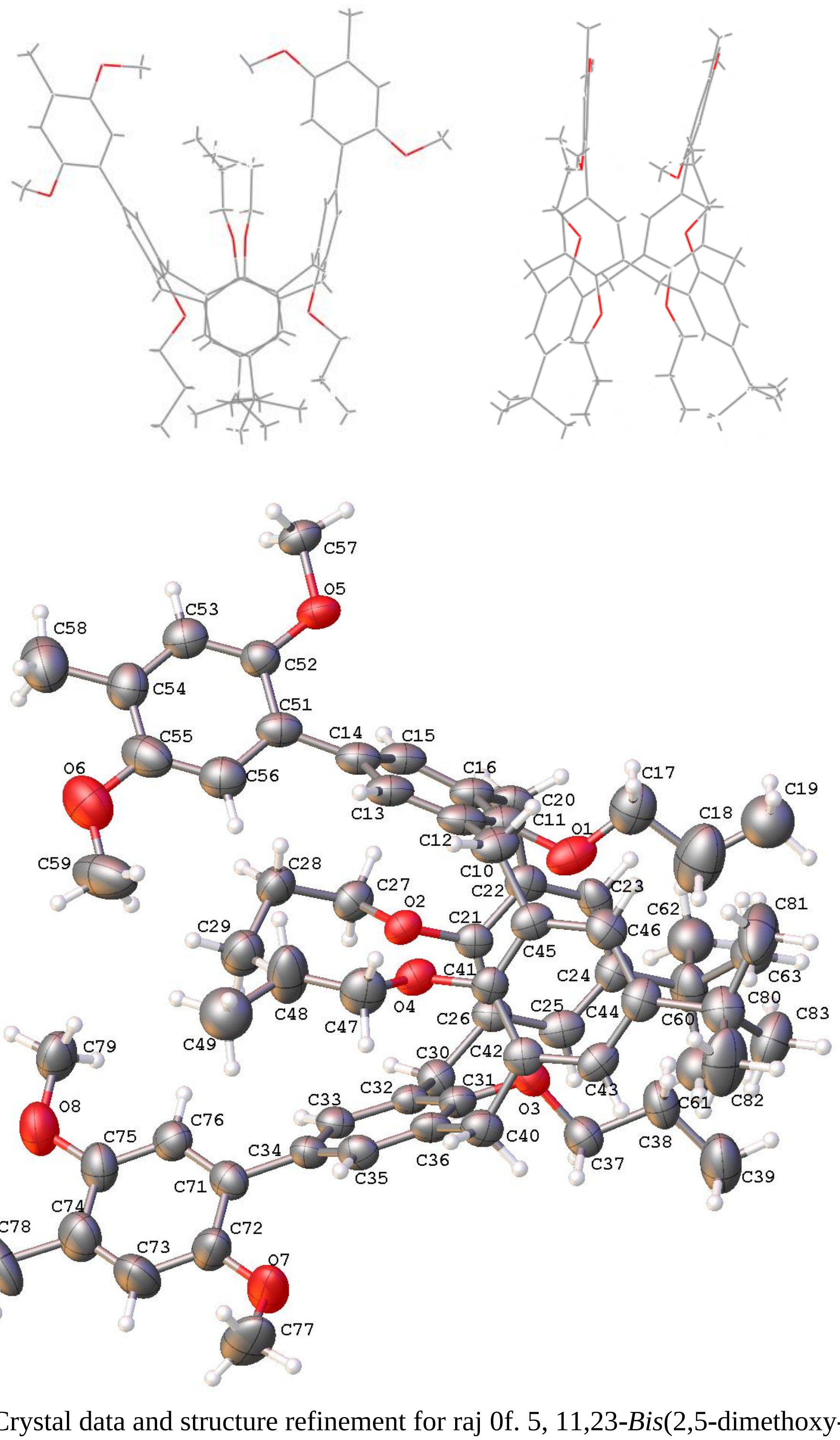

Figure S18: Crystal data and structure refinement for raj 0f. 5, 11,23-*Bis*(2,5-dimethoxy-4-methylphenyl)-5,17-di-*tert*-butyl-25,26,27,28-tetrapropoxycalix[4]arene (1,3-Alternate Conformer) (C).

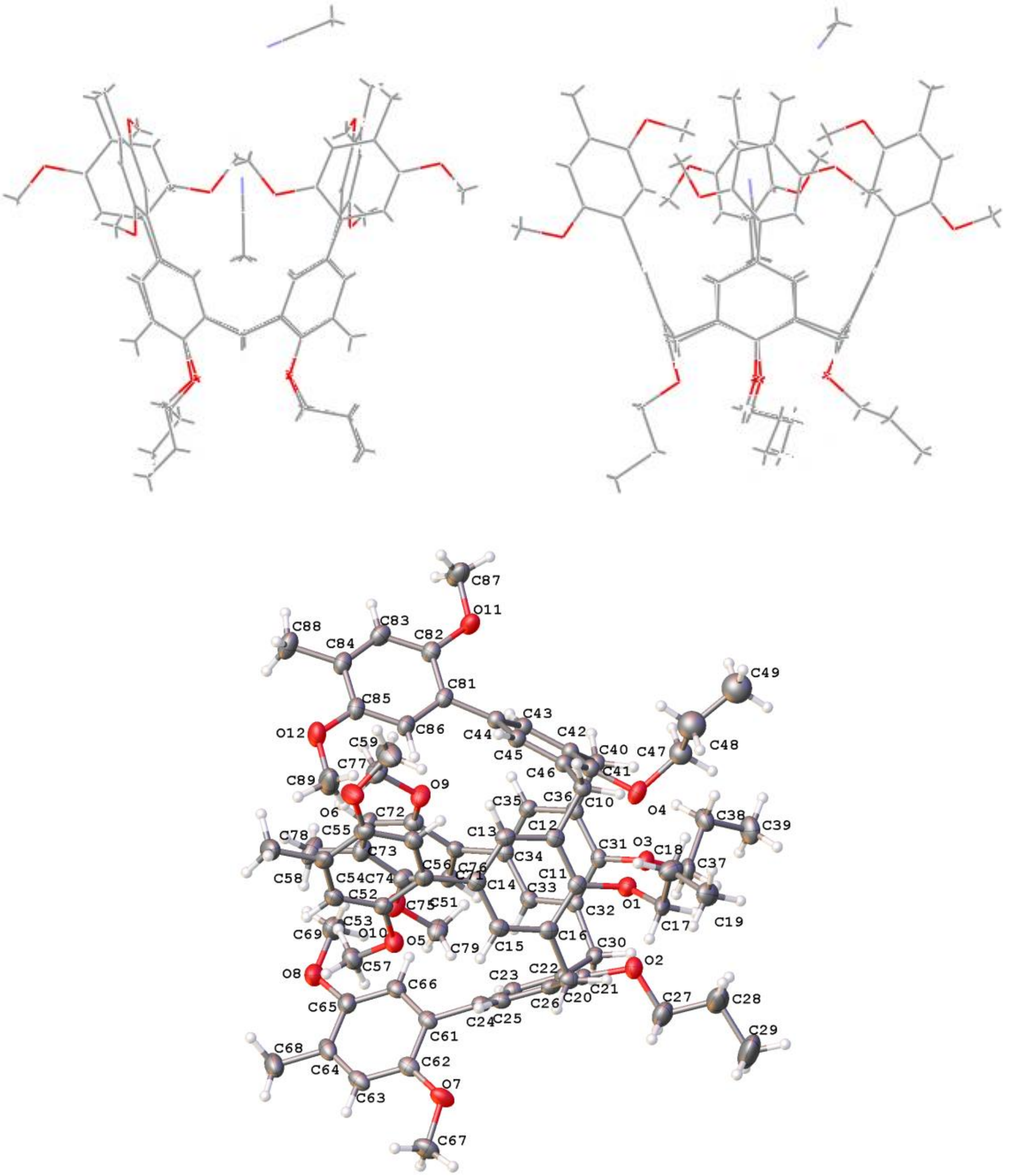

Figure S19: Crystal data and structure refinement for raj0b.5,11,17,23-*tetrakis*(2,5-Dimethoxy-4-methylphenyl)-25,26,27,28-tetrapropoxycalix [4]arene (Cone Conformer) (D).

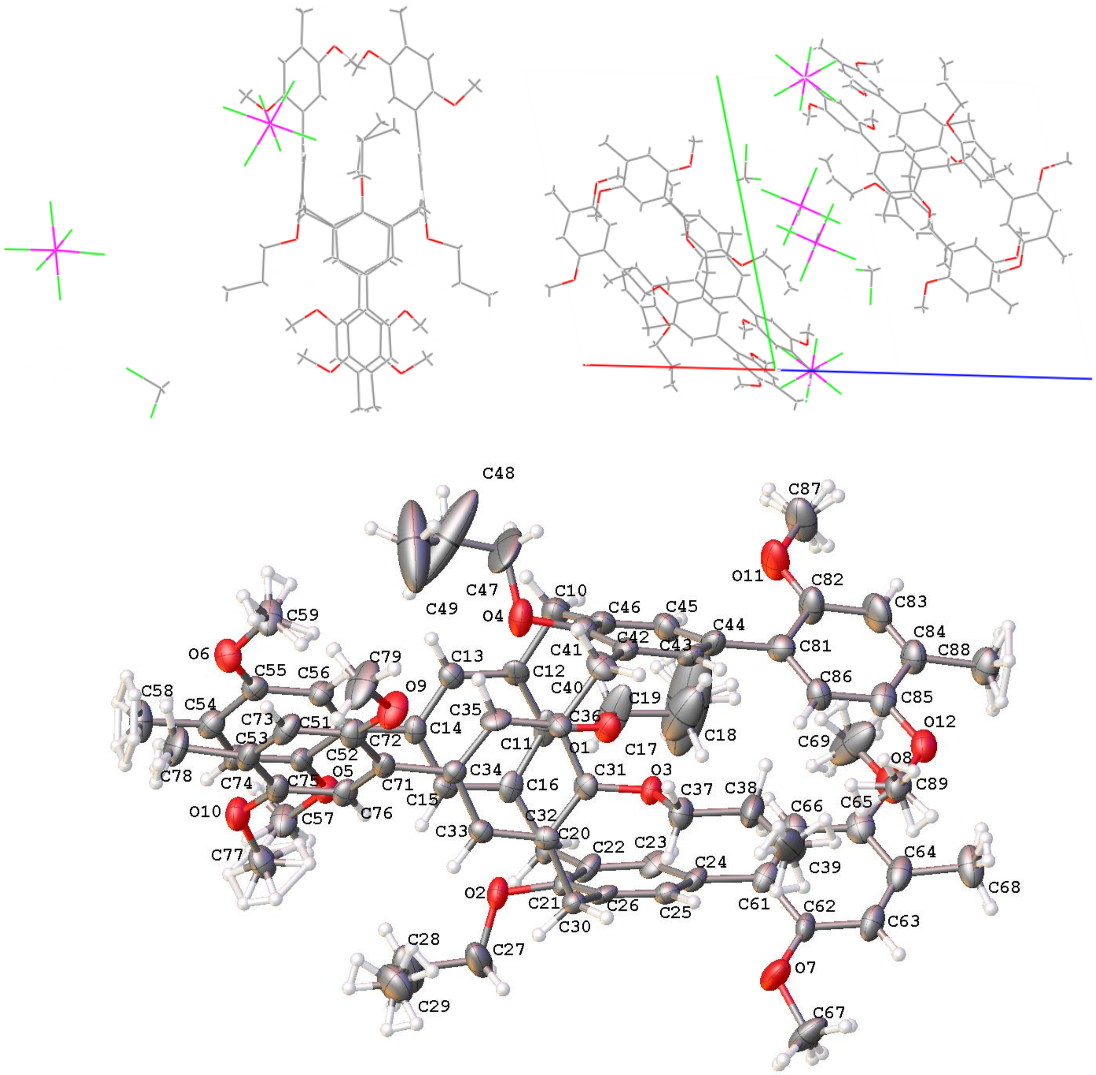

Figure S20: X-ray crystal structure of the cation radical for 1,3-alternate conformer of trataarylcalix[4]arene ether $[E]^{+\cdot}\ SbCl_6^{-}$. raj0ea

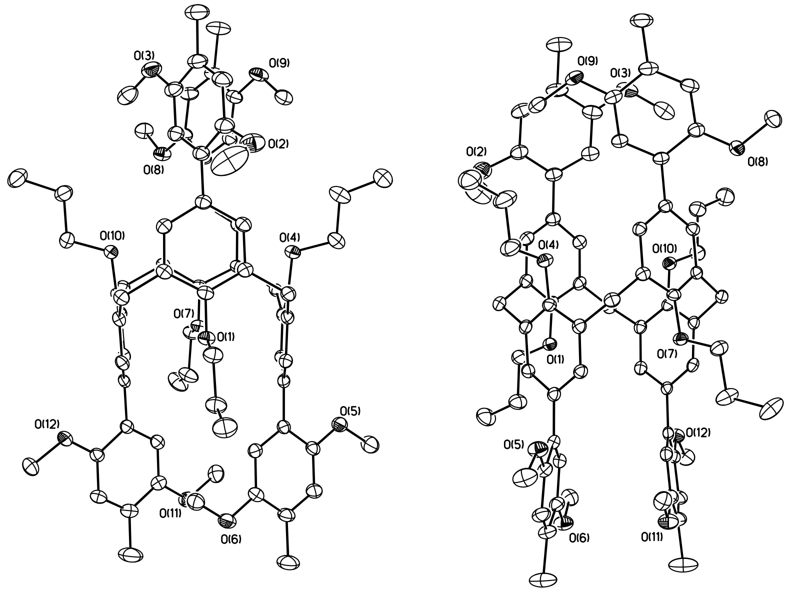

Figure[3] S21: Crystal data and structure refinement 25,26,27,28-tetrapropoxycalix[4]arene (1,3-Alternate Conformer) (E). rethf

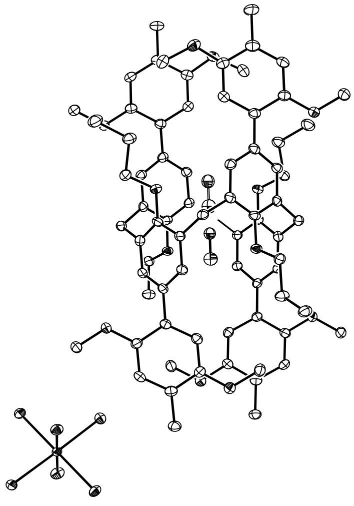

Figure[3] S22: X-ray crystal structure of NO-bound 1,3-alternate conformer of trataarylcalix[4]arene ether $[E,NO]^+$ $]SbCl_6^-$. Hydrogens are omitted for the sake of clarity.

Table S1. Crystal data and structure refinement for raj_o.

Identification code	raj_o	
Empirical formula	C61 H82 O6	
Formula weight	911.27	
Temperature	100(2) K	
Wavelength	0.71073 Å	
Crystal system	Monoclinic	
Space group	P2(1)/c	
Unit cell dimensions	a = 10.355(2) Å	α= 90°.
	b = 25.912(5) Å	β= 90.004(3)°.
	c = 20.403(4) Å	γ = 90°.
Volume	5475(3) $Å^3$	
Z	4	
Density (calculated)	1.106 Mg/m^3	
Absorption coefficient	0.069 mm^{-1}	
F(000)	1984	
Crystal size	0.45 x 0.32 x 0.20 mm^3	
Theta range for data collection	1.57 to 30.59°.	
Index ranges	-14<=h<=14, 0<=k<=37, 0<=l<=29	
Reflections collected	85666	
Independent reflections	16751 [R(int) = 0.0323]	
Completeness to theta = 30.59°	99.6 %	
Absorption correction	Semi-empirical from equivalents	
Max. and min. transmission	0.9863 and 0.9695	
Refinement method	Full-matrix least-squares on F^2	
Data / restraints / parameters	16751 / 6 / 633	
Goodness-of-fit on F^2	1.035	
Final R indices [I>2sigma(I)]	R1 = 0.0516, wR2 = 0.1369	
R indices (all data)	R1 = 0.0668, wR2 = 0.1457	
Largest diff. peak and hole	0.432 and -0.281 e.$Å^{-3}$	

Table S2. Atomic coordinates (x 10^4) and equivalent isotropic displacement parameters (Å^{2}x 10^3) for raj_o. U(eq) is defined as one third of the trace of the orthogonalized U^{ij} tensor.

	x	y	z	U(eq)
O(1)	7085(1)	749(1)	2180(1)	23(1)
O(2)	3876(1)	930(1)	1899(1)	19(1)
O(3)	5602(1)	1698(1)	1170(1)	21(1)
O(4)	8729(1)	1381(1)	1199(1)	25(1)
C(1)	7410(1)	756(1)	2838(1)	21(1)
C(2)	6470(1)	640(1)	3307(1)	20(1)
C(3)	6845(1)	625(1)	3964(1)	23(1)
C(4)	8103(1)	746(1)	4162(1)	24(1)
C(5)	8983(1)	904(1)	3682(1)	25(1)
C(6)	8656(1)	909(1)	3020(1)	23(1)
C(7)	7526(1)	291(1)	1849(1)	26(1)
C(8)	6558(1)	-144(1)	1888(1)	31(1)
C(9)	7019(2)	-616(1)	1509(1)	49(1)
C(10)	8493(1)	682(1)	4860(1)	23(1)
C(11)	9183(1)	1055(1)	5210(1)	26(1)
O(5)	9492(1)	1512(1)	4912(1)	31(1)
C(16)	10408(2)	1839(1)	5221(1)	32(1)
C(15A)	9183(1)	1055(1)	5210(1)	26(1)
C(12)	9529(1)	962(1)	5863(1)	30(1)
C(14A)	9529(1)	962(1)	5863(1)	30(1)
O(6A)	10205(7)	1447(3)	6064(3)	44(2)
C(18A)	10755(10)	1830(4)	5644(5)	42(2)
C(13)	9210(1)	504(1)	6176(1)	31(1)
C(14)	8520(1)	137(1)	5826(1)	28(1)
O(6)	8189(1)	-319(1)	6146(1)	28(1)
C(18)	7692(1)	-730(1)	5763(1)	29(1)
C(12A)	8520(1)	137(1)	5826(1)	28(1)
C(15)	8177(1)	224(1)	5180(1)	24(1)
C(11A)	8177(1)	224(1)	5180(1)	24(1)
O(5A)	7600(6)	-201(2)	4958(3)	34(1)
C(16A)	7635(8)	-678(3)	5309(4)	31(2)

C(17)	9577(2)	391(1)	6879(1)	49(1)
C(19)	5065(1)	598(1)	3109(1)	20(1)
C(20)	3973(1)	1287(1)	2405(1)	17(1)
C(21)	3593(1)	1798(1)	2304(1)	18(1)
C(22)	3743(1)	2153(1)	2814(1)	20(1)
C(23)	4272(1)	2016(1)	3416(1)	19(1)
C(24)	4647(1)	1503(1)	3498(1)	19(1)
C(25)	4519(1)	1137(1)	3004(1)	17(1)
C(26)	2702(1)	642(1)	1910(1)	22(1)
C(27)	2748(1)	256(1)	1356(1)	32(1)
C(28)	1492(1)	-45(1)	1286(1)	39(1)
C(29)	4522(1)	2403(1)	3968(1)	24(1)
C(30)	3687(1)	2266(1)	4563(1)	35(1)
C(31)	4218(2)	2956(1)	3763(1)	38(1)
C(32)	5960(1)	2372(1)	4161(1)	37(1)
C(33)	3147(1)	1998(1)	1640(1)	20(1)
C(34)	5334(1)	2220(1)	1183(1)	20(1)
C(35)	6312(1)	2570(1)	1024(1)	22(1)
C(36)	6036(1)	3095(1)	1053(1)	25(1)
C(37)	4829(1)	3282(1)	1249(1)	24(1)
C(38)	3885(1)	2920(1)	1416(1)	22(1)
C(39)	4120(1)	2388(1)	1388(1)	20(1)
C(40)	5330(1)	1469(1)	546(1)	25(1)
C(41)	5865(1)	928(1)	542(1)	27(1)
C(42)	5704(2)	677(1)	-126(1)	48(1)
C(43)	4623(1)	3866(1)	1301(1)	30(1)
C(44)	5523(2)	4079(1)	1835(1)	42(1)
C(45)	4975(2)	4123(1)	642(1)	50(1)
C(46)	3237(1)	4008(1)	1474(1)	40(1)
C(47)	7668(1)	2380(1)	883(1)	25(1)
C(48)	8795(1)	1765(1)	1674(1)	23(1)
C(49)	9272(1)	1648(1)	2299(1)	24(1)
C(50)	9408(1)	2048(1)	2751(1)	28(1)
C(51)	9081(1)	2555(1)	2604(1)	29(1)
C(52)	8535(1)	2650(1)	1989(1)	29(1)
C(53)	8370(1)	2264(1)	1523(1)	24(1)

C(54)	9833(1)	1382(1)	777(1)	30(1)
C(55)	9592(1)	1011(1)	220(1)	38(1)
C(56)	10796(2)	934(1)	-200(1)	48(1)
C(57)	9357(1)	3001(1)	3077(1)	39(1)
C(58)	8229(2)	3377(1)	3110(1)	51(1)
C(59)	9633(2)	2808(1)	3775(1)	51(1)
C(60)	10564(2)	3282(1)	2821(1)	55(1)
C(61)	9605(1)	1103(1)	2509(1)	26(1)

Table S3. Bond lengths [Å] and angles [°] for raj_o.

O(1)-C(1)	1.3826(12)
O(1)-C(7)	1.4394(14)
O(2)-C(20)	1.3891(12)
O(2)-C(26)	1.4269(13)
O(3)-C(34)	1.3827(13)
O(3)-C(40)	1.4324(13)
O(4)-C(48)	1.3904(14)
O(4)-C(54)	1.4314(13)
C(1)-C(2)	1.3985(15)
C(1)-C(6)	1.3993(16)
C(2)-C(3)	1.3973(14)
C(2)-C(19)	1.5134(15)
C(3)-C(4)	1.3985(16)
C(4)-C(5)	1.3993(16)
C(4)-C(10)	1.4892(15)
C(5)-C(6)	1.3924(15)
C(6)-C(61)	1.5186(15)
C(7)-C(8)	1.5087(18)
C(8)-C(9)	1.5238(18)
C(10)-C(15)	1.3923(16)
C(10)-C(11)	1.3988(17)
C(11)-O(5)	1.3680(15)
C(11)-C(12)	1.4008(17)
O(5)-C(16)	1.4193(18)
C(12)-C(13)	1.3878(19)
O(6A)-C(18A)	1.430(11)
C(13)-C(14)	1.3880(19)
C(13)-C(17)	1.5132(18)
C(14)-C(15)	1.3844(16)
C(14)-O(6)	1.3930(15)
O(6)-C(18)	1.4170(18)
O(5A)-C(16A)	1.429(9)
C(19)-C(25)	1.5208(14)
C(20)-C(21)	1.3970(14)

C(20)-C(25)	1.4019(14)
C(21)-C(22)	1.3974(14)
C(21)-C(33)	1.5227(14)
C(22)-C(23)	1.3913(14)
C(23)-C(24)	1.3943(15)
C(23)-C(29)	1.5295(14)
C(24)-C(25)	1.3895(14)
C(26)-C(27)	1.5105(15)
C(27)-C(28)	1.5220(18)
C(29)-C(31)	1.5275(17)
C(29)-C(30)	1.5313(17)
C(29)-C(32)	1.5424(18)
C(33)-C(39)	1.5179(15)
C(34)-C(39)	1.3949(15)
C(34)-C(35)	1.3967(15)
C(35)-C(36)	1.3913(16)
C(35)-C(47)	1.5159(16)
C(36)-C(37)	1.3986(17)
C(37)-C(38)	1.3972(16)
C(37)-C(43)	1.5328(16)
C(38)-C(39)	1.3991(15)
C(40)-C(41)	1.5069(16)
C(41)-C(42)	1.5192(18)
C(43)-C(46)	1.523(2)
C(43)-C(44)	1.5358(19)
C(43)-C(45)	1.543(2)
C(47)-C(53)	1.5255(16)
C(48)-C(49)	1.3998(15)
C(48)-C(53)	1.3997(17)
C(49)-C(50)	1.3960(17)
C(49)-C(61)	1.5157(17)
C(50)-C(51)	1.3883(18)
C(51)-C(52)	1.3986(17)
C(51)-C(57)	1.5329(18)
C(52)-C(53)	1.3896(17)
C(54)-C(55)	1.5090(19)

C(55)-C(56)	1.5261(19)
C(57)-C(58)	1.523(2)
C(57)-C(59)	1.536(2)
C(57)-C(60)	1.539(2)

C(1)-O(1)-C(7)	112.93(8)
C(20)-O(2)-C(26)	113.48(8)
C(34)-O(3)-C(40)	112.46(8)
C(48)-O(4)-C(54)	112.30(9)
O(1)-C(1)-C(2)	119.44(9)
O(1)-C(1)-C(6)	119.11(9)
C(2)-C(1)-C(6)	121.35(10)
C(3)-C(2)-C(1)	118.00(10)
C(3)-C(2)-C(19)	121.42(9)
C(1)-C(2)-C(19)	120.15(9)
C(2)-C(3)-C(4)	122.00(10)
C(3)-C(4)-C(5)	118.03(10)
C(3)-C(4)-C(10)	120.23(10)
C(5)-C(4)-C(10)	121.69(10)
C(6)-C(5)-C(4)	121.54(11)
C(5)-C(6)-C(1)	118.68(10)
C(5)-C(6)-C(61)	120.72(10)
C(1)-C(6)-C(61)	120.51(10)
O(1)-C(7)-C(8)	112.37(9)
C(7)-C(8)-C(9)	111.40(11)
C(15)-C(10)-C(11)	118.02(10)
C(15)-C(10)-C(4)	118.62(10)
C(11)-C(10)-C(4)	123.35(11)
O(5)-C(11)-C(10)	119.41(11)
O(5)-C(11)-C(12)	120.79(11)
C(10)-C(11)-C(12)	119.80(11)
C(11)-O(5)-C(16)	118.35(11)
C(13)-C(12)-C(11)	121.63(11)
C(12)-C(13)-C(14)	118.18(11)
C(12)-C(13)-C(17)	122.82(13)
C(14)-C(13)-C(17)	119.00(13)

C(15)-C(14)-C(13)	120.64(11)
C(15)-C(14)-O(6)	121.50(11)
C(13)-C(14)-O(6)	117.86(11)
C(14)-O(6)-C(18)	117.94(10)
C(14)-C(15)-C(10)	121.72(11)
C(2)-C(19)-C(25)	109.23(8)
O(2)-C(20)-C(21)	119.98(9)
O(2)-C(20)-C(25)	119.60(9)
C(21)-C(20)-C(25)	120.33(9)
C(20)-C(21)-C(22)	118.87(9)
C(20)-C(21)-C(33)	122.64(9)
C(22)-C(21)-C(33)	118.20(9)
C(23)-C(22)-C(21)	122.24(10)
C(22)-C(23)-C(24)	117.25(9)
C(22)-C(23)-C(29)	123.37(9)
C(24)-C(23)-C(29)	119.29(9)
C(25)-C(24)-C(23)	122.55(9)
C(24)-C(25)-C(20)	118.74(9)
C(24)-C(25)-C(19)	119.33(9)
C(20)-C(25)-C(19)	121.76(9)
O(2)-C(26)-C(27)	107.99(9)
C(26)-C(27)-C(28)	112.48(10)
C(31)-C(29)-C(23)	112.24(9)
C(31)-C(29)-C(30)	108.58(11)
C(23)-C(29)-C(30)	109.65(10)
C(31)-C(29)-C(32)	108.47(11)
C(23)-C(29)-C(32)	108.53(9)
C(30)-C(29)-C(32)	109.33(10)
C(39)-C(33)-C(21)	109.02(8)
O(3)-C(34)-C(39)	119.49(9)
O(3)-C(34)-C(35)	119.03(10)
C(39)-C(34)-C(35)	121.37(10)
C(36)-C(35)-C(34)	118.39(10)
C(36)-C(35)-C(47)	121.04(10)
C(34)-C(35)-C(47)	120.37(10)
C(35)-C(36)-C(37)	122.28(10)

C(38)-C(37)-C(36)	117.55(10)
C(38)-C(37)-C(43)	123.29(11)
C(36)-C(37)-C(43)	119.09(10)
C(37)-C(38)-C(39)	121.95(10)
C(34)-C(39)-C(38)	118.43(10)
C(34)-C(39)-C(33)	119.44(9)
C(38)-C(39)-C(33)	121.83(10)
O(3)-C(40)-C(41)	108.48(9)
C(40)-C(41)-C(42)	111.31(11)
C(46)-C(43)-C(37)	112.73(10)
C(46)-C(43)-C(44)	108.69(11)
C(37)-C(43)-C(44)	108.62(10)
C(46)-C(43)-C(45)	108.63(12)
C(37)-C(43)-C(45)	109.47(10)
C(44)-C(43)-C(45)	108.62(13)
C(35)-C(47)-C(53)	109.99(9)
O(4)-C(48)-C(49)	119.80(10)
O(4)-C(48)-C(53)	119.45(10)
C(49)-C(48)-C(53)	120.75(11)
C(50)-C(49)-C(48)	118.47(11)
C(50)-C(49)-C(61)	118.81(10)
C(48)-C(49)-C(61)	122.70(11)
C(51)-C(50)-C(49)	122.31(11)
C(50)-C(51)-C(52)	117.37(11)
C(50)-C(51)-C(57)	122.04(11)
C(52)-C(51)-C(57)	120.53(12)
C(53)-C(52)-C(51)	122.41(12)
C(52)-C(53)-C(48)	118.41(10)
C(52)-C(53)-C(47)	120.10(11)
C(48)-C(53)-C(47)	121.39(10)
O(4)-C(54)-C(55)	108.65(10)
C(54)-C(55)-C(56)	111.79(13)
C(58)-C(57)-C(51)	111.60(11)
C(58)-C(57)-C(59)	108.07(14)
C(51)-C(57)-C(59)	111.95(12)
C(58)-C(57)-C(60)	109.50(14)

C(51)-C(57)-C(60)	107.14(13)
C(59)-C(57)-C(60)	108.53(13)
C(49)-C(61)-C(6)	110.84(9)

Symmetry transformations used to generate equivalent atoms:

Table S4. Anisotropic displacement parameters ($Å^2$x 10^3)for raj_o. The anisotropic displacement factor exponent takes the form: $-2\pi^2[h^2a^{*2}U^{11} + ... + 2 h k a^* b^* U^{12}]$

	U^{11}	U^{22}	U^{33}	U^{23}	U^{13}	U^{12}
O(1)	25(1)	27(1)	16(1)	1(1)	-1(1)	7(1)
O(2)	20(1)	22(1)	16(1)	-6(1)	2(1)	-1(1)
O(3)	28(1)	20(1)	16(1)	1(1)	-1(1)	3(1)
O(4)	24(1)	33(1)	19(1)	-1(1)	4(1)	1(1)
C(1)	22(1)	24(1)	16(1)	1(1)	-1(1)	7(1)
C(2)	20(1)	22(1)	19(1)	0(1)	-1(1)	5(1)
C(3)	23(1)	27(1)	18(1)	2(1)	2(1)	5(1)
C(4)	24(1)	31(1)	18(1)	1(1)	-1(1)	7(1)
C(5)	20(1)	37(1)	20(1)	1(1)	-1(1)	6(1)
C(6)	20(1)	29(1)	19(1)	2(1)	1(1)	7(1)
C(7)	28(1)	32(1)	18(1)	-1(1)	2(1)	8(1)
C(8)	36(1)	28(1)	30(1)	-3(1)	6(1)	7(1)
C(9)	56(1)	39(1)	52(1)	-17(1)	8(1)	10(1)
C(10)	22(1)	30(1)	18(1)	1(1)	-1(1)	5(1)
C(11)	26(1)	26(1)	26(1)	2(1)	2(1)	2(1)
O(5)	39(1)	28(1)	25(1)	6(1)	-6(1)	-6(1)
C(16)	29(1)	31(1)	36(1)	2(1)	-2(1)	-3(1)
C(15A)	26(1)	26(1)	26(1)	2(1)	2(1)	2(1)
C(12)	31(1)	33(1)	27(1)	-9(1)	-6(1)	4(1)
C(14A)	31(1)	33(1)	27(1)	-9(1)	-6(1)	4(1)
C(13)	36(1)	39(1)	20(1)	-1(1)	-4(1)	12(1)
C(14)	31(1)	29(1)	23(1)	5(1)	3(1)	8(1)
O(6)	35(1)	27(1)	22(1)	5(1)	-3(1)	-1(1)
C(18)	28(1)	31(1)	29(1)	2(1)	1(1)	-3(1)
C(12A)	31(1)	29(1)	23(1)	5(1)	3(1)	8(1)
C(15)	24(1)	27(1)	22(1)	-1(1)	-1(1)	2(1)
C(11A)	24(1)	27(1)	22(1)	-1(1)	-1(1)	2(1)
C(17)	75(1)	46(1)	25(1)	1(1)	-15(1)	7(1)
C(19)	21(1)	20(1)	20(1)	0(1)	0(1)	2(1)
C(20)	17(1)	20(1)	14(1)	-3(1)	1(1)	-1(1)
C(21)	18(1)	21(1)	15(1)	0(1)	0(1)	0(1)

C(22)	24(1)	18(1)	18(1)	-1(1)	0(1)	2(1)
C(23)	21(1)	21(1)	16(1)	-2(1)	0(1)	-1(1)
C(24)	21(1)	22(1)	15(1)	-1(1)	-2(1)	1(1)
C(25)	17(1)	19(1)	17(1)	0(1)	2(1)	2(1)
C(26)	21(1)	24(1)	20(1)	-4(1)	1(1)	-3(1)
C(27)	28(1)	35(1)	31(1)	-16(1)	0(1)	-3(1)
C(28)	42(1)	40(1)	36(1)	-14(1)	-1(1)	-14(1)
C(29)	31(1)	23(1)	18(1)	-6(1)	-3(1)	1(1)
C(30)	46(1)	40(1)	21(1)	-9(1)	5(1)	0(1)
C(31)	60(1)	23(1)	31(1)	-8(1)	-11(1)	2(1)
C(32)	35(1)	38(1)	37(1)	-14(1)	-10(1)	-2(1)
C(33)	22(1)	23(1)	16(1)	1(1)	-2(1)	0(1)
C(34)	26(1)	20(1)	14(1)	1(1)	-1(1)	1(1)
C(35)	25(1)	25(1)	16(1)	2(1)	1(1)	-1(1)
C(36)	30(1)	23(1)	21(1)	2(1)	0(1)	-4(1)
C(37)	33(1)	20(1)	19(1)	0(1)	-1(1)	0(1)
C(38)	27(1)	22(1)	18(1)	0(1)	0(1)	2(1)
C(39)	24(1)	21(1)	14(1)	1(1)	-2(1)	0(1)
C(40)	35(1)	24(1)	17(1)	-1(1)	-3(1)	4(1)
C(41)	31(1)	24(1)	25(1)	-1(1)	3(1)	5(1)
C(42)	74(1)	35(1)	34(1)	-13(1)	1(1)	9(1)
C(43)	40(1)	20(1)	30(1)	-1(1)	0(1)	0(1)
C(44)	48(1)	28(1)	52(1)	-14(1)	-8(1)	-2(1)
C(45)	81(1)	27(1)	44(1)	11(1)	11(1)	5(1)
C(46)	41(1)	25(1)	52(1)	-5(1)	-1(1)	5(1)
C(47)	24(1)	31(1)	21(1)	4(1)	2(1)	-1(1)
C(48)	18(1)	32(1)	19(1)	0(1)	2(1)	-2(1)
C(49)	17(1)	34(1)	21(1)	2(1)	1(1)	-1(1)
C(50)	23(1)	38(1)	22(1)	1(1)	-3(1)	-3(1)
C(51)	26(1)	34(1)	28(1)	-2(1)	-3(1)	-8(1)
C(52)	27(1)	29(1)	30(1)	2(1)	-2(1)	-5(1)
C(53)	20(1)	32(1)	21(1)	3(1)	1(1)	-3(1)
C(54)	23(1)	43(1)	23(1)	4(1)	6(1)	6(1)
C(55)	34(1)	55(1)	25(1)	-5(1)	7(1)	7(1)
C(56)	39(1)	76(1)	30(1)	-4(1)	10(1)	15(1)
C(57)	41(1)	39(1)	37(1)	-6(1)	-10(1)	-12(1)

C(58)	61(1)	43(1)	50(1)	-16(1)	-6(1)	-3(1)
C(59)	71(1)	49(1)	35(1)	-9(1)	-17(1)	-12(1)
C(60)	51(1)	52(1)	62(1)	-3(1)	-10(1)	-25(1)
C(61)	20(1)	35(1)	22(1)	2(1)	2(1)	6(1)

Table S5. Hydrogen coordinates (x 10^4) and isotropic displacement parameters ($Å^2$x 10^3) for raj_o.

	x	y	z	U(eq)
H(3A)	6227	531	4287	27
H(5A)	9823	1011	3811	30
H(7A)	8349	176	2048	31
H(7B)	7693	373	1383	31
H(8A)	5722	-26	1706	38
H(8B)	6421	-239	2353	38
H(9A)	6373	-891	1543	74
H(9B)	7840	-736	1692	74
H(9C)	7140	-524	1047	74
H(16A)	10534	2150	4955	48
H(16B)	11232	1655	5265	48
H(16C)	10092	1936	5656	48
H(15A)	9415	1371	5005	31
H(12)	9995	1219	6097	36
H(18A)	11123	2108	5910	63
H(18B)	10083	1971	5357	63
H(18C)	11437	1673	5377	63
H(18D)	7504	-1026	6048	44
H(18E)	8331	-831	5433	44
H(18F)	6896	-618	5545	44
H(12A)	8282	-178	6032	33
H(15)	7715	-35	4948	29
H(16D)	7159	-941	5063	47
H(16E)	7238	-632	5740	47
H(16F)	8534	-788	5363	47
H(17A)	10084(13)	658(5)	6948(6)	14(3)
H(17B)	10040(30)	67(11)	6950(13)	97(8)
H(17C)	8810(30)	391(13)	7203(16)	129(11)
H(19A)	4571	419	3457	24
H(19B)	4988	395	2700	24

H(22A)	3474	2499	2747	24
H(24A)	5004	1400	3906	23
H(26A)	1954	876	1854	26
H(26B)	2610	461	2334	26
H(27A)	3464	10	1435	38
H(27B)	2928	440	941	38
H(28A)	1570	-291	923	59
H(28B)	783	195	1196	59
H(28C)	1316	-232	1693	59
H(30A)	2773	2286	4442	53
H(30B)	3864	2509	4919	53
H(30C)	3891	1914	4708	53
H(31A)	3306	2982	3639	57
H(31B)	4760	3052	3388	57
H(31C)	4392	3190	4130	57
H(32A)	6496	2461	3781	55
H(32B)	6165	2021	4307	55
H(32C)	6133	2616	4518	55
H(33A)	3073	1707	1326	24
H(33B)	2288	2162	1683	24
H(36A)	6691	3335	936	29
H(38A)	3059	3038	1553	27
H(40A)	5735	1675	192	30
H(40B)	4386	1461	469	30
H(41A)	5411	719	876	32
H(41B)	6793	938	658	32
H(42A)	6059	327	-114	72
H(42B)	6165	880	-457	72
H(42C)	4785	662	-239	72
H(44A)	5307	3920	2256	64
H(44B)	5415	4454	1866	64
H(44C)	6421	3999	1722	64
H(45A)	4406	3991	297	76
H(45B)	5874	4044	532	76
H(45C)	4867	4498	680	76
H(46A)	3003	3849	1893	59

H(46B)	2655	3883	1130	59
H(46C)	3161	4384	1510	59
H(47A)	8148	2646	635	30
H(47B)	7628	2064	611	30
H(50A)	9736	1971	3175	33
H(52A)	8268	2991	1885	34
H(54A)	10611	1276	1025	35
H(54B)	9980	1734	602	35
H(55A)	8883	1144	-57	46
H(55B)	9317	674	401	46
H(56A)	10604	691	-555	73
H(56B)	11496	796	71	73
H(56C)	11060	1266	-388	73
H(58A)	8039	3507	2670	77
H(58B)	8456	3667	3397	77
H(58C)	7467	3200	3285	77
H(59A)	8871	2628	3946	77
H(59B)	9837	3102	4058	77
H(59C)	10368	2570	3767	77
H(60A)	10400	3409	2376	82
H(60B)	11297	3043	2814	82
H(60C)	10764	3574	3109	82
H(61A)	10490	1097	2693	31
H(61B)	9587	872	2123	31

Table S1. Crystal data and structure refinement for raj_s.

Identification code	raj_s	
Empirical formula	C61 H82 O6	
Formula weight	911.27	
Temperature	100(2) K	
Wavelength	0.71073 Å	
Crystal system	Orthorhombic	
Space group	Pbca	
Unit cell dimensions	a = 10.5301(4) Å	α= 90°.
	b = 19.8639(8) Å	β= 90°.
	c = 52.491(2) Å	γ = 90°.
Volume	10979.5(7) $Å^3$	
Z	8	
Density (calculated)	1.103 Mg/m^3	
Absorption coefficient	0.069 mm^{-1}	
F(000)	3968	
Crystal size	0.40 x 0.40 x 0.17 mm^3	
Theta range for data collection	1.55 to 29.89°.	
Index ranges	0<=h<=14, 0<=k<=27, 0<=l<=72	
Reflections collected	146190	
Independent reflections	15021 [R(int) = 0.0349]	
Completeness to theta = 29.89°	94.7 %	
Absorption correction	Semi-empirical from equivalents	
Max. and min. transmission	0.9884 and 0.9729	
Refinement method	Full-matrix least-squares on F^2	
Data / restraints / parameters	15021 / 25 / 590	
Goodness-of-fit on F^2	1.070	
Final R indices [I>2sigma(I)]	R1 = 0.0933, wR2 = 0.2445	
R indices (all data)	R1 = 0.1218, wR2 = 0.2617	
Largest diff. peak and hole	0.839 and -0.478 e.$Å^{-3}$	

Table S2. Atomic coordinates (x 10^4) and equivalent isotropic displacement parameters (Å^{2}x 10^3) for raj_s. U(eq) is defined as one third of the trace of the orthogonalized U^{ij} tensor.

	x	y	z	U(eq)
O(1)	2389(2)	4492(1)	1654(1)	54(1)
O(2)	2334(2)	3075(1)	1147(1)	40(1)
O(3)	5452(2)	3583(1)	1410(1)	39(1)
O(4)	3929(2)	4887(1)	1040(1)	38(1)
C(1)	1617(2)	4653(1)	1452(1)	40(1)
C(2)	731(2)	4183(1)	1361(1)	38(1)
C(3)	19(2)	4361(1)	1148(1)	37(1)
C(4)	125(2)	4994(1)	1034(1)	34(1)
C(5)	1023(2)	5440(1)	1130(1)	37(1)
C(6)	1794(2)	5275(1)	1334(1)	39(1)
C(7)	1890(4)	4662(2)	1892(1)	76(1)
C(8)	2962(6)	4583(2)	2086(1)	108(2)
C(9)	2614(9)	4716(3)	2342(1)	163(4)
O(5)	6(3)	4332(2)	544(1)	40(1)
O(6)	-3193(3)	6497(2)	722(1)	38(1)
C(10)	-719(6)	5228(3)	839(1)	26(2)
C(11)	-785(10)	4885(5)	588(2)	48(4)
C(12)	-1693(6)	5095(3)	407(1)	37(1)
C(13)	-2467(4)	5639(3)	458(1)	29(1)
C(14)	-2360(4)	5973(2)	691(1)	22(1)
C(15)	-1486(5)	5766(3)	872(1)	22(1)
C(16)	74(6)	4060(3)	293(1)	49(1)
C(17)	-3442(5)	5857(3)	259(1)	44(1)
C(18)	-3199(5)	6804(3)	965(1)	43(1)
O(5A)	-1579(3)	6117(2)	1048(1)	42(1)
O(6A)	-1465(4)	4560(2)	176(1)	64(1)
C(10A)	-702(6)	5163(3)	800(1)	25(2)
C(11A)	-1569(5)	5725(3)	819(1)	31(2)
C(12A)	-2388(4)	5844(3)	616(1)	32(1)
C(13A)	-2369(5)	5441(3)	404(1)	39(1)
C(14A)	-1483(6)	4928(3)	396(1)	45(2)

C(15A)	-723(7)	4801(4)	597(1)	29(2)
C(16A)	-2572(5)	6605(3)	1076(1)	47(1)
C(17A)	-3261(6)	5572(4)	182(1)	61(2)
C(18A)	-486(7)	4081(4)	144(1)	72(2)
C(19)	466(2)	3512(1)	1491(1)	45(1)
C(20)	2492(2)	2882(1)	1398(1)	32(1)
C(21)	3546(2)	2509(1)	1474(1)	32(1)
C(22)	3636(2)	2317(1)	1728(1)	33(1)
C(23)	2729(2)	2487(1)	1909(1)	36(1)
C(24)	1711(2)	2884(1)	1826(1)	39(1)
C(25)	1587(2)	3091(1)	1575(1)	35(1)
C(26)	1777(3)	2567(2)	992(1)	58(1)
C(27)	1464(4)	2871(2)	737(1)	71(1)
C(28)	891(6)	2365(2)	554(1)	118(2)
C(29)	2831(3)	2244(2)	2186(1)	50(1)
C(30)	2816(7)	2792(4)	2367(1)	84(2)
C(31)	1625(5)	1752(3)	2233(1)	60(1)
C(32)	3975(5)	1768(2)	2230(1)	50(1)
C(30A)	2983(10)	1534(5)	2201(2)	77(3)
C(31A)	4025(9)	2630(5)	2311(2)	72(2)
C(32A)	1734(10)	2506(5)	2354(2)	78(3)
C(33)	4583(3)	2279(1)	1292(1)	43(1)
C(34)	5493(2)	3396(1)	1156(1)	32(1)
C(35)	5941(2)	3840(1)	970(1)	34(1)
C(36)	5922(2)	3632(1)	717(1)	41(1)
C(37)	5470(3)	3007(1)	641(1)	45(1)
C(38)	5022(2)	2581(1)	833(1)	44(1)
C(39)	5023(2)	2767(1)	1089(1)	37(1)
C(40)	6615(3)	3499(1)	1549(1)	45(1)
C(41)	6348(4)	3676(2)	1822(1)	62(1)
C(42)	7521(5)	3639(2)	1986(1)	82(1)
C(43)	5480(3)	2798(2)	357(1)	61(1)
C(44)	4734(6)	3240(3)	209(1)	69(2)
C(45)	6874(6)	2710(3)	278(1)	73(2)
C(46)	4903(5)	2045(3)	331(1)	64(1)
C(44A)	6290(8)	2285(4)	297(2)	59(2)

C(45A)	4106(8)	2725(4)	248(2)	54(2)
C(46A)	5874(7)	3466(4)	169(1)	48(2)
C(47)	6519(2)	4519(1)	1030(1)	38(1)
C(48)	4685(2)	5139(1)	1233(1)	34(1)
C(49)	4167(2)	5549(1)	1423(1)	38(1)
C(50)	4986(2)	5791(1)	1610(1)	42(1)
C(51)	6279(2)	5633(1)	1617(1)	42(1)
C(52)	6734(2)	5205(1)	1430(1)	39(1)
C(53)	5955(2)	4951(1)	1238(1)	34(1)
C(54)	3811(2)	5332(1)	826(1)	45(1)
C(55)	3083(3)	4963(2)	622(1)	55(1)
C(56)	2669(4)	5440(3)	408(1)	86(1)
C(57)	7135(3)	5922(1)	1827(1)	55(1)
C(58)	8530(3)	5713(2)	1791(1)	82(1)
C(59)	6686(4)	5665(2)	2083(1)	77(1)
C(60)	7065(4)	6693(2)	1823(1)	93(2)
C(61)	2784(2)	5773(1)	1425(1)	47(1)

Table S3. Bond lengths [Å] and angles [°] for raj_s.

O(1)-C(1)	1.377(3)
O(1)-C(7)	1.394(4)
O(2)-C(20)	1.382(3)
O(2)-C(26)	1.424(3)
O(3)-C(34)	1.385(3)
O(3)-C(40)	1.435(3)
O(4)-C(48)	1.383(3)
O(4)-C(54)	1.436(3)
C(1)-C(6)	1.394(4)
C(1)-C(2)	1.404(3)
C(2)-C(3)	1.390(3)
C(2)-C(19)	1.524(3)
C(3)-C(4)	1.398(3)
C(4)-C(5)	1.390(3)
C(4)-C(10)	1.433(6)
C(4)-C(10A)	1.543(6)
C(5)-C(6)	1.383(3)
C(6)-C(61)	1.515(3)
C(7)-C(8)	1.531(5)
C(8)-C(9)	1.416(7)
O(5)-C(11)	1.399(9)
O(5)-C(16)	1.425(6)
O(6)-C(14)	1.371(5)
O(6)-C(18)	1.413(6)
C(10)-C(15)	1.351(8)
C(10)-C(11)	1.486(11)
C(11)-C(12)	1.409(10)
C(12)-C(13)	1.380(8)
C(13)-C(14)	1.397(6)
C(13)-C(17)	1.527(6)
C(14)-C(15)	1.382(7)
O(5A)-C(16A)	1.433(6)
O(5A)-C(11A)	1.433(6)
O(6A)-C(14A)	1.370(7)

O(6A)-C(18A)	1.412(8)
C(10A)-C(15A)	1.285(9)
C(10A)-C(11A)	1.446(8)
C(11A)-C(12A)	1.388(7)
C(12A)-C(13A)	1.373(7)
C(13A)-C(14A)	1.382(8)
C(13A)-C(17A)	1.517(7)
C(14A)-C(15A)	1.348(9)
C(19)-C(25)	1.512(3)
C(20)-C(21)	1.393(3)
C(20)-C(25)	1.395(3)
C(21)-C(22)	1.389(3)
C(21)-C(33)	1.522(3)
C(22)-C(23)	1.388(3)
C(23)-C(24)	1.400(3)
C(23)-C(29)	1.534(3)
C(24)-C(25)	1.384(3)
C(26)-C(27)	1.504(4)
C(27)-C(28)	1.514(5)
C(29)-C(30A)	1.421(10)
C(29)-C(30)	1.446(7)
C(29)-C(32A)	1.545(10)
C(29)-C(32)	1.549(5)
C(29)-C(31A)	1.614(10)
C(29)-C(31)	1.620(6)
C(33)-C(39)	1.513(3)
C(34)-C(39)	1.389(3)
C(34)-C(35)	1.396(3)
C(35)-C(36)	1.391(3)
C(35)-C(47)	1.513(3)
C(36)-C(37)	1.387(4)
C(37)-C(38)	1.397(4)
C(37)-C(43)	1.547(4)
C(38)-C(39)	1.395(3)
C(40)-C(41)	1.504(4)
C(41)-C(42)	1.507(5)

C(43)-C(44A)	1.366(9)
C(43)-C(44)	1.412(6)
C(43)-C(45)	1.534(7)
C(43)-C(45A)	1.562(9)
C(43)-C(46)	1.621(6)
C(43)-C(46A)	1.706(8)
C(47)-C(53)	1.509(3)
C(48)-C(53)	1.389(3)
C(48)-C(49)	1.397(3)
C(49)-C(50)	1.394(3)
C(49)-C(61)	1.523(3)
C(50)-C(51)	1.398(4)
C(51)-C(52)	1.382(4)
C(51)-C(57)	1.538(3)
C(52)-C(53)	1.395(3)
C(54)-C(55)	1.507(4)
C(55)-C(56)	1.531(5)
C(57)-C(59)	1.513(5)
C(57)-C(60)	1.534(4)
C(57)-C(58)	1.538(5)

C(1)-O(1)-C(7)	114.3(2)
C(20)-O(2)-C(26)	113.60(18)
C(34)-O(3)-C(40)	115.54(18)
C(48)-O(4)-C(54)	113.62(17)
O(1)-C(1)-C(6)	118.0(2)
O(1)-C(1)-C(2)	120.0(2)
C(6)-C(1)-C(2)	121.9(2)
C(3)-C(2)-C(1)	117.5(2)
C(3)-C(2)-C(19)	118.9(2)
C(1)-C(2)-C(19)	123.5(2)
C(2)-C(3)-C(4)	122.1(2)
C(5)-C(4)-C(3)	118.1(2)
C(5)-C(4)-C(10)	118.3(3)
C(3)-C(4)-C(10)	123.3(3)
C(5)-C(4)-C(10A)	122.3(3)

C(3)-C(4)-C(10A)	119.5(3)
C(10)-C(4)-C(10A)	8.4(4)
C(6)-C(5)-C(4)	121.9(2)
C(5)-C(6)-C(1)	118.3(2)
C(5)-C(6)-C(61)	119.6(2)
C(1)-C(6)-C(61)	122.0(2)
O(1)-C(7)-C(8)	107.1(4)
C(9)-C(8)-C(7)	114.9(6)
C(11)-O(5)-C(16)	118.7(5)
C(14)-O(6)-C(18)	116.0(4)
C(15)-C(10)-C(4)	122.5(5)
C(15)-C(10)-C(11)	116.6(6)
C(4)-C(10)-C(11)	120.9(6)
O(5)-C(11)-C(12)	121.7(7)
O(5)-C(11)-C(10)	118.6(7)
C(12)-C(11)-C(10)	119.6(7)
C(13)-C(12)-C(11)	120.1(6)
C(12)-C(13)-C(14)	119.6(4)
C(12)-C(13)-C(17)	119.1(5)
C(14)-C(13)-C(17)	121.2(4)
O(6)-C(14)-C(15)	124.7(5)
O(6)-C(14)-C(13)	114.4(4)
C(15)-C(14)-C(13)	120.8(4)
C(10)-C(15)-C(14)	123.1(6)
C(16A)-O(5A)-C(11A)	117.3(4)
C(14A)-O(6A)-C(18A)	118.1(5)
C(15A)-C(10A)-C(11A)	118.6(6)
C(15A)-C(10A)-C(4)	123.2(6)
C(11A)-C(10A)-C(4)	118.0(5)
C(12A)-C(11A)-O(5A)	123.1(5)
C(12A)-C(11A)-C(10A)	118.1(5)
O(5A)-C(11A)-C(10A)	118.8(5)
C(13A)-C(12A)-C(11A)	120.9(5)
C(12A)-C(13A)-C(14A)	117.5(5)
C(12A)-C(13A)-C(17A)	121.0(5)
C(14A)-C(13A)-C(17A)	121.5(5)

C(15A)-C(14A)-O(6A)	123.5(6)
C(15A)-C(14A)-C(13A)	121.2(6)
O(6A)-C(14A)-C(13A)	115.3(5)
C(10A)-C(15A)-C(14A)	123.5(7)
C(25)-C(19)-C(2)	118.1(2)
O(2)-C(20)-C(21)	121.0(2)
O(2)-C(20)-C(25)	118.1(2)
C(21)-C(20)-C(25)	120.8(2)
C(22)-C(21)-C(20)	118.3(2)
C(22)-C(21)-C(33)	118.2(2)
C(20)-C(21)-C(33)	123.5(2)
C(23)-C(22)-C(21)	122.9(2)
C(22)-C(23)-C(24)	116.7(2)
C(22)-C(23)-C(29)	121.6(2)
C(24)-C(23)-C(29)	121.7(2)
C(25)-C(24)-C(23)	122.4(2)
C(24)-C(25)-C(20)	118.7(2)
C(24)-C(25)-C(19)	121.1(2)
C(20)-C(25)-C(19)	120.2(2)
O(2)-C(26)-C(27)	108.4(2)
C(26)-C(27)-C(28)	112.6(3)
C(30A)-C(29)-C(30)	135.2(5)
C(30A)-C(29)-C(23)	112.1(5)
C(30)-C(29)-C(23)	112.6(4)
C(30A)-C(29)-C(32A)	112.7(6)
C(30)-C(29)-C(32A)	50.3(5)
C(23)-C(29)-C(32A)	112.5(4)
C(30A)-C(29)-C(32)	45.4(4)
C(30)-C(29)-C(32)	111.6(4)
C(23)-C(29)-C(32)	112.9(3)
C(32A)-C(29)-C(32)	134.6(5)
C(30A)-C(29)-C(31A)	111.1(6)
C(30)-C(29)-C(31A)	51.8(4)
C(23)-C(29)-C(31A)	106.9(4)
C(32A)-C(29)-C(31A)	100.9(5)
C(32)-C(29)-C(31A)	67.8(4)

C(30A)-C(29)-C(31)	58.9(5)
C(30)-C(29)-C(31)	110.2(4)
C(23)-C(29)-C(31)	106.2(3)
C(32A)-C(29)-C(31)	62.0(4)
C(32)-C(29)-C(31)	102.7(3)
C(31A)-C(29)-C(31)	146.7(4)
C(39)-C(33)-C(21)	118.03(19)
O(3)-C(34)-C(39)	118.24(19)
O(3)-C(34)-C(35)	120.89(19)
C(39)-C(34)-C(35)	120.8(2)
C(36)-C(35)-C(34)	118.3(2)
C(36)-C(35)-C(47)	118.1(2)
C(34)-C(35)-C(47)	123.6(2)
C(37)-C(36)-C(35)	123.1(2)
C(36)-C(37)-C(38)	116.7(2)
C(36)-C(37)-C(43)	121.0(3)
C(38)-C(37)-C(43)	122.3(3)
C(39)-C(38)-C(37)	122.3(2)
C(34)-C(39)-C(38)	118.8(2)
C(34)-C(39)-C(33)	120.5(2)
C(38)-C(39)-C(33)	120.6(2)
O(3)-C(40)-C(41)	107.3(2)
C(40)-C(41)-C(42)	112.3(3)
C(44A)-C(43)-C(44)	133.1(5)
C(44A)-C(43)-C(45)	41.9(4)
C(44)-C(43)-C(45)	117.0(4)
C(44A)-C(43)-C(37)	115.3(4)
C(44)-C(43)-C(37)	111.1(3)
C(45)-C(43)-C(37)	107.3(3)
C(44A)-C(43)-C(45A)	115.0(5)
C(44)-C(43)-C(45A)	48.8(4)
C(45)-C(43)-C(45A)	140.9(4)
C(37)-C(43)-C(45A)	111.7(4)
C(44A)-C(43)-C(46)	61.7(4)
C(44)-C(43)-C(46)	108.6(4)
C(45)-C(43)-C(46)	103.3(4)

C(37)-C(43)-C(46)	109.1(3)
C(45A)-C(43)-C(46)	62.3(4)
C(44A)-C(43)-C(46A)	107.2(5)
C(44)-C(43)-C(46A)	48.1(4)
C(45)-C(43)-C(46A)	72.5(4)
C(37)-C(43)-C(46A)	110.6(3)
C(45A)-C(43)-C(46A)	94.9(4)
C(46)-C(43)-C(46A)	139.5(4)
C(53)-C(47)-C(35)	119.96(19)
O(4)-C(48)-C(53)	118.0(2)
O(4)-C(48)-C(49)	120.61(19)
C(53)-C(48)-C(49)	121.3(2)
C(50)-C(49)-C(48)	117.6(2)
C(50)-C(49)-C(61)	119.0(2)
C(48)-C(49)-C(61)	123.3(2)
C(49)-C(50)-C(51)	122.8(2)
C(52)-C(51)-C(50)	117.3(2)
C(52)-C(51)-C(57)	122.4(2)
C(50)-C(51)-C(57)	120.3(3)
C(51)-C(52)-C(53)	122.1(2)
C(48)-C(53)-C(52)	118.8(2)
C(48)-C(53)-C(47)	121.3(2)
C(52)-C(53)-C(47)	119.8(2)
O(4)-C(54)-C(55)	107.5(2)
C(54)-C(55)-C(56)	111.3(3)
C(59)-C(57)-C(60)	109.6(3)
C(59)-C(57)-C(51)	109.2(2)
C(60)-C(57)-C(51)	109.5(2)
C(59)-C(57)-C(58)	108.4(3)
C(60)-C(57)-C(58)	108.3(3)
C(51)-C(57)-C(58)	111.8(3)
C(6)-C(61)-C(49)	117.67(19)

Symmetry transformations used to generate equivalent atoms:

Table S4. Anisotropic displacement parameters ($Å^2 x 10^3$)for raj_s. The anisotropic displacement factor exponent takes the form: $-2\pi^2[h^2a^{*2}U^{11} + ... + 2 h k a^* b^* U^{12}]$

	U^{11}	U^{22}	U^{33}	U^{23}	U^{13}	U^{12}
O(1)	55(1)	50(1)	56(1)	-3(1)	-26(1)	13(1)
O(2)	56(1)	32(1)	31(1)	4(1)	-5(1)	-5(1)
O(3)	49(1)	29(1)	38(1)	-4(1)	11(1)	-4(1)
O(4)	33(1)	30(1)	49(1)	-3(1)	-13(1)	-4(1)
C(1)	31(1)	42(1)	48(1)	-1(1)	-10(1)	12(1)
C(2)	25(1)	39(1)	51(1)	7(1)	-2(1)	5(1)
C(3)	23(1)	35(1)	52(1)	5(1)	-5(1)	1(1)
C(4)	24(1)	34(1)	44(1)	2(1)	-2(1)	5(1)
C(5)	29(1)	30(1)	52(1)	-2(1)	-3(1)	7(1)
C(6)	28(1)	34(1)	56(1)	-11(1)	-7(1)	9(1)
C(7)	106(3)	66(2)	57(2)	4(2)	-29(2)	16(2)
C(8)	184(5)	74(2)	65(2)	1(2)	-57(3)	18(3)
C(9)	335(11)	84(3)	71(3)	-7(2)	-65(5)	47(5)
C(19)	29(1)	51(1)	53(2)	16(1)	-5(1)	2(1)
C(20)	37(1)	26(1)	33(1)	2(1)	-4(1)	-7(1)
C(21)	37(1)	21(1)	38(1)	0(1)	3(1)	-3(1)
C(22)	31(1)	28(1)	40(1)	2(1)	-3(1)	0(1)
C(23)	36(1)	38(1)	34(1)	2(1)	-6(1)	1(1)
C(24)	33(1)	48(1)	36(1)	4(1)	3(1)	7(1)
C(25)	29(1)	37(1)	39(1)	6(1)	-4(1)	0(1)
C(26)	82(2)	48(2)	43(1)	-2(1)	-21(1)	-7(2)
C(27)	112(3)	61(2)	39(2)	-5(1)	-20(2)	15(2)
C(28)	198(6)	92(3)	64(2)	-17(2)	-69(3)	10(3)
C(29)	57(2)	57(2)	34(1)	6(1)	-4(1)	14(1)
C(33)	53(1)	22(1)	55(2)	-1(1)	18(1)	-1(1)
C(34)	35(1)	27(1)	35(1)	-1(1)	7(1)	2(1)
C(35)	29(1)	30(1)	43(1)	6(1)	5(1)	3(1)
C(36)	39(1)	49(1)	34(1)	7(1)	6(1)	5(1)
C(37)	46(1)	55(2)	36(1)	-7(1)	3(1)	10(1)
C(38)	48(1)	38(1)	46(1)	-11(1)	7(1)	2(1)
C(39)	42(1)	27(1)	41(1)	-2(1)	11(1)	1(1)

C(40)	68(2)	34(1)	35(1)	2(1)	-4(1)	9(1)
C(41)	97(2)	50(2)	40(1)	-5(1)	3(2)	-8(2)
C(42)	139(4)	64(2)	45(2)	-5(2)	-18(2)	10(2)
C(43)	67(2)	79(2)	36(1)	-11(1)	3(1)	13(2)
C(47)	30(1)	32(1)	53(1)	11(1)	2(1)	-3(1)
C(48)	31(1)	21(1)	48(1)	1(1)	-12(1)	-3(1)
C(49)	35(1)	23(1)	56(1)	-4(1)	-15(1)	2(1)
C(50)	46(1)	26(1)	54(2)	-6(1)	-15(1)	-1(1)
C(51)	40(1)	29(1)	57(2)	5(1)	-20(1)	-10(1)
C(52)	29(1)	29(1)	58(2)	9(1)	-11(1)	-7(1)
C(53)	29(1)	24(1)	50(1)	8(1)	-5(1)	-6(1)
C(54)	38(1)	48(1)	48(1)	5(1)	-8(1)	-2(1)
C(55)	43(1)	74(2)	48(2)	-6(1)	-9(1)	-3(1)
C(56)	78(2)	122(3)	59(2)	22(2)	-23(2)	-18(2)
C(57)	56(2)	43(1)	65(2)	-2(1)	-30(1)	-13(1)
C(58)	55(2)	83(2)	109(3)	-11(2)	-44(2)	-12(2)
C(59)	85(3)	74(2)	72(2)	-14(2)	-34(2)	-10(2)
C(60)	107(3)	45(2)	129(3)	-10(2)	-65(3)	-23(2)
C(61)	40(1)	33(1)	67(2)	-14(1)	-17(1)	10(1)

Table S5. Hydrogen coordinates (x 10^4) and isotropic displacement parameters ($Å^2$x 10^3) for raj_s.

	x	y	z	U(eq)
H(3A)	-558	4043	1079	44
H(5A)	1109	5871	1053	44
H(7A)	1173	4361	1935	91
H(7B)	1578	5132	1890	91
H(8A)	3292	4117	2076	129
H(8B)	3663	4890	2039	129
H(9A)	3356	4659	2453	245
H(9B)	1945	4402	2394	245
H(9C)	2300	5179	2356	245
H(12A)	-1772	4861	250	45
H(15A)	-1422	6015	1026	26
H(16A)	654	3675	291	74
H(16B)	388	4406	175	74
H(16C)	-774	3914	239	74
H(17A)	-3974	6217	330	66
H(17B)	-3976	5472	213	66
H(17C)	-3001	6023	107	66
H(18A)	-3822	7171	967	65
H(18B)	-2353	6984	1002	65
H(18C)	-3428	6469	1095	65
H(12B)	-2970	6209	625	39
H(15B)	-172	4423	588	35
H(16D)	-2471	6840	1239	70
H(16E)	-3397	6377	1072	70
H(16F)	-2526	6930	936	70
H(17D)	-2868	5893	64	92
H(17E)	-4061	5758	246	92
H(17F)	-3429	5148	92	92
H(18D)	-579	3861	-22	109
H(18E)	-541	3743	279	109

H(18F)	340	4307	152	109
H(19A)	-64	3603	1643	54
H(19B)	-51	3235	1373	54
H(22A)	4349	2059	1780	40
H(24A)	1083	3015	1946	47
H(26A)	995	2393	1073	69
H(26B)	2377	2188	970	69
H(27A)	2248	3058	661	85
H(27B)	858	3246	761	85
H(28A)	709	2586	391	177
H(28B)	102	2186	626	177
H(28C)	1492	1995	526	177
H(30A)	1935	2916	2405	126
H(30B)	3261	3181	2294	126
H(30C)	3241	2650	2524	126
H(31A)	1198	1665	2070	90
H(31B)	1029	1969	2350	90
H(31C)	1918	1326	2306	90
H(32A)	3764	1316	2169	75
H(32B)	4172	1751	2412	75
H(32C)	4715	1938	2136	75
H(30D)	2259	1312	2120	116
H(30E)	3028	1399	2381	116
H(30F)	3769	1402	2114	116
H(31D)	4483	2878	2178	108
H(31E)	4597	2301	2390	108
H(31F)	3721	2946	2441	108
H(32D)	932	2306	2297	117
H(32E)	1684	2997	2340	117
H(32F)	1891	2381	2532	117
H(33A)	5332	2151	1395	52
H(33B)	4280	1867	1205	52
H(36A)	6231	3931	590	49
H(38A)	4706	2150	787	52
H(40A)	7281	3799	1479	54
H(40B)	6914	3028	1536	54

H(41A)	5700	3363	1890	74
H(41B)	5995	4138	1830	74
H(42A)	7303	3757	2162	124
H(42B)	8159	3956	1921	124
H(42C)	7866	3181	1981	124
H(44A)	3837	3116	224	103
H(44B)	4995	3209	30	103
H(44C)	4854	3703	269	103
H(45A)	7212	2296	354	110
H(45B)	7369	3097	338	110
H(45C)	6929	2681	92	110
H(46A)	4884	1830	499	96
H(46B)	5436	1780	215	96
H(46C)	4038	2070	263	96
H(44D)	6104	1894	404	88
H(44E)	7168	2430	325	88
H(44F)	6178	2162	117	88
H(45D)	3677	2348	333	81
H(45E)	4148	2640	65	81
H(45F)	3631	3141	280	81
H(46D)	6230	3827	275	73
H(46E)	5113	3632	81	73
H(46F)	6505	3325	43	73
H(47A)	7422	4443	1074	46
H(47B)	6505	4787	871	46
H(50A)	4651	6075	1739	51
H(52A)	7604	5080	1433	47
H(54A)	4661	5461	762	54
H(54B)	3351	5746	876	54
H(55A)	2324	4749	698	66
H(55B)	3624	4602	549	66
H(56A)	2202	5187	278	129
H(56B)	3420	5648	331	129
H(56C)	2119	5792	479	129
H(58A)	9045	5903	1929	123
H(58B)	8840	5881	1627	123

H(58C)	8595	5221	1795	123
H(59A)	7226	5849	2218	115
H(59B)	6735	5172	2086	115
H(59C)	5805	5806	2111	115
H(60A)	7603	6877	1958	140
H(60B)	6184	6836	1850	140
H(60C)	7361	6859	1657	140
H(61A)	2565	5903	1602	56
H(61B)	2714	6183	1319	56

Table S1. Crystal data and structure refinement for raj0f.

Identification code	raj0f	
Empirical formula	C66 H84 Cl2.86 O8	
Formula weight	1106.72	
Temperature	100(2) K	
Wavelength	1.54178 Å	
Crystal system	Monoclinic	
Space group	P 21/c	
Unit cell dimensions	a = 9.5632(9) Å	α= 90°.
	b = 19.7955(18) Å	β= 92.207(5)°.
	c = 32.047(3) Å	γ = 90°.
Volume	6062.3(10) Å^3	
Z	4	
Density (calculated)	1.213 Mg/m^3	
Absorption coefficient	1.733 mm^{-1}	
F(000)	2370	
Crystal size	0.38 x 0.21 x 0.06 mm^3	
Theta range for data collection	2.62 to 67.52°.	
Index ranges	-11<=h<=11, 0<=k<=23, 0<=l<=37	
Reflections collected	50111	
Independent reflections	10526 [R(int) = 0.0767]	
Completeness to theta = 67.52°	96.2 %	
Absorption correction	Semi-empirical from equivalents	
Max. and min. transmission	0.9031 and 0.5588	
Refinement method	Full-matrix least-squares on F^2	
Data / restraints / parameters	10526 / 6 / 728	
Goodness-of-fit on F^2	1.032	
Final R indices [I>2sigma(I)]	R1 = 0.1369, wR2 = 0.3367	
R indices (all data)	R1 = 0.1696, wR2 = 0.3537	
Extinction coefficient	0.00137(17)	
Largest diff. peak and hole	0.542 and -0.458 e.Å^{-3}	

Table S2. Atomic coordinates (x 10^4) and equivalent isotropic displacement parameters ($Å^2$x 10^3) for raj0f. U(eq) is defined as one third of the trace of the orthogonalized U^{ij} tensor.

	x	y	z	U(eq)
O(1)	6598(5)	1529(2)	-3531(2)	61(1)
O(2)	3931(4)	2354(2)	-2760(1)	41(1)
O(3)	2693(4)	769(2)	-3403(1)	45(1)
O(4)	5450(4)	299(2)	-2603(1)	47(1)
O(5)	9561(4)	3308(2)	-2131(1)	54(1)
O(6)	6957(7)	2018(3)	-836(2)	95(2)
O(7)	-248(7)	-615(3)	-1933(2)	90(2)
O(8)	566(6)	1436(3)	-778(2)	80(2)
C(10)	8015(7)	575(3)	-2966(2)	57(2)
C(11)	7032(6)	1751(3)	-3138(2)	54(2)
C(12)	7622(6)	1307(3)	-2853(2)	54(2)
C(13)	7948(6)	1534(3)	-2452(2)	54(2)
C(14)	7789(6)	2203(3)	-2337(2)	51(2)
C(15)	7234(6)	2648(3)	-2644(2)	53(2)
C(16)	6782(6)	2430(3)	-3035(2)	46(2)
C(17)	7547(10)	1611(4)	-3844(3)	80(2)
C(18)	6768(15)	1383(6)	-4231(3)	121(4)
C(19)	7517(16)	1447(8)	-4590(5)	97(4)
C(18A)	6768(15)	1383(6)	-4231(3)	121(4)
C(19A)	6250(30)	1778(14)	-4503(9)	72(7)
C(20)	6000(6)	2898(3)	-3330(2)	51(2)
C(21)	3572(6)	2420(3)	-3178(2)	39(1)
C(22)	4502(7)	2697(3)	-3454(2)	48(2)
C(23)	4050(7)	2794(3)	-3867(2)	49(2)
C(24)	2701(7)	2621(3)	-4017(2)	48(2)
C(25)	1826(7)	2304(3)	-3734(2)	51(2)
C(26)	2247(6)	2204(3)	-3316(2)	42(1)
C(27)	3710(7)	2969(3)	-2538(2)	51(2)
C(28)	4130(8)	2876(4)	-2078(2)	59(2)
C(29)	3334(9)	2337(4)	-1880(2)	75(2)
C(30)	1235(6)	1895(3)	-3019(2)	43(1)

C(31)	2251(6)	691(3)	-3001(2)	44(1)
C(32)	1600(6)	1231(3)	-2803(2)	37(1)
C(33)	1189(6)	1135(3)	-2394(2)	44(1)
C(34)	1305(6)	524(3)	-2190(2)	48(2)
C(35)	1981(7)	0(3)	-2397(2)	48(2)
C(36)	2462(6)	73(3)	-2792(2)	43(1)
C(37)	1728(7)	535(4)	-3719(2)	55(2)
C(38)	2441(8)	546(4)	-4128(2)	64(2)
C(39)	1517(12)	283(5)	-4479(3)	97(3)
C(40)	3205(7)	-513(3)	-3002(2)	53(2)
C(41)	5641(6)	26(3)	-2991(2)	43(1)
C(42)	4590(6)	-360(3)	-3191(2)	45(1)
C(43)	4846(7)	-638(3)	-3578(2)	48(2)
C(44)	6109(7)	-545(3)	-3776(2)	52(2)
C(45)	7124(7)	-142(3)	-3571(2)	52(2)
C(46)	6888(7)	153(3)	-3186(2)	48(2)
C(47)	5705(9)	-161(4)	-2259(2)	71(2)
C(48)	5583(13)	207(4)	-1870(3)	94(3)
C(49)	5249(18)	-226(9)	-1502(5)	96(5)
C(48A)	5583(13)	207(4)	-1870(3)	94(3)
C(49A)	4590(30)	523(14)	-1738(9)	101(8)
C(51)	8092(7)	2438(3)	-1900(2)	53(2)
C(52)	8920(7)	3002(3)	-1804(2)	51(2)
C(53)	9072(7)	3211(4)	-1394(2)	60(2)
C(54)	8414(9)	2887(4)	-1069(2)	67(2)
C(55)	7593(8)	2336(5)	-1168(3)	75(2)
C(56)	7445(8)	2106(4)	-1582(2)	64(2)
C(57)	10487(7)	3861(3)	-2032(2)	52(2)
C(58)	8556(11)	3129(6)	-616(3)	100(3)
C(59)	6070(11)	1452(6)	-923(4)	117(4)
C(60)	2200(8)	2760(3)	-4469(2)	64(2)
C(61)	816(16)	2459(9)	-4563(5)	77(4)
C(62)	2261(17)	3526(7)	-4539(5)	73(4)
C(63)	3360(14)	2442(7)	-4748(4)	60(4)
C(60A)	2200(8)	2760(3)	-4469(2)	64(2)
C(61A)	3160(20)	3156(12)	-4732(7)	126(8)

C(62A)	1754(17)	2100(7)	-4705(5)	69(4)
C(63A)	717(19)	3144(11)	-4476(7)	109(7)
C(71)	756(7)	453(3)	-1758(2)	51(2)
C(72)	-42(10)	-109(4)	-1647(2)	74(2)
C(73)	-640(10)	-122(5)	-1253(3)	90(3)
C(74)	-446(10)	389(5)	-963(3)	81(2)
C(75)	375(9)	944(4)	-1084(2)	68(2)
C(76)	943(7)	968(4)	-1474(2)	57(2)
C(77)	-546(13)	-1276(4)	-1778(3)	108(4)
C(78)	-1031(12)	372(6)	-524(3)	117(4)
C(79)	1484(10)	1966(4)	-881(2)	77(2)
C(80)	6343(8)	-893(4)	-4199(2)	63(2)
C(81)	7839(9)	-752(6)	-4347(3)	98(3)
C(82)	6175(13)	-1646(4)	-4153(3)	100(3)
C(83)	5349(10)	-613(5)	-4533(2)	82(3)
Cl(1X)	3130(8)	351(7)	-382(2)	104(4)
Cl(2X)	2080(20)	-966(8)	-515(4)	125(2)
Cl(3X)	4732(12)	-514(5)	-863(3)	125(2)
Cl(4X)	3124(13)	-1481(6)	-226(4)	125(2)
Cl(5X)	2270(20)	-1140(13)	-929(7)	125(2)
Cl(6X)	4251(19)	-214(8)	-251(5)	125(2)
Cl(7X)	2030(30)	-557(12)	-783(7)	125(2)
Cl(8X)	2770(30)	-729(14)	-1045(8)	125(2)
Cl(9X)	3210(20)	-40(18)	-323(6)	125(2)
Cl(10)	2920(30)	-279(12)	-705(7)	125(2)
Cl(11)	2760(50)	-1086(14)	-462(8)	125(2)
Cl(12)	2340(60)	-1450(20)	-44(16)	125(2)
Cl(13)	3870(50)	-480(19)	-1102(12)	125(2)
Cl(14)	3630(70)	-900(30)	-575(17)	125(2)

Table S3. Bond lengths [Å] and angles [°] for raj0f.

O(1)-C(11)	1.382(8)
O(1)-C(17)	1.389(10)
O(2)-C(21)	1.378(7)
O(2)-C(27)	1.429(7)
O(3)-C(31)	1.381(7)
O(3)-C(37)	1.422(8)
O(4)-C(41)	1.375(7)
O(4)-C(47)	1.442(8)
O(5)-C(52)	1.375(8)
O(5)-C(57)	1.436(7)
O(6)-C(55)	1.396(9)
O(6)-C(59)	1.426(12)
O(7)-C(72)	1.366(9)
O(7)-C(77)	1.430(9)
O(8)-C(75)	1.390(9)
O(8)-C(79)	1.414(10)
C(10)-C(46)	1.516(9)
C(10)-C(12)	1.543(9)
C(10)-H(10A)	0.9900
C(10)-H(10B)	0.9900
C(11)-C(12)	1.373(9)
C(11)-C(16)	1.406(9)
C(12)-C(13)	1.387(10)
C(13)-C(14)	1.384(9)
C(13)-H(13)	0.9500
C(14)-C(15)	1.408(9)
C(14)-C(51)	1.496(10)
C(15)-C(16)	1.379(9)
C(15)-H(15)	0.9500
C(16)-C(20)	1.503(9)
C(17)-C(18)	1.493(13)
C(17)-H(17A)	0.9900
C(17)-H(17B)	0.9900
C(18)-C(19)	1.383(17)

C(18)-H(18A)	0.9900
C(18)-H(18B)	0.9900
C(19)-H(19A)	0.9800
C(19)-H(19B)	0.9800
C(19)-H(19C)	0.9800
C(19A)-H(19D)	0.9800
C(19A)-H(19E)	0.9800
C(19A)-H(19F)	0.9800
C(20)-C(22)	1.524(9)
C(20)-H(20A)	0.9900
C(20)-H(20B)	0.9900
C(21)-C(22)	1.390(8)
C(21)-C(26)	1.392(8)
C(22)-C(23)	1.391(9)
C(23)-C(24)	1.401(9)
C(23)-H(23)	0.9500
C(24)-C(25)	1.405(9)
C(24)-C(60)	1.534(9)
C(25)-C(26)	1.400(9)
C(25)-H(25)	0.9500
C(26)-C(30)	1.510(8)
C(27)-C(28)	1.524(9)
C(27)-H(27A)	0.9900
C(27)-H(27B)	0.9900
C(28)-C(29)	1.469(10)
C(28)-H(28A)	0.9900
C(28)-H(28B)	0.9900
C(29)-H(29A)	0.9800
C(29)-H(29B)	0.9800
C(29)-H(29C)	0.9800
C(30)-C(32)	1.520(8)
C(30)-H(30A)	0.9900
C(30)-H(30B)	0.9900
C(31)-C(32)	1.401(8)
C(31)-C(36)	1.406(8)
C(32)-C(33)	1.396(8)

C(33)-C(34)	1.378(9)
C(33)-H(33)	0.9500
C(34)-C(35)	1.402(9)
C(34)-C(71)	1.505(9)
C(35)-C(36)	1.372(9)
C(35)-H(35)	0.9500
C(36)-C(40)	1.529(8)
C(37)-C(38)	1.500(9)
C(37)-H(37A)	0.9900
C(37)-H(37B)	0.9900
C(38)-C(39)	1.496(12)
C(38)-H(38A)	0.9900
C(38)-H(38B)	0.9900
C(39)-H(39A)	0.9800
C(39)-H(39B)	0.9800
C(39)-H(39C)	0.9800
C(40)-C(42)	1.509(9)
C(40)-H(40A)	0.9900
C(40)-H(40B)	0.9900
C(41)-C(46)	1.389(9)
C(41)-C(42)	1.399(8)
C(42)-C(43)	1.387(9)
C(43)-C(44)	1.398(9)
C(43)-H(43)	0.9500
C(44)-C(45)	1.400(9)
C(44)-C(80)	1.545(10)
C(45)-C(46)	1.392(9)
C(45)-H(45)	0.9500
C(47)-C(48)	1.454(11)
C(47)-H(47A)	0.9900
C(47)-H(47B)	0.9900
C(48)-C(49)	1.501(18)
C(48)-H(48A)	0.9900
C(48)-H(48B)	0.9900
C(49)-H(49A)	0.9800
C(49)-H(49B)	0.9800

C(49)-H(49C)	0.9800
C(49A)-H(49D)	0.9800
C(49A)-H(49E)	0.9800
C(49A)-H(49F)	0.9800
C(51)-C(56)	1.379(10)
C(51)-C(52)	1.395(9)
C(52)-C(53)	1.380(10)
C(53)-C(54)	1.393(10)
C(53)-H(53)	0.9500
C(54)-C(55)	1.375(11)
C(54)-C(58)	1.530(12)
C(55)-C(56)	1.403(11)
C(56)-H(56)	0.9500
C(57)-H(57A)	0.9800
C(57)-H(57B)	0.9800
C(57)-H(57C)	0.9800
C(58)-H(58A)	0.9800
C(58)-H(58B)	0.9800
C(58)-H(58C)	0.9800
C(59)-H(59A)	0.9800
C(59)-H(59B)	0.9800
C(59)-H(59C)	0.9800
C(60)-C(61)	1.472(15)
C(60)-C(62)	1.534(14)
C(60)-C(63)	1.582(13)
C(61)-H(61A)	0.9800
C(61)-H(61B)	0.9800
C(61)-H(61C)	0.9800
C(62)-H(62A)	0.9800
C(62)-H(62B)	0.9800
C(62)-H(62C)	0.9800
C(63)-H(63A)	0.9800
C(63)-H(63B)	0.9800
C(63)-H(63C)	0.9800
C(61A)-H(61D)	0.9800
C(61A)-H(61E)	0.9800

C(61A)-H(61F)	0.9800
C(62A)-H(62D)	0.9800
C(62A)-H(62E)	0.9800
C(62A)-H(62F)	0.9800
C(63A)-H(63D)	0.9800
C(63A)-H(63E)	0.9800
C(63A)-H(63F)	0.9800
C(71)-C(76)	1.374(9)
C(71)-C(72)	1.403(10)
C(72)-C(73)	1.405(11)
C(73)-C(74)	1.381(12)
C(73)-H(73)	0.9500
C(74)-C(75)	1.413(11)
C(74)-C(78)	1.535(12)
C(75)-C(76)	1.383(10)
C(76)-H(76)	0.9500
C(77)-H(77A)	0.9800
C(77)-H(77B)	0.9800
C(77)-H(77C)	0.9800
C(78)-H(78A)	0.9800
C(78)-H(78B)	0.9800
C(78)-H(78C)	0.9800
C(79)-H(79A)	0.9800
C(79)-H(79B)	0.9800
C(79)-H(79C)	0.9800
C(80)-C(82)	1.507(11)
C(80)-C(83)	1.511(10)
C(80)-C(81)	1.550(12)
C(81)-H(81A)	0.9800
C(81)-H(81B)	0.9800
C(81)-H(81C)	0.9800
C(82)-H(82A)	0.9800
C(82)-H(82B)	0.9800
C(82)-H(82C)	0.9800
C(83)-H(83A)	0.9800
C(83)-H(83B)	0.9800

C(83)-H(83C)	0.9800
Cl(1X)-Cl(9X)	0.80(3)
Cl(1X)-Cl(6X)	1.59(2)
Cl(1X)-Cl(10)	1.63(3)
Cl(1X)-Cl(7X)	2.43(3)
Cl(2X)-Cl(11)	0.70(3)
Cl(2X)-Cl(7X)	1.18(2)
Cl(2X)-Cl(5X)	1.39(2)
Cl(2X)-Cl(14)	1.51(7)
Cl(2X)-Cl(4X)	1.68(2)
Cl(2X)-Cl(10)	1.71(3)
Cl(2X)-Cl(12)	1.79(5)
Cl(2X)-Cl(8X)	1.90(3)
Cl(2X)-Cl(9X)	2.20(3)
Cl(3X)-Cl(13)	1.11(4)
Cl(3X)-Cl(14)	1.62(7)
Cl(3X)-Cl(10)	1.88(3)
Cl(3X)-Cl(8X)	1.99(3)
Cl(3X)-Cl(6X)	2.119(19)
Cl(4X)-Cl(12)	0.97(5)
Cl(4X)-Cl(11)	1.13(3)
Cl(4X)-Cl(14)	1.68(6)
Cl(4X)-Cl(5X)	2.46(3)
Cl(5X)-Cl(8X)	1.02(3)
Cl(5X)-Cl(7X)	1.27(3)
Cl(5X)-Cl(11)	1.55(4)
Cl(5X)-Cl(14)	1.76(6)
Cl(5X)-Cl(10)	1.94(4)
Cl(5X)-Cl(13)	2.10(5)
Cl(6X)-Cl(9X)	1.07(3)
Cl(6X)-Cl(14)	1.80(6)
Cl(6X)-Cl(10)	1.90(3)
Cl(6X)-Cl(6X)#1	2.27(3)
Cl(6X)-Cl(11)	2.33(3)
Cl(7X)-Cl(10)	1.04(3)
Cl(7X)-Cl(8X)	1.17(3)

Cl(7X)-Cl(11)	1.61(5)
Cl(7X)-Cl(14)	1.78(6)
Cl(7X)-Cl(13)	2.07(5)
Cl(7X)-Cl(9X)	2.09(3)
Cl(8X)-Cl(13)	1.18(4)
Cl(8X)-Cl(10)	1.41(3)
Cl(8X)-Cl(14)	1.72(6)
Cl(8X)-Cl(11)	2.00(4)
Cl(9X)-Cl(10)	1.33(3)
Cl(9X)-Cl(14)	1.94(6)
Cl(9X)-Cl(11)	2.16(4)
Cl(10)-Cl(14)	1.46(6)
Cl(10)-Cl(13)	1.63(4)
Cl(10)-Cl(11)	1.79(4)
Cl(11)-Cl(14)	0.99(6)
Cl(11)-Cl(12)	1.58(6)
Cl(12)-Cl(14)	2.39(9)
Cl(13)-Cl(14)	1.91(7)

C(11)-O(1)-C(17)	116.1(6)
C(21)-O(2)-C(27)	111.5(4)
C(31)-O(3)-C(37)	114.5(4)
C(41)-O(4)-C(47)	114.7(5)
C(52)-O(5)-C(57)	117.2(5)
C(55)-O(6)-C(59)	118.6(8)
C(72)-O(7)-C(77)	117.7(6)
C(75)-O(8)-C(79)	114.9(6)
C(46)-C(10)-C(12)	116.9(5)
C(46)-C(10)-H(10A)	108.1
C(12)-C(10)-H(10A)	108.1
C(46)-C(10)-H(10B)	108.1
C(12)-C(10)-H(10B)	108.1
H(10A)-C(10)-H(10B)	107.3
C(12)-C(11)-O(1)	120.1(6)
C(12)-C(11)-C(16)	121.7(7)
O(1)-C(11)-C(16)	118.1(6)

C(11)-C(12)-C(13)	118.8(6)
C(11)-C(12)-C(10)	122.9(7)
C(13)-C(12)-C(10)	118.2(6)
C(14)-C(13)-C(12)	122.1(7)
C(14)-C(13)-H(13)	118.9
C(12)-C(13)-H(13)	118.9
C(13)-C(14)-C(15)	117.2(6)
C(13)-C(14)-C(51)	121.8(6)
C(15)-C(14)-C(51)	120.9(6)
C(16)-C(15)-C(14)	122.2(6)
C(16)-C(15)-H(15)	118.9
C(14)-C(15)-H(15)	118.9
C(15)-C(16)-C(11)	117.6(6)
C(15)-C(16)-C(20)	120.6(6)
C(11)-C(16)-C(20)	121.7(6)
O(1)-C(17)-C(18)	104.3(8)
O(1)-C(17)-H(17A)	110.9
C(18)-C(17)-H(17A)	110.9
O(1)-C(17)-H(17B)	110.9
C(18)-C(17)-H(17B)	110.9
H(17A)-C(17)-H(17B)	108.9
C(19)-C(18)-C(17)	114.0(12)
C(19)-C(18)-H(18A)	108.8
C(17)-C(18)-H(18A)	108.8
C(19)-C(18)-H(18B)	108.8
C(17)-C(18)-H(18B)	108.8
H(18A)-C(18)-H(18B)	107.6
C(18)-C(19)-H(19A)	109.5
C(18)-C(19)-H(19B)	109.5
H(19A)-C(19)-H(19B)	109.5
C(18)-C(19)-H(19C)	109.5
H(19A)-C(19)-H(19C)	109.5
H(19B)-C(19)-H(19C)	109.5
H(19D)-C(19A)-H(19E)	109.5
H(19D)-C(19A)-H(19F)	109.5
H(19E)-C(19A)-H(19F)	109.5

C(16)-C(20)-C(22)	116.4(5)
C(16)-C(20)-H(20A)	108.2
C(22)-C(20)-H(20A)	108.2
C(16)-C(20)-H(20B)	108.2
C(22)-C(20)-H(20B)	108.2
H(20A)-C(20)-H(20B)	107.4
O(2)-C(21)-C(22)	120.9(5)
O(2)-C(21)-C(26)	117.9(5)
C(22)-C(21)-C(26)	121.2(6)
C(21)-C(22)-C(23)	118.4(6)
C(21)-C(22)-C(20)	123.7(6)
C(23)-C(22)-C(20)	117.8(6)
C(22)-C(23)-C(24)	122.8(6)
C(22)-C(23)-H(23)	118.6
C(24)-C(23)-H(23)	118.6
C(23)-C(24)-C(25)	116.8(6)
C(23)-C(24)-C(60)	122.0(6)
C(25)-C(24)-C(60)	121.1(6)
C(26)-C(25)-C(24)	121.7(6)
C(26)-C(25)-H(25)	119.1
C(24)-C(25)-H(25)	119.1
C(21)-C(26)-C(25)	118.9(6)
C(21)-C(26)-C(30)	121.5(5)
C(25)-C(26)-C(30)	119.5(5)
O(2)-C(27)-C(28)	109.8(5)
O(2)-C(27)-H(27A)	109.7
C(28)-C(27)-H(27A)	109.7
O(2)-C(27)-H(27B)	109.7
C(28)-C(27)-H(27B)	109.7
H(27A)-C(27)-H(27B)	108.2
C(29)-C(28)-C(27)	112.7(6)
C(29)-C(28)-H(28A)	109.1
C(27)-C(28)-H(28A)	109.1
C(29)-C(28)-H(28B)	109.1
C(27)-C(28)-H(28B)	109.1
H(28A)-C(28)-H(28B)	107.8

C(28)-C(29)-H(29A)	109.5
C(28)-C(29)-H(29B)	109.5
H(29A)-C(29)-H(29B)	109.5
C(28)-C(29)-H(29C)	109.5
H(29A)-C(29)-H(29C)	109.5
H(29B)-C(29)-H(29C)	109.5
C(26)-C(30)-C(32)	119.7(5)
C(26)-C(30)-H(30A)	107.4
C(32)-C(30)-H(30A)	107.4
C(26)-C(30)-H(30B)	107.4
C(32)-C(30)-H(30B)	107.4
H(30A)-C(30)-H(30B)	106.9
O(3)-C(31)-C(32)	119.7(5)
O(3)-C(31)-C(36)	119.8(5)
C(32)-C(31)-C(36)	120.5(6)
C(33)-C(32)-C(31)	117.8(5)
C(33)-C(32)-C(30)	118.6(5)
C(31)-C(32)-C(30)	123.5(5)
C(34)-C(33)-C(32)	123.1(6)
C(34)-C(33)-H(33)	118.5
C(32)-C(33)-H(33)	118.5
C(33)-C(34)-C(35)	117.0(6)
C(33)-C(34)-C(71)	119.6(6)
C(35)-C(34)-C(71)	123.3(6)
C(36)-C(35)-C(34)	122.5(6)
C(36)-C(35)-H(35)	118.8
C(34)-C(35)-H(35)	118.8
C(35)-C(36)-C(31)	118.9(6)
C(35)-C(36)-C(40)	120.3(5)
C(31)-C(36)-C(40)	120.8(6)
O(3)-C(37)-C(38)	108.2(6)
O(3)-C(37)-H(37A)	110.0
C(38)-C(37)-H(37A)	110.0
O(3)-C(37)-H(37B)	110.0
C(38)-C(37)-H(37B)	110.0
H(37A)-C(37)-H(37B)	108.4

C(39)-C(38)-C(37)	112.1(7)
C(39)-C(38)-H(38A)	109.2
C(37)-C(38)-H(38A)	109.2
C(39)-C(38)-H(38B)	109.2
C(37)-C(38)-H(38B)	109.2
H(38A)-C(38)-H(38B)	107.9
C(38)-C(39)-H(39A)	109.5
C(38)-C(39)-H(39B)	109.5
H(39A)-C(39)-H(39B)	109.5
C(38)-C(39)-H(39C)	109.5
H(39A)-C(39)-H(39C)	109.5
H(39B)-C(39)-H(39C)	109.5
C(42)-C(40)-C(36)	117.1(5)
C(42)-C(40)-H(40A)	108.0
C(36)-C(40)-H(40A)	108.0
C(42)-C(40)-H(40B)	108.0
C(36)-C(40)-H(40B)	108.0
H(40A)-C(40)-H(40B)	107.3
O(4)-C(41)-C(46)	118.8(5)
O(4)-C(41)-C(42)	120.8(5)
C(46)-C(41)-C(42)	120.4(6)
C(43)-C(42)-C(41)	118.6(6)
C(43)-C(42)-C(40)	117.9(6)
C(41)-C(42)-C(40)	123.4(6)
C(42)-C(43)-C(44)	122.5(6)
C(42)-C(43)-H(43)	118.8
C(44)-C(43)-H(43)	118.8
C(43)-C(44)-C(45)	117.3(6)
C(43)-C(44)-C(80)	119.7(6)
C(45)-C(44)-C(80)	123.0(6)
C(46)-C(45)-C(44)	121.4(6)
C(46)-C(45)-H(45)	119.3
C(44)-C(45)-H(45)	119.3
C(41)-C(46)-C(45)	119.6(6)
C(41)-C(46)-C(10)	119.9(6)
C(45)-C(46)-C(10)	120.4(6)

O(4)-C(47)-C(48)	108.7(6)
O(4)-C(47)-H(47A)	109.9
C(48)-C(47)-H(47A)	109.9
O(4)-C(47)-H(47B)	109.9
C(48)-C(47)-H(47B)	109.9
H(47A)-C(47)-H(47B)	108.3
C(47)-C(48)-C(49)	114.4(9)
C(47)-C(48)-H(48A)	108.7
C(49)-C(48)-H(48A)	108.7
C(47)-C(48)-H(48B)	108.7
C(49)-C(48)-H(48B)	108.7
H(48A)-C(48)-H(48B)	107.6
C(48)-C(49)-H(49A)	109.5
C(48)-C(49)-H(49B)	109.5
H(49A)-C(49)-H(49B)	109.5
C(48)-C(49)-H(49C)	109.5
H(49A)-C(49)-H(49C)	109.5
H(49B)-C(49)-H(49C)	109.5
H(49D)-C(49A)-H(49E)	109.5
H(49D)-C(49A)-H(49F)	109.5
H(49E)-C(49A)-H(49F)	109.5
C(56)-C(51)-C(52)	119.1(7)
C(56)-C(51)-C(14)	117.9(6)
C(52)-C(51)-C(14)	122.9(6)
O(5)-C(52)-C(53)	124.0(6)
O(5)-C(52)-C(51)	116.8(6)
C(53)-C(52)-C(51)	119.2(6)
C(52)-C(53)-C(54)	122.6(7)
C(52)-C(53)-H(53)	118.7
C(54)-C(53)-H(53)	118.7
C(55)-C(54)-C(53)	117.6(7)
C(55)-C(54)-C(58)	119.8(8)
C(53)-C(54)-C(58)	122.6(8)
C(54)-C(55)-O(6)	116.3(8)
C(54)-C(55)-C(56)	120.8(7)
O(6)-C(55)-C(56)	122.9(8)

C(51)-C(56)-C(55)	120.7(7)
C(51)-C(56)-H(56)	119.6
C(55)-C(56)-H(56)	119.6
O(5)-C(57)-H(57A)	109.5
O(5)-C(57)-H(57B)	109.5
H(57A)-C(57)-H(57B)	109.5
O(5)-C(57)-H(57C)	109.5
H(57A)-C(57)-H(57C)	109.5
H(57B)-C(57)-H(57C)	109.5
C(54)-C(58)-H(58A)	109.5
C(54)-C(58)-H(58B)	109.5
H(58A)-C(58)-H(58B)	109.5
C(54)-C(58)-H(58C)	109.5
H(58A)-C(58)-H(58C)	109.5
H(58B)-C(58)-H(58C)	109.5
O(6)-C(59)-H(59A)	109.5
O(6)-C(59)-H(59B)	109.5
H(59A)-C(59)-H(59B)	109.5
O(6)-C(59)-H(59C)	109.5
H(59A)-C(59)-H(59C)	109.5
H(59B)-C(59)-H(59C)	109.5
C(61)-C(60)-C(62)	114.2(10)
C(61)-C(60)-C(24)	111.5(8)
C(62)-C(60)-C(24)	107.5(8)
C(61)-C(60)-C(63)	111.6(9)
C(62)-C(60)-C(63)	106.3(9)
C(24)-C(60)-C(63)	105.2(7)
C(60)-C(61)-H(61A)	109.5
C(60)-C(61)-H(61B)	109.5
H(61A)-C(61)-H(61B)	109.5
C(60)-C(61)-H(61C)	109.5
H(61A)-C(61)-H(61C)	109.5
H(61B)-C(61)-H(61C)	109.5
C(60)-C(62)-H(62A)	109.5
C(60)-C(62)-H(62B)	109.5
H(62A)-C(62)-H(62B)	109.5

C(60)-C(62)-H(62C)	109.5
H(62A)-C(62)-H(62C)	109.5
H(62B)-C(62)-H(62C)	109.5
C(60)-C(63)-H(63A)	109.5
C(60)-C(63)-H(63B)	109.5
H(63A)-C(63)-H(63B)	109.5
C(60)-C(63)-H(63C)	109.5
H(63A)-C(63)-H(63C)	109.5
H(63B)-C(63)-H(63C)	109.5
H(61D)-C(61A)-H(61E)	109.5
H(61D)-C(61A)-H(61F)	109.5
H(61E)-C(61A)-H(61F)	109.5
H(62D)-C(62A)-H(62E)	109.5
H(62D)-C(62A)-H(62F)	109.5
H(62E)-C(62A)-H(62F)	109.5
H(63D)-C(63A)-H(63E)	109.5
H(63D)-C(63A)-H(63F)	109.5
H(63E)-C(63A)-H(63F)	109.5
C(76)-C(71)-C(72)	118.6(6)
C(76)-C(71)-C(34)	119.9(6)
C(72)-C(71)-C(34)	121.3(6)
O(7)-C(72)-C(71)	118.5(7)
O(7)-C(72)-C(73)	122.4(7)
C(71)-C(72)-C(73)	119.1(7)
C(74)-C(73)-C(72)	122.8(8)
C(74)-C(73)-H(73)	118.6
C(72)-C(73)-H(73)	118.6
C(73)-C(74)-C(75)	116.6(7)
C(73)-C(74)-C(78)	123.8(8)
C(75)-C(74)-C(78)	119.6(8)
C(76)-C(75)-O(8)	124.6(7)
C(76)-C(75)-C(74)	121.1(7)
O(8)-C(75)-C(74)	114.3(7)
C(71)-C(76)-C(75)	121.8(7)
C(71)-C(76)-H(76)	119.1
C(75)-C(76)-H(76)	119.1

O(7)-C(77)-H(77A)	109.5
O(7)-C(77)-H(77B)	109.5
H(77A)-C(77)-H(77B)	109.5
O(7)-C(77)-H(77C)	109.5
H(77A)-C(77)-H(77C)	109.5
H(77B)-C(77)-H(77C)	109.5
C(74)-C(78)-H(78A)	109.5
C(74)-C(78)-H(78B)	109.5
H(78A)-C(78)-H(78B)	109.5
C(74)-C(78)-H(78C)	109.5
H(78A)-C(78)-H(78C)	109.5
H(78B)-C(78)-H(78C)	109.5
O(8)-C(79)-H(79A)	109.5
O(8)-C(79)-H(79B)	109.5
H(79A)-C(79)-H(79B)	109.5
O(8)-C(79)-H(79C)	109.5
H(79A)-C(79)-H(79C)	109.5
H(79B)-C(79)-H(79C)	109.5
C(82)-C(80)-C(83)	111.4(8)
C(82)-C(80)-C(44)	109.7(6)
C(83)-C(80)-C(44)	110.5(6)
C(82)-C(80)-C(81)	108.2(8)
C(83)-C(80)-C(81)	106.3(7)
C(44)-C(80)-C(81)	110.7(7)
C(80)-C(81)-H(81A)	109.5
C(80)-C(81)-H(81B)	109.5
H(81A)-C(81)-H(81B)	109.5
C(80)-C(81)-H(81C)	109.5
H(81A)-C(81)-H(81C)	109.5
H(81B)-C(81)-H(81C)	109.5
C(80)-C(82)-H(82A)	109.5
C(80)-C(82)-H(82B)	109.5
H(82A)-C(82)-H(82B)	109.5
C(80)-C(82)-H(82C)	109.5
H(82A)-C(82)-H(82C)	109.5
H(82B)-C(82)-H(82C)	109.5

C(80)-C(83)-H(83A)	109.5
C(80)-C(83)-H(83B)	109.5
H(83A)-C(83)-H(83B)	109.5
C(80)-C(83)-H(83C)	109.5
H(83A)-C(83)-H(83C)	109.5
H(83B)-C(83)-H(83C)	109.5

Symmetry transformations used to generate equivalent atoms:

#1 -x+1,-y,-z

Table S4. Anisotropic displacement parameters ($Å^2$x 10^3)for raj0f. The anisotropic displacement factor exponent takes the form: $-2\pi^2[h^2a^{*2}U^{11} + ... + 2 h k a^* b^* U^{12}]$

	U^{11}	U^{22}	U^{33}	U^{23}	U^{13}	U^{12}
O(1)	51(3)	45(3)	86(4)	-11(2)	-13(3)	5(2)
O(2)	42(2)	32(2)	47(2)	-1(2)	-5(2)	2(2)
O(3)	48(2)	37(2)	50(2)	1(2)	9(2)	1(2)
O(4)	49(2)	35(2)	57(3)	-2(2)	-5(2)	2(2)
O(5)	50(3)	46(2)	64(3)	-1(2)	-4(2)	-12(2)
O(6)	104(5)	98(5)	85(4)	23(4)	18(4)	-2(4)
O(7)	142(6)	71(4)	58(3)	5(3)	14(3)	-48(4)
O(8)	105(4)	76(4)	58(3)	-5(3)	18(3)	1(3)
C(10)	40(3)	49(4)	81(5)	-7(3)	-6(3)	5(3)
C(11)	38(3)	45(4)	78(5)	0(3)	-5(3)	-1(3)
C(12)	36(3)	41(3)	85(5)	0(3)	6(3)	-5(3)
C(13)	38(3)	41(3)	82(5)	2(3)	0(3)	-6(3)
C(14)	42(3)	43(4)	69(4)	4(3)	1(3)	-7(3)
C(15)	38(3)	40(3)	81(5)	-2(3)	4(3)	-4(3)
C(16)	36(3)	35(3)	68(4)	1(3)	0(3)	-4(2)
C(17)	100(7)	64(5)	75(6)	-1(4)	-3(5)	4(5)
C(18)	189(13)	91(8)	83(7)	7(6)	-15(8)	3(8)
C(18A)	189(13)	91(8)	83(7)	7(6)	-15(8)	3(8)
C(20)	48(4)	33(3)	72(4)	3(3)	4(3)	-5(3)
C(21)	43(3)	24(3)	50(3)	2(2)	1(3)	4(2)
C(22)	51(4)	32(3)	63(4)	-2(3)	6(3)	2(3)

C(23)	68(4)	35(3)	47(4)	7(3)	15(3)	1(3)
C(24)	54(4)	42(3)	49(4)	-1(3)	-3(3)	9(3)
C(25)	48(4)	33(3)	72(4)	2(3)	-5(3)	7(3)
C(26)	37(3)	35(3)	54(4)	4(3)	0(3)	5(2)
C(27)	58(4)	38(3)	57(4)	-7(3)	-1(3)	4(3)
C(28)	69(5)	55(4)	53(4)	-8(3)	0(3)	-10(3)
C(29)	96(6)	66(5)	63(5)	-3(4)	1(4)	-15(4)
C(30)	43(3)	40(3)	48(3)	1(3)	0(3)	6(3)
C(31)	44(3)	40(3)	49(4)	-2(3)	1(3)	-8(3)
C(32)	33(3)	29(3)	48(3)	-2(2)	0(2)	-2(2)
C(33)	37(3)	44(3)	52(4)	-1(3)	4(3)	-4(3)
C(34)	47(4)	46(3)	51(4)	0(3)	6(3)	-11(3)
C(35)	54(4)	35(3)	56(4)	8(3)	0(3)	-6(3)
C(36)	41(3)	28(3)	59(4)	1(3)	1(3)	-7(2)
C(37)	58(4)	53(4)	53(4)	5(3)	8(3)	1(3)
C(38)	81(5)	62(4)	49(4)	2(3)	17(4)	6(4)
C(39)	145(9)	85(6)	61(5)	-2(5)	19(5)	-8(6)
C(40)	54(4)	41(3)	64(4)	0(3)	5(3)	-3(3)
C(41)	53(4)	28(3)	47(3)	1(2)	1(3)	2(3)
C(42)	49(3)	29(3)	55(4)	6(3)	-1(3)	8(3)
C(43)	52(4)	34(3)	58(4)	-5(3)	-9(3)	7(3)
C(44)	60(4)	41(3)	55(4)	0(3)	-2(3)	14(3)
C(45)	51(4)	46(4)	61(4)	-1(3)	7(3)	10(3)
C(46)	48(4)	32(3)	62(4)	-2(3)	-4(3)	6(3)
C(47)	92(6)	46(4)	74(5)	1(4)	2(4)	-3(4)
C(48)	163(10)	59(5)	61(5)	-8(4)	19(6)	-19(6)
C(48A)	163(10)	59(5)	61(5)	-8(4)	19(6)	-19(6)
C(51)	46(4)	40(3)	74(5)	1(3)	1(3)	-3(3)
C(52)	53(4)	45(4)	53(4)	4(3)	0(3)	-4(3)
C(53)	57(4)	52(4)	69(5)	2(3)	-2(3)	1(3)
C(54)	75(5)	69(5)	58(4)	4(4)	-1(4)	4(4)
C(55)	63(5)	82(6)	81(6)	29(5)	1(4)	-7(4)
C(56)	63(5)	55(4)	75(5)	6(4)	1(4)	-1(3)
C(57)	50(4)	44(3)	63(4)	-6(3)	-6(3)	-6(3)
C(58)	94(7)	126(9)	80(6)	2(6)	10(5)	7(6)
C(59)	92(7)	132(10)	125(9)	56(8)	-7(6)	-38(7)

C(60)	93(6)	50(4)	49(4)	9(3)	9(4)	2(4)
C(60A)	93(6)	50(4)	49(4)	9(3)	9(4)	2(4)
C(71)	54(4)	51(4)	50(4)	10(3)	4(3)	0(3)
C(72)	103(6)	68(5)	51(4)	2(4)	1(4)	-33(5)
C(73)	104(7)	88(6)	80(6)	4(5)	28(5)	-37(5)
C(74)	109(7)	74(5)	62(5)	10(4)	9(5)	-15(5)
C(75)	86(5)	70(5)	48(4)	2(4)	8(4)	2(4)
C(76)	63(4)	56(4)	51(4)	5(3)	-2(3)	-6(3)
C(77)	168(10)	64(5)	92(7)	15(5)	-15(7)	-61(6)
C(78)	130(9)	135(9)	90(7)	42(7)	58(7)	14(7)
C(79)	109(7)	63(5)	60(5)	-13(4)	7(4)	-10(5)
C(80)	67(5)	60(4)	63(4)	4(3)	2(4)	18(4)
C(81)	81(6)	150(9)	64(5)	-28(6)	4(4)	37(6)
C(82)	166(10)	59(5)	76(6)	-20(4)	9(6)	29(6)
C(83)	102(7)	81(6)	60(5)	-14(4)	-12(4)	20(5)
Cl(1X)	130(6)	87(7)	96(5)	-6(4)	23(4)	14(4)

Table S5. Hydrogen coordinates (x 10^4) and isotropic displacement parameters (Å^{2}x 10^3) for raj0f.

	x	y	z	U(eq)
H(10A)	8832	592	-3146	68
H(10B)	8316	340	-2705	68
H(13)	8292	1219	-2248	65
H(15)	7168	3115	-2579	64
H(17A)	8392	1332	-3789	96
H(17B)	7832	2090	-3867	96
H(18A)	6501	904	-4198	146
H(18B)	5896	1650	-4265	146
H(19A)	6942	1291	-4831	145
H(19B)	8369	1172	-4564	145
H(19C)	7770	1921	-4630	145
H(18C)	7409	1080	-4378	146
H(18D)	5988	1098	-4139	146
H(19D)	5796	1515	-4728	108
H(19E)	7000	2054	-4616	108
H(19F)	5565	2072	-4376	108
H(20A)	6534	2936	-3587	61
H(20B)	5980	3351	-3200	61
H(23)	4682	2986	-4056	59
H(25)	926	2155	-3829	61
H(27A)	2712	3099	-2567	61
H(27B)	4275	3335	-2658	61
H(28A)	5140	2767	-2053	71
H(28B)	3979	3306	-1929	71
H(29A)	3644	2297	-1586	112
H(29B)	3494	1908	-2023	112
H(29C)	2335	2447	-1898	112
H(30A)	334	1829	-3177	52
H(30B)	1070	2233	-2799	52
H(33)	812	1508	-2250	53

H(35)	2109	-421	-2258	58
H(37A)	892	830	-3734	66
H(37B)	1424	70	-3655	66
H(38A)	2720	1016	-4192	76
H(38B)	3301	269	-4105	76
H(39A)	2025	293	-4739	145
H(39B)	679	567	-4510	145
H(39C)	1241	-182	-4418	145
H(40A)	3358	-875	-2792	63
H(40B)	2566	-695	-3225	63
H(43)	4135	-902	-3714	58
H(45)	7993	-68	-3697	63
H(47A)	6655	-358	-2274	85
H(47B)	5016	-535	-2274	85
H(48A)	4841	553	-1908	113
H(48B)	6475	447	-1806	113
H(49A)	5210	55	-1252	144
H(49B)	5976	-571	-1461	144
H(49C)	4341	-447	-1555	144
H(48C)	5822	-127	-1648	113
H(48D)	6367	534	-1865	113
H(49D)	4849	706	-1462	152
H(49E)	3788	221	-1718	152
H(49F)	4355	894	-1930	152
H(53)	9647	3592	-1330	72
H(56)	6892	1717	-1644	77
H(57A)	10882	4033	-2289	79
H(57B)	11245	3706	-1841	79
H(57C)	9966	4223	-1898	79
H(58A)	7635	3262	-520	149
H(58B)	9191	3518	-598	149
H(58C)	8934	2763	-440	149
H(59A)	5690	1287	-662	175
H(59B)	6612	1092	-1051	175
H(59C)	5300	1588	-1115	175
H(61A)	866	1970	-4519	116

H(61B)	532	2552	-4855	116
H(61C)	131	2655	-4379	116
H(62A)	3206	3690	-4466	109
H(62B)	1587	3750	-4362	109
H(62C)	2030	3628	-4832	109
H(63A)	4264	2656	-4680	91
H(63B)	3107	2518	-5044	91
H(63C)	3426	1956	-4694	91
H(61D)	3439	3571	-4584	189
H(61E)	2670	3273	-4997	189
H(61F)	3986	2885	-4787	189
H(62D)	1164	1827	-4527	104
H(62E)	2591	1842	-4772	104
H(62F)	1229	2217	-4964	104
H(63D)	828	3582	-4336	163
H(63E)	37	2870	-4330	163
H(63F)	384	3214	-4766	163
H(73)	-1201	-499	-1184	108
H(76)	1477	1352	-1548	68
H(77A)	-688	-1587	-2014	163
H(77B)	-1395	-1260	-1617	163
H(77C)	241	-1432	-1599	163
H(78A)	-255	365	-316	175
H(78B)	-1604	-34	-494	175
H(78C)	-1607	774	-483	175
H(79A)	1542	2293	-652	115
H(79B)	1129	2190	-1136	115
H(79C)	2416	1780	-927	115
H(81A)	7967	-977	-4615	147
H(81B)	8528	-925	-4139	147
H(81C)	7969	-264	-4379	147
H(82A)	5228	-1747	-4064	150
H(82B)	6862	-1815	-3944	150
H(82C)	6324	-1866	-4422	150
H(83A)	4382	-693	-4455	123
H(83B)	5513	-837	-4799	123

H(83C)	5505	-126	-4562	123

Table S1. Crystal data and structure refinement for raj0b.

Identification code	raj0b	
Empirical formula	C79.72 H93.59 N1.87 O12.19	
Formula weight	1273.08	
Temperature	100(2) K	
Wavelength	1.54178 Å	
Crystal system	Triclinic	
Space group	P -1	
Unit cell dimensions	a = 12.2609(3) Å	α= 102.5950(10)°.
	b = 16.8844(4) Å	β= 103.4940(10)°.
	c = 18.3910(5) Å	γ = 99.8310(10)°.
Volume	3513.16(15) $Å^3$	
Z	2	
Density (calculated)	1.203 Mg/m^3	
Absorption coefficient	0.641 mm^{-1}	
F(000)	1365	
Crystal size	0.65 x 0.25 x 0.16 mm^3	
Theta range for data collection	2.57 to 67.30°.	
Index ranges	-14<=h<=14, -20<=k<=19, 0<=l<=21	
Reflections collected	29139	
Independent reflections	11567 [R(int) = 0.0158]	
Completeness to theta = 67.30°	91.7 %	
Absorption correction	Numerical	
Max. and min. transmission	0.9044 and 0.6807	
Refinement method	Full-matrix least-squares on F^2	
Data / restraints / parameters	11567 / 93 / 982	
Goodness-of-fit on F^2	0.989	
Final R indices [I>2sigma(I)]	R1 = 0.0465, wR2 = 0.1205	
R indices (all data)	R1 = 0.0515, wR2 = 0.1242	
Extinction coefficient	0.00045(8)	
Largest diff. peak and hole	0.406 and -0.330 e.$Å^{-3}$	

Table S2. Atomic coordinates (x 10^4) and equivalent isotropic displacement parameters ($Å^2$x 10^3) for raj0b. U(eq) is defined as one third of the trace of the orthogonalized U^{ij} tensor.

	x	y	z	U(eq)
C(10)	14283(1)	9957(1)	3165(1)	25(1)
C(11)	14356(1)	8974(1)	1914(1)	25(1)
C(12)	14742(1)	9275(1)	2723(1)	25(1)
C(13)	15501(1)	8894(1)	3131(1)	26(1)
C(14)	15876(1)	8219(1)	2767(1)	27(1)
C(15)	15438(1)	7916(1)	1960(1)	26(1)
C(16)	14666(1)	8271(1)	1529(1)	25(1)
O(1)	13623(1)	9353(1)	1492(1)	31(1)
C(17)	14175(2)	9863(2)	1077(2)	30(1)
C(18)	15043(3)	10654(2)	1585(2)	42(1)
C(19)	15609(2)	11142(2)	1103(2)	40(1)
O(1A)	13623(1)	9353(1)	1492(1)	31(1)
C(17A)	14120(12)	10079(9)	1327(8)	76(5)
C(18A)	13142(10)	10139(9)	656(7)	87(4)
C(19A)	11970(10)	10166(9)	840(7)	77(3)
C(20)	14084(1)	7842(1)	672(1)	26(1)
C(21)	11926(1)	7227(1)	393(1)	24(1)
C(22)	13064(1)	7137(1)	586(1)	24(1)
C(23)	13261(1)	6398(1)	744(1)	25(1)
C(24)	12363(1)	5756(1)	724(1)	24(1)
C(25)	11254(1)	5893(1)	585(1)	23(1)
C(26)	11016(1)	6627(1)	433(1)	23(1)
O(2)	11697(1)	7924(1)	159(1)	31(1)
C(27)	11399(2)	7756(1)	-671(1)	45(1)
C(28)	11120(6)	8531(3)	-867(3)	63(1)
C(29)	10877(4)	8373(3)	-1773(2)	85(1)
C(27A)	11399(2)	7756(1)	-671(1)	45(1)
C(28A)	11280(20)	8443(13)	-1043(12)	63(1)
C(29A)	10320(20)	8856(14)	-727(14)	154(9)
C(30)	9813(1)	6783(1)	364(1)	23(1)
C(31)	9728(1)	7743(1)	1622(1)	23(1)

C(32)	9598(1)	6937(1)	1161(1)	23(1)
C(33)	9382(1)	6268(1)	1482(1)	24(1)
C(34)	9383(1)	6391(1)	2257(1)	24(1)
C(35)	9597(1)	7207(1)	2713(1)	24(1)
C(36)	9770(1)	7889(1)	2409(1)	23(1)
O(3)	9861(1)	8415(1)	1306(1)	28(1)
C(37)	8818(7)	8484(12)	801(4)	37(1)
C(38)	8138(2)	8943(2)	1282(2)	36(1)
C(39)	7054(2)	9045(2)	740(2)	44(1)
O(3A)	9861(1)	8415(1)	1306(1)	28(1)
C(37A)	8770(20)	8440(40)	814(13)	37(1)
C(38A)	7706(9)	8547(7)	1090(6)	45(3)
C(39A)	7852(9)	9447(6)	1484(6)	55(3)
C(40)	10081(1)	8766(1)	2946(1)	24(1)
C(41)	12188(1)	9446(1)	3140(1)	24(1)
C(42)	11351(1)	9008(1)	3404(1)	23(1)
C(43)	11710(1)	8746(1)	4069(1)	24(1)
C(44)	12879(1)	8890(1)	4461(1)	24(1)
C(45)	13686(1)	9305(1)	4167(1)	25(1)
C(46)	13366(1)	9579(1)	3504(1)	23(1)
O(4)	11857(1)	9720(1)	2485(1)	33(1)
C(47)	11439(5)	10528(3)	2685(3)	40(1)
C(48)	12363(5)	11303(3)	3087(3)	61(1)
C(49)	11851(5)	12077(3)	3239(3)	68(2)
O(4A)	11857(1)	9720(1)	2485(1)	33(1)
C(47A)	11721(4)	10495(3)	2561(3)	35(1)
C(48A)	11885(3)	10796(2)	1894(2)	39(1)
C(49A)	11175(4)	10224(3)	1105(2)	42(1)
C(51)	16698(2)	7862(2)	3244(2)	26(1)
C(52)	16626(2)	7001(1)	3115(1)	28(1)
C(53)	17375(2)	6706(1)	3630(1)	31(1)
C(54)	18200(2)	7240(2)	4280(1)	32(1)
C(55)	18269(2)	8097(1)	4409(1)	32(1)
C(56)	17542(2)	8397(2)	3898(1)	29(1)
O(5)	15783(1)	6478(1)	2476(1)	34(1)
O(6)	19092(1)	8609(1)	5066(1)	42(1)

C(57)	15454(2)	5628(1)	2485(1)	36(1)
C(58)	18989(2)	6915(2)	4842(1)	44(1)
C(59)	18898(2)	9411(2)	5340(2)	54(1)
C(51A)	16570(20)	7633(13)	3267(17)	26(1)
C(52A)	17532(16)	8035(9)	3909(10)	28(1)
C(53A)	18103(19)	7561(11)	4345(12)	31(1)
C(54A)	17714(14)	6696(10)	4136(9)	32(1)
C(55A)	16752(14)	6307(9)	3490(9)	32(1)
C(56A)	16204(17)	6774(11)	3045(13)	29(1)
O(5A)	17944(11)	8889(8)	4072(7)	34(1)
O(6A)	16388(11)	5450(7)	3328(7)	42(1)
C(57A)	18432(17)	9304(11)	4866(9)	36(1)
C(58A)	18257(18)	6167(12)	4618(11)	44(1)
C(59A)	15458(17)	5058(14)	2673(12)	54(1)
C(61)	12566(1)	4954(1)	872(1)	25(1)
C(62)	13111(1)	4456(1)	433(1)	28(1)
C(63)	13261(1)	3701(1)	581(1)	31(1)
C(64)	12867(2)	3420(1)	1149(1)	32(1)
C(65)	12301(2)	3911(1)	1577(1)	31(1)
C(66)	12161(1)	4668(1)	1440(1)	28(1)
O(7)	13437(1)	4740(1)	-151(1)	34(1)
O(8)	11910(1)	3587(1)	2119(1)	42(1)
C(67)	13789(2)	4167(1)	-690(1)	40(1)
C(68)	13012(2)	2595(1)	1296(1)	45(1)
C(69)	11290(2)	4060(1)	2547(1)	47(1)
C(71)	9183(5)	5669(3)	2588(3)	24(1)
C(72)	9888(10)	5631(4)	3295(5)	27(1)
C(73)	9673(3)	4915(2)	3551(2)	31(1)
C(74)	8781(2)	4230(2)	3127(2)	31(1)
C(75)	8094(2)	4259(2)	2416(1)	28(1)
C(76)	8301(4)	4968(3)	2154(3)	25(1)
O(9)	10775(1)	6316(1)	3694(1)	32(1)
O(10)	7224(1)	3558(1)	2019(1)	36(1)
C(77)	11567(2)	6257(1)	4378(1)	36(1)
C(78)	8582(2)	3466(2)	3419(2)	44(1)
C(79)	6449(2)	3596(1)	1332(1)	35(1)

C(71A)	9230(50)	5580(30)	2530(30)	24(1)
C(72A)	8300(40)	4870(30)	2220(20)	27(1)
C(73A)	8230(20)	4272(18)	2631(14)	31(1)
C(74A)	9070(20)	4304(18)	3274(18)	31(1)
C(75A)	9890(30)	5050(20)	3633(17)	28(1)
C(76A)	9940(80)	5700(30)	3280(40)	25(1)
O(9A)	7525(9)	4862(6)	1551(6)	32(1)
O(10A)	10648(9)	5092(7)	4334(6)	36(1)
C(77A)	6657(13)	4107(10)	1155(9)	36(1)
C(78A)	8940(20)	3615(15)	3682(14)	44(1)
C(79A)	11626(13)	5771(9)	4658(9)	35(1)
C(81)	13295(6)	8557(6)	5125(4)	25(1)
C(82)	12875(3)	8656(2)	5777(2)	28(1)
C(83)	13274(2)	8276(2)	6359(1)	31(1)
C(84)	14092(2)	7809(1)	6319(2)	30(1)
C(85)	14527(2)	7726(1)	5678(1)	30(1)
C(86)	14127(3)	8088(2)	5091(2)	28(1)
O(11)	12108(1)	9160(1)	5813(1)	34(1)
O(12)	15349(1)	7259(1)	5677(1)	40(1)
C(87)	11784(2)	9350(2)	6511(2)	47(1)
C(88)	14514(2)	7398(2)	6946(1)	44(1)
C(89)	15794(3)	7142(2)	5021(2)	55(1)
C(81A)	13170(40)	8520(40)	5180(20)	25(1)
C(82A)	14037(17)	8064(14)	5272(9)	28(1)
C(83A)	14337(13)	7836(9)	5968(8)	31(1)
C(84A)	13870(12)	8080(9)	6557(7)	30(1)
C(85A)	13020(12)	8527(9)	6454(7)	30(1)
C(86A)	12670(20)	8756(17)	5773(11)	28(1)
O(11A)	14501(7)	7898(5)	4656(5)	34(1)
O(12A)	12522(7)	8717(5)	7057(5)	40(1)
C(87A)	15296(12)	7363(10)	4703(8)	47(1)
C(88A)	14237(12)	7825(9)	7304(7)	44(1)
C(89A)	11627(13)	9149(10)	6946(9)	55(1)
N(1S)	13368(2)	6174(1)	3249(1)	56(1)
C(1S)	13049(2)	6661(1)	2965(1)	35(1)
C(2S)	12637(2)	7275(1)	2605(1)	33(1)

N(2S)	13578(4)	5110(2)	5529(3)	111(1)
C(3S)	14576(4)	5316(2)	5848(2)	88(1)
C(4S)	15827(3)	5560(2)	6222(2)	83(1)
O(1W)	11378(7)	9707(5)	811(5)	51(3)

Table S3. Bond lengths [Å] and angles [°] for raj0b.

C(10)-C(46)	1.522(2)
C(10)-C(12)	1.526(2)
C(10)-H(10A)	0.9900
C(10)-H(10B)	0.9900
C(11)-O(1)	1.382(2)
C(11)-C(12)	1.397(2)
C(11)-C(16)	1.403(2)
C(12)-C(13)	1.389(2)
C(13)-C(14)	1.395(2)
C(13)-H(13)	0.9500
C(14)-C(15)	1.398(2)
C(14)-C(51)	1.475(3)
C(14)-C(51A)	1.69(2)
C(15)-C(16)	1.391(2)
C(15)-H(15)	0.9500
C(16)-C(20)	1.523(2)
O(1)-C(17)	1.453(3)
C(17)-C(18)	1.515(4)
C(17)-H(17A)	0.9900
C(17)-H(17B)	0.9900
C(18)-C(19)	1.535(3)
C(18)-H(18A)	0.9900
C(18)-H(18B)	0.9900
C(19)-H(19A)	0.9800
C(19)-H(19B)	0.9800
C(19)-H(19C)	0.9800
C(17A)-C(18A)	1.542(14)
C(17A)-H(17C)	0.9900
C(17A)-H(17D)	0.9900
C(18A)-C(19A)	1.556(14)
C(18A)-H(18C)	0.9900
C(18A)-H(18D)	0.9900
C(19A)-H(19D)	0.9800
C(19A)-H(19E)	0.9800

C(19A)-H(19F)	0.9800
C(20)-C(22)	1.525(2)
C(20)-H(20A)	0.9900
C(20)-H(20B)	0.9900
C(21)-O(2)	1.387(2)
C(21)-C(22)	1.401(2)
C(21)-C(26)	1.401(2)
C(22)-C(23)	1.388(2)
C(23)-C(24)	1.395(2)
C(23)-H(23)	0.9500
C(24)-C(25)	1.393(2)
C(24)-C(61)	1.488(2)
C(25)-C(26)	1.391(2)
C(25)-H(25)	0.9500
C(26)-C(30)	1.524(2)
O(2)-C(27)	1.436(2)
C(27)-C(28)	1.504(5)
C(27)-H(27A)	0.9900
C(27)-H(27B)	0.9900
C(28)-C(29)	1.576(5)
C(28)-H(28A)	0.9900
C(28)-H(28B)	0.9900
C(29)-H(29A)	0.9800
C(29)-H(29B)	0.9800
C(29)-H(29C)	0.9800
C(28A)-C(29A)	1.629(16)
C(28A)-H(28C)	0.9900
C(28A)-H(28D)	0.9900
C(29A)-H(29D)	0.9800
C(29A)-H(29E)	0.9800
C(29A)-H(29F)	0.9800
C(30)-C(32)	1.524(2)
C(30)-H(30A)	0.9900
C(30)-H(30B)	0.9900
C(31)-O(3)	1.3851(19)
C(31)-C(32)	1.398(2)

C(31)-C(36)	1.402(2)
C(32)-C(33)	1.398(2)
C(33)-C(34)	1.396(2)
C(33)-H(33)	0.9500
C(34)-C(35)	1.394(2)
C(34)-C(71)	1.485(4)
C(34)-C(71A)	1.55(3)
C(35)-C(36)	1.392(2)
C(35)-H(35)	0.9500
C(36)-C(40)	1.519(2)
O(3)-C(37)	1.433(5)
C(37)-C(38)	1.530(7)
C(37)-H(37A)	0.9900
C(37)-H(37B)	0.9900
C(38)-C(39)	1.524(3)
C(38)-H(38A)	0.9900
C(38)-H(38B)	0.9900
C(39)-H(39A)	0.9800
C(39)-H(39B)	0.9800
C(39)-H(39C)	0.9800
C(37A)-C(38A)	1.535(17)
C(37A)-H(37C)	0.9900
C(37A)-H(37D)	0.9900
C(38A)-C(39A)	1.494(12)
C(38A)-H(38C)	0.9900
C(38A)-H(38D)	0.9900
C(39A)-H(39D)	0.9800
C(39A)-H(39E)	0.9800
C(39A)-H(39F)	0.9800
C(40)-C(42)	1.524(2)
C(40)-H(40A)	0.9900
C(40)-H(40B)	0.9900
C(41)-O(4)	1.382(2)
C(41)-C(46)	1.399(2)
C(41)-C(42)	1.403(2)
C(42)-C(43)	1.392(2)

C(43)-C(44)	1.397(2)
C(43)-H(43)	0.9500
C(44)-C(45)	1.392(2)
C(44)-C(81)	1.475(4)
C(44)-C(81A)	1.58(2)
C(45)-C(46)	1.394(2)
C(45)-H(45)	0.9500
O(4)-C(47)	1.539(5)
C(47)-C(48)	1.491(7)
C(47)-H(47A)	0.9900
C(47)-H(47B)	0.9900
C(48)-C(49)	1.543(7)
C(48)-H(48A)	0.9900
C(48)-H(48B)	0.9900
C(49)-H(49A)	0.9800
C(49)-H(49B)	0.9800
C(49)-H(49C)	0.9800
C(47A)-C(48A)	1.467(6)
C(47A)-H(47C)	0.9900
C(47A)-H(47D)	0.9900
C(48A)-C(49A)	1.520(5)
C(48A)-H(48C)	0.9900
C(48A)-H(48D)	0.9900
C(49A)-H(49D)	0.9800
C(49A)-H(49E)	0.9800
C(49A)-H(49F)	0.9800
C(51)-C(56)	1.400(3)
C(51)-C(52)	1.405(3)
C(52)-O(5)	1.377(3)
C(52)-C(53)	1.395(3)
C(53)-C(54)	1.386(3)
C(53)-H(53)	0.9500
C(54)-C(55)	1.398(3)
C(54)-C(58)	1.506(3)
C(55)-O(6)	1.380(2)
C(55)-C(56)	1.381(3)

C(56)-H(56)	0.9500
O(5)-C(57)	1.428(2)
O(6)-C(59)	1.418(3)
C(57)-H(57A)	0.9800
C(57)-H(57B)	0.9800
C(57)-H(57C)	0.9800
C(58)-H(58A)	0.9800
C(58)-H(58B)	0.9800
C(58)-H(58C)	0.9800
C(59)-H(59A)	0.9800
C(59)-H(59B)	0.9800
C(59)-H(59C)	0.9800
C(51A)-C(56A)	1.382(18)
C(51A)-C(52A)	1.405(18)
C(52A)-O(5A)	1.384(14)
C(52A)-C(53A)	1.408(17)
C(53A)-C(54A)	1.394(16)
C(53A)-H(53A)	0.9500
C(54A)-C(55A)	1.405(15)
C(54A)-C(58A)	1.517(16)
C(55A)-O(6A)	1.381(15)
C(55A)-C(56A)	1.397(16)
C(56A)-H(56A)	0.9500
O(5A)-C(57A)	1.408(16)
O(6A)-C(59A)	1.394(15)
C(57A)-H(57D)	0.9800
C(57A)-H(57E)	0.9800
C(57A)-H(57F)	0.9800
C(58A)-H(58D)	0.9800
C(58A)-H(58E)	0.9800
C(58A)-H(58F)	0.9800
C(59A)-H(59D)	0.9800
C(59A)-H(59E)	0.9800
C(59A)-H(59F)	0.9800
C(61)-C(66)	1.397(2)
C(61)-C(62)	1.398(2)

C(62)-O(7)	1.378(2)
C(62)-C(63)	1.393(2)
C(63)-C(64)	1.386(3)
C(63)-H(63)	0.9500
C(64)-C(65)	1.399(3)
C(64)-C(68)	1.508(2)
C(65)-O(8)	1.375(2)
C(65)-C(66)	1.386(2)
C(66)-H(66)	0.9500
O(7)-C(67)	1.425(2)
O(8)-C(69)	1.430(2)
C(67)-H(67A)	0.9800
C(67)-H(67B)	0.9800
C(67)-H(67C)	0.9800
C(68)-H(68A)	0.9800
C(68)-H(68B)	0.9800
C(68)-H(68C)	0.9800
C(69)-H(69A)	0.9800
C(69)-H(69B)	0.9800
C(69)-H(69C)	0.9800
C(71)-C(76)	1.398(4)
C(71)-C(72)	1.403(4)
C(72)-O(9)	1.374(4)
C(72)-C(73)	1.397(3)
C(73)-C(74)	1.385(3)
C(73)-H(73)	0.9500
C(74)-C(75)	1.396(3)
C(74)-C(78)	1.506(3)
C(75)-O(10)	1.379(3)
C(75)-C(76)	1.391(3)
C(76)-H(76)	0.9500
O(9)-C(77)	1.433(2)
O(10)-C(79)	1.413(2)
C(77)-H(77A)	0.9800
C(77)-H(77B)	0.9800
C(77)-H(77C)	0.9800

C(78)-H(78A)	0.9800
C(78)-H(78B)	0.9800
C(78)-H(78C)	0.9800
C(79)-H(79A)	0.9800
C(79)-H(79B)	0.9800
C(79)-H(79C)	0.9800
C(71A)-C(76A)	1.40(2)
C(71A)-C(72A)	1.409(19)
C(72A)-O(9A)	1.365(17)
C(72A)-C(73A)	1.393(18)
C(73A)-C(74A)	1.358(17)
C(73A)-H(73A)	0.9500
C(74A)-C(75A)	1.391(17)
C(74A)-C(78A)	1.521(17)
C(75A)-O(10A)	1.381(17)
C(75A)-C(76A)	1.389(19)
C(76A)-H(76A)	0.9500
O(9A)-C(77A)	1.435(14)
O(10A)-C(79A)	1.416(14)
C(77A)-H(77D)	0.9800
C(77A)-H(77E)	0.9800
C(77A)-H(77F)	0.9800
C(78A)-H(78D)	0.9800
C(78A)-H(78E)	0.9800
C(78A)-H(78F)	0.9800
C(79A)-H(79D)	0.9800
C(79A)-H(79E)	0.9800
C(79A)-H(79F)	0.9800
C(81)-C(86)	1.399(4)
C(81)-C(82)	1.400(4)
C(82)-O(11)	1.376(3)
C(82)-C(83)	1.400(4)
C(83)-C(84)	1.382(4)
C(83)-H(83)	0.9500
C(84)-C(85)	1.393(3)
C(84)-C(88)	1.509(3)

C(85)-O(12)	1.381(3)
C(85)-C(86)	1.387(3)
C(86)-H(86)	0.9500
O(11)-C(87)	1.418(3)
O(12)-C(89)	1.425(3)
C(87)-H(87A)	0.9800
C(87)-H(87B)	0.9800
C(87)-H(87C)	0.9800
C(88)-H(88A)	0.9800
C(88)-H(88B)	0.9800
C(88)-H(88C)	0.9800
C(89)-H(89A)	0.9800
C(89)-H(89B)	0.9800
C(89)-H(89C)	0.9800
C(81A)-C(86A)	1.39(2)
C(81A)-C(82A)	1.414(19)
C(82A)-O(11A)	1.378(13)
C(82A)-C(83A)	1.402(16)
C(83A)-C(84A)	1.360(14)
C(83A)-H(83A)	0.9500
C(84A)-C(85A)	1.389(13)
C(84A)-C(88A)	1.519(13)
C(85A)-C(86A)	1.387(16)
C(85A)-O(12A)	1.390(12)
C(86A)-H(86A)	0.9500
O(11A)-C(87A)	1.438(13)
O(12A)-C(89A)	1.417(12)
C(87A)-H(87D)	0.9800
C(87A)-H(87E)	0.9800
C(87A)-H(87F)	0.9800
C(88A)-H(88D)	0.9800
C(88A)-H(88E)	0.9800
C(88A)-H(88F)	0.9800
C(89A)-H(89D)	0.9800
C(89A)-H(89E)	0.9800
C(89A)-H(89F)	0.9800

N(1S)-C(1S)	1.142(3)
C(1S)-C(2S)	1.446(3)
C(2S)-H(2S1)	0.96(5)
C(2S)-H(2S2)	1.00(5)
C(2S)-H(2S3)	0.99(5)
C(2S)-H(2S4)	0.95(6)
C(2S)-H(2S5)	0.96(6)
C(2S)-H(2S6)	0.96(6)
N(2S)-C(3S)	1.180(5)
C(3S)-C(4S)	1.471(6)
C(4S)-H(4S1)	0.9800
C(4S)-H(4S2)	0.9800
C(4S)-H(4S3)	0.9800

C(46)-C(10)-C(12)	110.55(13)
C(46)-C(10)-H(10A)	109.5
C(12)-C(10)-H(10A)	109.5
C(46)-C(10)-H(10B)	109.5
C(12)-C(10)-H(10B)	109.5
H(10A)-C(10)-H(10B)	108.1
O(1)-C(11)-C(12)	119.58(14)
O(1)-C(11)-C(16)	119.59(14)
C(12)-C(11)-C(16)	120.78(15)
C(13)-C(12)-C(11)	118.29(15)
C(13)-C(12)-C(10)	119.72(15)
C(11)-C(12)-C(10)	121.83(15)
C(12)-C(13)-C(14)	122.72(16)
C(12)-C(13)-H(13)	118.6
C(14)-C(13)-H(13)	118.6
C(13)-C(14)-C(15)	117.36(15)
C(13)-C(14)-C(51)	119.12(18)
C(15)-C(14)-C(51)	123.52(19)
C(13)-C(14)-C(51A)	122.4(12)
C(15)-C(14)-C(51A)	118.7(10)
C(51)-C(14)-C(51A)	12.5(7)
C(16)-C(15)-C(14)	121.89(15)

C(16)-C(15)-H(15)	119.1
C(14)-C(15)-H(15)	119.1
C(15)-C(16)-C(11)	118.78(15)
C(15)-C(16)-C(20)	119.66(15)
C(11)-C(16)-C(20)	121.26(15)
C(11)-O(1)-C(17)	113.74(15)
O(1)-C(17)-C(18)	115.1(2)
O(1)-C(17)-H(17A)	108.5
C(18)-C(17)-H(17A)	108.5
O(1)-C(17)-H(17B)	108.5
C(18)-C(17)-H(17B)	108.5
H(17A)-C(17)-H(17B)	107.5
C(17)-C(18)-C(19)	111.8(2)
C(17)-C(18)-H(18A)	109.3
C(19)-C(18)-H(18A)	109.3
C(17)-C(18)-H(18B)	109.3
C(19)-C(18)-H(18B)	109.3
H(18A)-C(18)-H(18B)	107.9
C(18)-C(19)-H(19A)	109.5
C(18)-C(19)-H(19B)	109.5
H(19A)-C(19)-H(19B)	109.5
C(18)-C(19)-H(19C)	109.5
H(19A)-C(19)-H(19C)	109.5
H(19B)-C(19)-H(19C)	109.5
C(18A)-C(17A)-H(17C)	111.4
C(18A)-C(17A)-H(17D)	111.4
H(17C)-C(17A)-H(17D)	109.3
C(17A)-C(18A)-C(19A)	115.4(11)
C(17A)-C(18A)-H(18C)	108.4
C(19A)-C(18A)-H(18C)	108.4
C(17A)-C(18A)-H(18D)	108.4
C(19A)-C(18A)-H(18D)	108.4
H(18C)-C(18A)-H(18D)	107.5
C(18A)-C(19A)-H(19D)	109.5
C(18A)-C(19A)-H(19E)	109.5
H(19D)-C(19A)-H(19E)	109.5

C(18A)-C(19A)-H(19F)	109.5
H(19D)-C(19A)-H(19F)	109.5
H(19E)-C(19A)-H(19F)	109.5
C(16)-C(20)-C(22)	109.69(13)
C(16)-C(20)-H(20A)	109.7
C(22)-C(20)-H(20A)	109.7
C(16)-C(20)-H(20B)	109.7
C(22)-C(20)-H(20B)	109.7
H(20A)-C(20)-H(20B)	108.2
O(2)-C(21)-C(22)	119.45(15)
O(2)-C(21)-C(26)	119.49(14)
C(22)-C(21)-C(26)	121.06(15)
C(23)-C(22)-C(21)	118.47(15)
C(23)-C(22)-C(20)	119.64(15)
C(21)-C(22)-C(20)	121.79(15)
C(22)-C(23)-C(24)	121.85(15)
C(22)-C(23)-H(23)	119.1
C(24)-C(23)-H(23)	119.1
C(25)-C(24)-C(23)	117.95(15)
C(25)-C(24)-C(61)	119.99(15)
C(23)-C(24)-C(61)	122.04(15)
C(26)-C(25)-C(24)	122.20(15)
C(26)-C(25)-H(25)	118.9
C(24)-C(25)-H(25)	118.9
C(25)-C(26)-C(21)	118.03(15)
C(25)-C(26)-C(30)	120.08(14)
C(21)-C(26)-C(30)	121.80(14)
C(21)-O(2)-C(27)	110.97(14)
O(2)-C(27)-C(28)	106.9(2)
O(2)-C(27)-H(27A)	110.3
C(28)-C(27)-H(27A)	110.3
O(2)-C(27)-H(27B)	110.3
C(28)-C(27)-H(27B)	110.3
H(27A)-C(27)-H(27B)	108.6
C(27)-C(28)-C(29)	107.1(3)
C(27)-C(28)-H(28A)	110.3

C(29)-C(28)-H(28A)	110.3
C(27)-C(28)-H(28B)	110.3
C(29)-C(28)-H(28B)	110.3
H(28A)-C(28)-H(28B)	108.5
C(28)-C(29)-H(29A)	109.5
C(28)-C(29)-H(29B)	109.5
H(29A)-C(29)-H(29B)	109.5
C(28)-C(29)-H(29C)	109.5
H(29A)-C(29)-H(29C)	109.5
H(29B)-C(29)-H(29C)	109.5
C(29A)-C(28A)-H(28C)	110.8
C(29A)-C(28A)-H(28D)	110.8
H(28C)-C(28A)-H(28D)	108.9
C(28A)-C(29A)-H(29D)	109.5
C(28A)-C(29A)-H(29E)	109.5
H(29D)-C(29A)-H(29E)	109.5
C(28A)-C(29A)-H(29F)	109.5
H(29D)-C(29A)-H(29F)	109.5
H(29E)-C(29A)-H(29F)	109.5
C(26)-C(30)-C(32)	109.34(12)
C(26)-C(30)-H(30A)	109.8
C(32)-C(30)-H(30A)	109.8
C(26)-C(30)-H(30B)	109.8
C(32)-C(30)-H(30B)	109.8
H(30A)-C(30)-H(30B)	108.3
O(3)-C(31)-C(32)	119.80(14)
O(3)-C(31)-C(36)	119.04(14)
C(32)-C(31)-C(36)	121.12(14)
C(33)-C(32)-C(31)	118.20(15)
C(33)-C(32)-C(30)	119.70(14)
C(31)-C(32)-C(30)	121.76(14)
C(34)-C(33)-C(32)	121.85(15)
C(34)-C(33)-H(33)	119.1
C(32)-C(33)-H(33)	119.1
C(35)-C(34)-C(33)	118.12(15)
C(35)-C(34)-C(71)	121.2(3)

C(33)-C(34)-C(71)	120.7(3)
C(35)-C(34)-C(71A)	127(2)
C(33)-C(34)-C(71A)	114(2)
C(71)-C(34)-C(71A)	7(3)
C(36)-C(35)-C(34)	121.88(15)
C(36)-C(35)-H(35)	119.1
C(34)-C(35)-H(35)	119.1
C(35)-C(36)-C(31)	118.52(15)
C(35)-C(36)-C(40)	119.39(14)
C(31)-C(36)-C(40)	121.95(14)
C(31)-O(3)-C(37)	114.2(8)
O(3)-C(37)-C(38)	109.7(5)
O(3)-C(37)-H(37A)	109.7
C(38)-C(37)-H(37A)	109.7
O(3)-C(37)-H(37B)	109.7
C(38)-C(37)-H(37B)	109.7
H(37A)-C(37)-H(37B)	108.2
C(39)-C(38)-C(37)	109.5(3)
C(39)-C(38)-H(38A)	109.8
C(37)-C(38)-H(38A)	109.8
C(39)-C(38)-H(38B)	109.8
C(37)-C(38)-H(38B)	109.8
H(38A)-C(38)-H(38B)	108.2
C(38)-C(39)-H(39A)	109.5
C(38)-C(39)-H(39B)	109.5
H(39A)-C(39)-H(39B)	109.5
C(38)-C(39)-H(39C)	109.5
H(39A)-C(39)-H(39C)	109.5
H(39B)-C(39)-H(39C)	109.5
C(38A)-C(37A)-H(37C)	106.2
C(38A)-C(37A)-H(37D)	106.2
H(37C)-C(37A)-H(37D)	106.4
C(39A)-C(38A)-C(37A)	109(2)
C(39A)-C(38A)-H(38C)	109.9
C(37A)-C(38A)-H(38C)	109.9
C(39A)-C(38A)-H(38D)	109.9

C(37A)-C(38A)-H(38D)	109.9
H(38C)-C(38A)-H(38D)	108.3
C(38A)-C(39A)-H(39D)	109.5
C(38A)-C(39A)-H(39E)	109.5
H(39D)-C(39A)-H(39E)	109.5
C(38A)-C(39A)-H(39F)	109.5
H(39D)-C(39A)-H(39F)	109.5
H(39E)-C(39A)-H(39F)	109.5
C(36)-C(40)-C(42)	110.62(13)
C(36)-C(40)-H(40A)	109.5
C(42)-C(40)-H(40A)	109.5
C(36)-C(40)-H(40B)	109.5
C(42)-C(40)-H(40B)	109.5
H(40A)-C(40)-H(40B)	108.1
O(4)-C(41)-C(46)	118.68(14)
O(4)-C(41)-C(42)	119.89(14)
C(46)-C(41)-C(42)	121.36(15)
C(43)-C(42)-C(41)	118.60(15)
C(43)-C(42)-C(40)	120.08(14)
C(41)-C(42)-C(40)	121.18(14)
C(42)-C(43)-C(44)	121.46(15)
C(42)-C(43)-H(43)	119.3
C(44)-C(43)-H(43)	119.3
C(45)-C(44)-C(43)	118.26(15)
C(45)-C(44)-C(81)	118.6(3)
C(43)-C(44)-C(81)	122.9(3)
C(45)-C(44)-C(81A)	125.6(18)
C(43)-C(44)-C(81A)	116.1(19)
C(81)-C(44)-C(81A)	7.1(16)
C(44)-C(45)-C(46)	122.25(15)
C(44)-C(45)-H(45)	118.9
C(46)-C(45)-H(45)	118.9
C(45)-C(46)-C(41)	117.96(14)
C(45)-C(46)-C(10)	120.14(14)
C(41)-C(46)-C(10)	121.77(14)
C(41)-O(4)-C(47)	110.5(2)

C(48)-C(47)-O(4)	115.7(4)
C(48)-C(47)-H(47A)	108.4
O(4)-C(47)-H(47A)	108.4
C(48)-C(47)-H(47B)	108.4
O(4)-C(47)-H(47B)	108.4
H(47A)-C(47)-H(47B)	107.4
C(47)-C(48)-C(49)	111.3(4)
C(47)-C(48)-H(48A)	109.4
C(49)-C(48)-H(48A)	109.4
C(47)-C(48)-H(48B)	109.4
C(49)-C(48)-H(48B)	109.4
H(48A)-C(48)-H(48B)	108.0
C(48)-C(49)-H(49A)	109.5
C(48)-C(49)-H(49B)	109.5
H(49A)-C(49)-H(49B)	109.5
C(48)-C(49)-H(49C)	109.5
H(49A)-C(49)-H(49C)	109.5
H(49B)-C(49)-H(49C)	109.5
C(48A)-C(47A)-H(47C)	109.4
C(48A)-C(47A)-H(47D)	109.4
H(47C)-C(47A)-H(47D)	108.0
C(47A)-C(48A)-C(49A)	115.0(3)
C(47A)-C(48A)-H(48C)	108.5
C(49A)-C(48A)-H(48C)	108.5
C(47A)-C(48A)-H(48D)	108.5
C(49A)-C(48A)-H(48D)	108.5
H(48C)-C(48A)-H(48D)	107.5
C(48A)-C(49A)-H(49D)	109.5
C(48A)-C(49A)-H(49E)	109.5
H(49D)-C(49A)-H(49E)	109.5
C(48A)-C(49A)-H(49F)	109.5
H(49D)-C(49A)-H(49F)	109.5
H(49E)-C(49A)-H(49F)	109.5
C(56)-C(51)-C(52)	117.6(2)
C(56)-C(51)-C(14)	118.8(2)
C(52)-C(51)-C(14)	123.4(2)

O(5)-C(52)-C(53)	122.67(19)
O(5)-C(52)-C(51)	117.17(18)
C(53)-C(52)-C(51)	120.2(2)
C(54)-C(53)-C(52)	121.8(2)
C(54)-C(53)-H(53)	119.1
C(52)-C(53)-H(53)	119.1
C(53)-C(54)-C(55)	117.95(19)
C(53)-C(54)-C(58)	121.5(2)
C(55)-C(54)-C(58)	120.6(2)
O(6)-C(55)-C(56)	122.9(2)
O(6)-C(55)-C(54)	116.33(18)
C(56)-C(55)-C(54)	120.78(19)
C(55)-C(56)-C(51)	121.7(2)
C(55)-C(56)-H(56)	119.2
C(51)-C(56)-H(56)	119.2
C(52)-O(5)-C(57)	116.53(15)
C(55)-O(6)-C(59)	115.39(16)
O(5)-C(57)-H(57A)	109.5
O(5)-C(57)-H(57B)	109.5
H(57A)-C(57)-H(57B)	109.5
O(5)-C(57)-H(57C)	109.5
H(57A)-C(57)-H(57C)	109.5
H(57B)-C(57)-H(57C)	109.5
O(6)-C(59)-H(59A)	109.5
O(6)-C(59)-H(59B)	109.5
H(59A)-C(59)-H(59B)	109.5
O(6)-C(59)-H(59C)	109.5
H(59A)-C(59)-H(59C)	109.5
H(59B)-C(59)-H(59C)	109.5
C(56A)-C(51A)-C(52A)	120.4(18)
C(56A)-C(51A)-C(14)	120.7(14)
C(52A)-C(51A)-C(14)	118.9(14)
O(5A)-C(52A)-C(51A)	119.0(14)
O(5A)-C(52A)-C(53A)	121.1(15)
C(51A)-C(52A)-C(53A)	119.8(15)
C(54A)-C(53A)-C(52A)	119.9(16)

C(54A)-C(53A)-H(53A)	120.1
C(52A)-C(53A)-H(53A)	120.1
C(53A)-C(54A)-C(55A)	119.3(13)
C(53A)-C(54A)-C(58A)	121.5(14)
C(55A)-C(54A)-C(58A)	119.0(14)
O(6A)-C(55A)-C(56A)	122.6(13)
O(6A)-C(55A)-C(54A)	116.5(12)
C(56A)-C(55A)-C(54A)	120.9(13)
C(51A)-C(56A)-C(55A)	119.6(17)
C(51A)-C(56A)-H(56A)	120.2
C(55A)-C(56A)-H(56A)	120.2
C(52A)-O(5A)-C(57A)	115.1(13)
C(55A)-O(6A)-C(59A)	116.8(14)
O(5A)-C(57A)-H(57D)	109.5
O(5A)-C(57A)-H(57E)	109.5
H(57D)-C(57A)-H(57E)	109.5
O(5A)-C(57A)-H(57F)	109.5
H(57D)-C(57A)-H(57F)	109.5
H(57E)-C(57A)-H(57F)	109.5
C(54A)-C(58A)-H(58D)	109.5
C(54A)-C(58A)-H(58E)	109.5
H(58D)-C(58A)-H(58E)	109.5
C(54A)-C(58A)-H(58F)	109.5
H(58D)-C(58A)-H(58F)	109.5
H(58E)-C(58A)-H(58F)	109.5
O(6A)-C(59A)-H(59D)	109.5
O(6A)-C(59A)-H(59E)	109.5
H(59D)-C(59A)-H(59E)	109.5
O(6A)-C(59A)-H(59F)	109.5
H(59D)-C(59A)-H(59F)	109.5
H(59E)-C(59A)-H(59F)	109.5
C(66)-C(61)-C(62)	118.65(15)
C(66)-C(61)-C(24)	119.64(14)
C(62)-C(61)-C(24)	121.65(15)
O(7)-C(62)-C(63)	123.67(15)
O(7)-C(62)-C(61)	116.67(15)

C(63)-C(62)-C(61)	119.62(16)
C(64)-C(63)-C(62)	121.76(16)
C(64)-C(63)-H(63)	119.1
C(62)-C(63)-H(63)	119.1
C(63)-C(64)-C(65)	118.54(16)
C(63)-C(64)-C(68)	121.50(17)
C(65)-C(64)-C(68)	119.94(18)
O(8)-C(65)-C(66)	124.34(16)
O(8)-C(65)-C(64)	115.59(15)
C(66)-C(65)-C(64)	120.07(17)
C(65)-C(66)-C(61)	121.32(16)
C(65)-C(66)-H(66)	119.3
C(61)-C(66)-H(66)	119.3
C(62)-O(7)-C(67)	116.71(14)
C(65)-O(8)-C(69)	116.94(14)
O(7)-C(67)-H(67A)	109.5
O(7)-C(67)-H(67B)	109.5
H(67A)-C(67)-H(67B)	109.5
O(7)-C(67)-H(67C)	109.5
H(67A)-C(67)-H(67C)	109.5
H(67B)-C(67)-H(67C)	109.5
C(64)-C(68)-H(68A)	109.5
C(64)-C(68)-H(68B)	109.5
H(68A)-C(68)-H(68B)	109.5
C(64)-C(68)-H(68C)	109.5
H(68A)-C(68)-H(68C)	109.5
H(68B)-C(68)-H(68C)	109.5
O(8)-C(69)-H(69A)	109.5
O(8)-C(69)-H(69B)	109.5
H(69A)-C(69)-H(69B)	109.5
O(8)-C(69)-H(69C)	109.5
H(69A)-C(69)-H(69C)	109.5
H(69B)-C(69)-H(69C)	109.5
C(76)-C(71)-C(72)	118.0(3)
C(76)-C(71)-C(34)	118.7(3)
C(72)-C(71)-C(34)	123.1(3)

O(9)-C(72)-C(73)	123.6(3)
O(9)-C(72)-C(71)	116.7(3)
C(73)-C(72)-C(71)	119.6(3)
C(74)-C(73)-C(72)	122.1(3)
C(74)-C(73)-H(73)	119.0
C(72)-C(73)-H(73)	119.0
C(73)-C(74)-C(75)	118.4(2)
C(73)-C(74)-C(78)	120.6(2)
C(75)-C(74)-C(78)	121.0(2)
O(10)-C(75)-C(76)	124.1(2)
O(10)-C(75)-C(74)	115.8(2)
C(76)-C(75)-C(74)	120.1(2)
C(75)-C(76)-C(71)	121.8(2)
C(75)-C(76)-H(76)	119.1
C(71)-C(76)-H(76)	119.1
C(72)-O(9)-C(77)	117.3(2)
C(75)-O(10)-C(79)	117.13(16)
O(9)-C(77)-H(77A)	109.5
O(9)-C(77)-H(77B)	109.5
H(77A)-C(77)-H(77B)	109.5
O(9)-C(77)-H(77C)	109.5
H(77A)-C(77)-H(77C)	109.5
H(77B)-C(77)-H(77C)	109.5
O(10)-C(79)-H(79A)	109.5
O(10)-C(79)-H(79B)	109.5
H(79A)-C(79)-H(79B)	109.5
O(10)-C(79)-H(79C)	109.5
H(79A)-C(79)-H(79C)	109.5
H(79B)-C(79)-H(79C)	109.5
C(76A)-C(71A)-C(72A)	118(2)
C(76A)-C(71A)-C(34)	112(2)
C(72A)-C(71A)-C(34)	128(3)
O(9A)-C(72A)-C(73A)	126(2)
O(9A)-C(72A)-C(71A)	115(2)
C(73A)-C(72A)-C(71A)	118.5(18)
C(74A)-C(73A)-C(72A)	123(2)

C(74A)-C(73A)-H(73A)	118.6
C(72A)-C(73A)-H(73A)	118.6
C(73A)-C(74A)-C(75A)	117.9(17)
C(73A)-C(74A)-C(78A)	119.5(18)
C(75A)-C(74A)-C(78A)	121.1(19)
O(10A)-C(75A)-C(76A)	124(2)
O(10A)-C(75A)-C(74A)	116.1(18)
C(76A)-C(75A)-C(74A)	120.2(18)
C(75A)-C(76A)-C(71A)	121(2)
C(75A)-C(76A)-H(76A)	119.7
C(71A)-C(76A)-H(76A)	119.7
C(72A)-O(9A)-C(77A)	117.7(16)
C(75A)-O(10A)-C(79A)	118.7(14)
O(9A)-C(77A)-H(77D)	109.5
O(9A)-C(77A)-H(77E)	109.5
H(77D)-C(77A)-H(77E)	109.5
O(9A)-C(77A)-H(77F)	109.5
H(77D)-C(77A)-H(77F)	109.5
H(77E)-C(77A)-H(77F)	109.5
C(74A)-C(78A)-H(78D)	109.5
C(74A)-C(78A)-H(78E)	109.5
H(78D)-C(78A)-H(78E)	109.5
C(74A)-C(78A)-H(78F)	109.5
H(78D)-C(78A)-H(78F)	109.5
H(78E)-C(78A)-H(78F)	109.5
O(10A)-C(79A)-H(79D)	109.5
O(10A)-C(79A)-H(79E)	109.5
H(79D)-C(79A)-H(79E)	109.5
O(10A)-C(79A)-H(79F)	109.5
H(79D)-C(79A)-H(79F)	109.5
H(79E)-C(79A)-H(79F)	109.5
C(86)-C(81)-C(82)	117.8(3)
C(86)-C(81)-C(44)	118.3(3)
C(82)-C(81)-C(44)	123.9(3)
O(11)-C(82)-C(83)	123.8(3)
O(11)-C(82)-C(81)	116.3(3)

C(83)-C(82)-C(81)	119.9(3)
C(84)-C(83)-C(82)	122.0(2)
C(84)-C(83)-H(83)	119.0
C(82)-C(83)-H(83)	119.0
C(83)-C(84)-C(85)	118.23(19)
C(83)-C(84)-C(88)	121.7(2)
C(85)-C(84)-C(88)	120.1(2)
O(12)-C(85)-C(86)	124.2(2)
O(12)-C(85)-C(84)	115.34(19)
C(86)-C(85)-C(84)	120.4(2)
C(85)-C(86)-C(81)	121.7(2)
C(85)-C(86)-H(86)	119.1
C(81)-C(86)-H(86)	119.1
C(82)-O(11)-C(87)	116.99(19)
C(85)-O(12)-C(89)	116.94(18)
C(86A)-C(81A)-C(82A)	121.0(18)
C(86A)-C(81A)-C(44)	117.4(18)
C(82A)-C(81A)-C(44)	120.9(17)
O(11A)-C(82A)-C(83A)	127.1(15)
O(11A)-C(82A)-C(81A)	115.4(15)
C(83A)-C(82A)-C(81A)	117.5(14)
C(84A)-C(83A)-C(82A)	122.0(13)
C(84A)-C(83A)-H(83A)	119.0
C(82A)-C(83A)-H(83A)	119.0
C(83A)-C(84A)-C(85A)	119.3(11)
C(83A)-C(84A)-C(88A)	119.9(12)
C(85A)-C(84A)-C(88A)	120.7(11)
C(86A)-C(85A)-C(84A)	121.5(13)
C(86A)-C(85A)-O(12A)	121.4(13)
C(84A)-C(85A)-O(12A)	117.0(10)
C(85A)-C(86A)-C(81A)	118.6(17)
C(85A)-C(86A)-H(86A)	120.7
C(81A)-C(86A)-H(86A)	120.7
C(82A)-O(11A)-C(87A)	116.1(11)
C(85A)-O(12A)-C(89A)	117.4(9)
O(11A)-C(87A)-H(87D)	109.5

O(11A)-C(87A)-H(87E)	109.5
H(87D)-C(87A)-H(87E)	109.5
O(11A)-C(87A)-H(87F)	109.5
H(87D)-C(87A)-H(87F)	109.5
H(87E)-C(87A)-H(87F)	109.5
C(84A)-C(88A)-H(88D)	109.5
C(84A)-C(88A)-H(88E)	109.5
H(88D)-C(88A)-H(88E)	109.5
C(84A)-C(88A)-H(88F)	109.5
H(88D)-C(88A)-H(88F)	109.5
H(88E)-C(88A)-H(88F)	109.5
O(12A)-C(89A)-H(89D)	109.5
O(12A)-C(89A)-H(89E)	109.5
H(89D)-C(89A)-H(89E)	109.5
O(12A)-C(89A)-H(89F)	109.5
H(89D)-C(89A)-H(89F)	109.5
H(89E)-C(89A)-H(89F)	109.5
N(1S)-C(1S)-C(2S)	179.6(2)
C(1S)-C(2S)-H(2S1)	112(3)
C(1S)-C(2S)-H(2S2)	108(3)
H(2S1)-C(2S)-H(2S2)	104(4)
C(1S)-C(2S)-H(2S3)	112(3)
H(2S1)-C(2S)-H(2S3)	105(4)
H(2S2)-C(2S)-H(2S3)	115(4)
C(1S)-C(2S)-H(2S4)	108(3)
H(2S1)-C(2S)-H(2S4)	58(4)
H(2S2)-C(2S)-H(2S4)	50(3)
H(2S3)-C(2S)-H(2S4)	140(4)
C(1S)-C(2S)-H(2S5)	106(3)
H(2S1)-C(2S)-H(2S5)	51(3)
H(2S2)-C(2S)-H(2S5)	143(4)
H(2S3)-C(2S)-H(2S5)	60(4)
H(2S4)-C(2S)-H(2S5)	108(4)
C(1S)-C(2S)-H(2S6)	113(3)
H(2S1)-C(2S)-H(2S6)	134(4)
H(2S2)-C(2S)-H(2S6)	66(4)

H(2S3)-C(2S)-H(2S6)	53(3)
H(2S4)-C(2S)-H(2S6)	111(5)
H(2S5)-C(2S)-H(2S6)	111(4)
N(2S)-C(3S)-C(4S)	178.1(5)

Symmetry transformations used to generate equivalent atoms:

Table S4. Anisotropic displacement parameters ($Å^2 x 10^3$)for raj0b. The anisotropic displacement factor exponent takes the form: $-2\pi^2[h^2a^{*2}U^{11} + ... + 2 h k a^* b^* U^{12}]$

	U^{11}	U^{22}	U^{33}	U^{23}	U^{13}	U^{12}
C(10)	25(1)	22(1)	26(1)	5(1)	7(1)	3(1)
C(11)	23(1)	25(1)	28(1)	9(1)	9(1)	1(1)
C(12)	23(1)	22(1)	29(1)	7(1)	10(1)	1(1)
C(13)	25(1)	28(1)	24(1)	5(1)	8(1)	2(1)
C(14)	24(1)	28(1)	30(1)	8(1)	10(1)	4(1)
C(15)	26(1)	24(1)	29(1)	5(1)	11(1)	3(1)
C(16)	24(1)	23(1)	27(1)	7(1)	10(1)	0(1)
O(1)	35(1)	34(1)	27(1)	10(1)	8(1)	13(1)
C(17)	45(2)	20(1)	26(1)	10(1)	5(1)	8(1)
C(18)	52(2)	38(2)	34(1)	8(1)	15(1)	2(1)
C(19)	48(2)	33(1)	43(2)	16(1)	19(1)	5(1)
O(1A)	35(1)	34(1)	27(1)	10(1)	8(1)	13(1)
C(20)	28(1)	26(1)	26(1)	7(1)	11(1)	4(1)
C(21)	29(1)	24(1)	19(1)	5(1)	6(1)	6(1)
C(22)	27(1)	25(1)	18(1)	3(1)	9(1)	3(1)
C(23)	23(1)	26(1)	24(1)	2(1)	8(1)	6(1)
C(24)	26(1)	23(1)	22(1)	2(1)	8(1)	5(1)
C(25)	23(1)	23(1)	21(1)	3(1)	6(1)	3(1)
C(26)	25(1)	25(1)	17(1)	4(1)	5(1)	6(1)
O(2)	33(1)	28(1)	34(1)	14(1)	8(1)	8(1)
C(27)	55(1)	53(1)	38(1)	24(1)	15(1)	18(1)
C(28)	87(3)	83(2)	51(3)	38(2)	39(2)	53(2)
C(29)	110(3)	116(3)	65(2)	62(2)	34(2)	62(3)
C(27A)	55(1)	53(1)	38(1)	24(1)	15(1)	18(1)
C(28A)	87(3)	83(2)	51(3)	38(2)	39(2)	53(2)
C(30)	23(1)	24(1)	22(1)	6(1)	4(1)	5(1)
C(31)	18(1)	23(1)	27(1)	9(1)	4(1)	5(1)
C(32)	18(1)	26(1)	23(1)	7(1)	3(1)	5(1)
C(33)	21(1)	23(1)	27(1)	5(1)	5(1)	5(1)
C(34)	21(1)	26(1)	27(1)	9(1)	7(1)	5(1)
C(35)	22(1)	28(1)	23(1)	7(1)	7(1)	5(1)

C(36)	18(1)	25(1)	27(1)	7(1)	6(1)	7(1)
O(3)	35(1)	24(1)	26(1)	11(1)	7(1)	7(1)
C(37)	44(1)	41(2)	37(1)	22(1)	11(1)	22(2)
C(38)	35(1)	31(2)	41(1)	10(1)	6(1)	13(1)
C(39)	35(1)	47(2)	56(2)	22(1)	9(1)	17(1)
O(3A)	35(1)	24(1)	26(1)	11(1)	7(1)	7(1)
C(37A)	44(1)	41(2)	37(1)	22(1)	11(1)	22(2)
O(4)	31(1)	41(1)	29(1)	19(1)	6(1)	6(1)
O(4A)	31(1)	41(1)	29(1)	19(1)	6(1)	6(1)
C(51)	27(1)	30(1)	26(1)	9(1)	12(1)	9(1)
C(52)	29(1)	31(1)	26(1)	7(1)	11(1)	10(1)
C(53)	36(1)	32(1)	31(1)	8(1)	15(1)	16(1)
C(54)	34(1)	40(1)	28(1)	10(1)	14(1)	20(1)
C(55)	30(1)	39(1)	26(1)	5(1)	9(1)	14(1)
C(56)	30(1)	29(1)	30(1)	7(1)	11(1)	11(1)
O(5)	39(1)	27(1)	32(1)	8(1)	6(1)	7(1)
O(6)	40(1)	44(1)	34(1)	0(1)	-2(1)	19(1)
C(57)	41(1)	29(1)	40(1)	10(1)	13(1)	10(1)
C(58)	51(1)	51(1)	35(1)	12(1)	10(1)	31(1)
C(59)	51(1)	47(2)	46(1)	-2(1)	-8(1)	15(1)
C(51A)	27(1)	30(1)	26(1)	9(1)	12(1)	9(1)
C(52A)	29(1)	31(1)	26(1)	7(1)	11(1)	10(1)
C(53A)	36(1)	32(1)	31(1)	8(1)	15(1)	16(1)
C(54A)	34(1)	40(1)	28(1)	10(1)	14(1)	20(1)
C(55A)	30(1)	39(1)	26(1)	5(1)	9(1)	14(1)
C(56A)	30(1)	29(1)	30(1)	7(1)	11(1)	11(1)
O(5A)	39(1)	27(1)	32(1)	8(1)	6(1)	7(1)
O(6A)	40(1)	44(1)	34(1)	0(1)	-2(1)	19(1)
C(57A)	41(1)	29(1)	40(1)	10(1)	13(1)	10(1)
C(58A)	51(1)	51(1)	35(1)	12(1)	10(1)	31(1)
C(59A)	51(1)	47(2)	46(1)	-2(1)	-8(1)	15(1)
C(61)	20(1)	22(1)	27(1)	2(1)	3(1)	2(1)
C(62)	19(1)	26(1)	33(1)	2(1)	5(1)	2(1)
C(63)	22(1)	26(1)	38(1)	-1(1)	3(1)	7(1)
C(64)	29(1)	24(1)	34(1)	3(1)	-4(1)	8(1)
C(65)	34(1)	28(1)	28(1)	7(1)	3(1)	7(1)

C(66)	27(1)	24(1)	28(1)	2(1)	5(1)	6(1)
O(7)	34(1)	28(1)	43(1)	4(1)	22(1)	8(1)
O(8)	60(1)	33(1)	37(1)	15(1)	16(1)	16(1)
C(67)	37(1)	38(1)	48(1)	4(1)	22(1)	14(1)
C(68)	54(1)	33(1)	43(1)	9(1)	2(1)	18(1)
C(69)	63(1)	44(1)	43(1)	20(1)	25(1)	16(1)
C(71)	26(1)	23(2)	25(1)	7(1)	11(1)	7(1)
C(72)	26(2)	26(2)	26(1)	5(1)	6(1)	2(1)
C(73)	32(2)	36(2)	25(1)	13(2)	5(1)	6(1)
C(74)	30(2)	32(1)	32(2)	14(1)	7(1)	2(1)
C(75)	29(1)	26(1)	26(1)	7(1)	8(1)	-1(1)
C(76)	26(1)	27(2)	24(1)	10(1)	7(1)	6(1)
O(9)	31(1)	29(1)	30(1)	9(1)	-1(1)	-1(1)
O(10)	38(1)	30(1)	31(1)	12(1)	1(1)	-6(1)
C(77)	33(1)	37(1)	31(1)	9(1)	0(1)	1(1)
C(78)	47(2)	40(1)	35(2)	19(1)	-3(1)	-6(1)
C(79)	37(1)	28(1)	31(1)	6(1)	1(1)	0(1)
C(71A)	26(1)	23(2)	25(1)	7(1)	11(1)	7(1)
C(72A)	26(2)	26(2)	26(1)	5(1)	6(1)	2(1)
C(73A)	32(2)	36(2)	25(1)	13(2)	5(1)	6(1)
C(74A)	30(2)	32(1)	32(2)	14(1)	7(1)	2(1)
C(75A)	29(1)	26(1)	26(1)	7(1)	8(1)	-1(1)
C(76A)	26(1)	27(2)	24(1)	10(1)	7(1)	6(1)
O(9A)	31(1)	29(1)	30(1)	9(1)	-1(1)	-1(1)
O(10A)	38(1)	30(1)	31(1)	12(1)	1(1)	-6(1)
C(77A)	33(1)	37(1)	31(1)	9(1)	0(1)	1(1)
C(78A)	47(2)	40(1)	35(2)	19(1)	-3(1)	-6(1)
C(79A)	37(1)	28(1)	31(1)	6(1)	1(1)	0(1)
C(81)	22(2)	24(2)	25(1)	9(1)	4(1)	0(2)
C(82)	22(2)	32(2)	28(1)	10(1)	5(1)	4(1)
C(83)	30(1)	36(1)	28(1)	13(1)	10(1)	2(1)
C(84)	29(1)	31(1)	30(1)	14(1)	6(1)	3(1)
C(85)	27(1)	30(1)	32(1)	13(1)	5(1)	5(1)
C(86)	29(1)	28(1)	26(2)	12(1)	7(1)	3(1)
O(11)	35(1)	47(1)	30(1)	18(1)	15(1)	18(1)
O(12)	41(1)	47(1)	44(1)	24(1)	14(1)	22(1)

C(87)	49(1)	71(2)	39(1)	26(1)	23(1)	29(1)
C(88)	42(1)	57(2)	42(1)	29(1)	13(1)	15(1)
C(89)	64(2)	72(2)	56(2)	37(2)	32(1)	45(2)
C(81A)	22(2)	24(2)	25(1)	9(1)	4(1)	0(2)
C(82A)	22(2)	32(2)	28(1)	10(1)	5(1)	4(1)
C(83A)	30(1)	36(1)	28(1)	13(1)	10(1)	2(1)
C(84A)	29(1)	31(1)	30(1)	14(1)	6(1)	3(1)
C(85A)	27(1)	30(1)	32(1)	13(1)	5(1)	5(1)
C(86A)	29(1)	28(1)	26(2)	12(1)	7(1)	3(1)
O(11A)	35(1)	47(1)	30(1)	18(1)	15(1)	18(1)
O(12A)	41(1)	47(1)	44(1)	24(1)	14(1)	22(1)
C(87A)	49(1)	71(2)	39(1)	26(1)	23(1)	29(1)
C(88A)	42(1)	57(2)	42(1)	29(1)	13(1)	15(1)
C(89A)	64(2)	72(2)	56(2)	37(2)	32(1)	45(2)
N(1S)	70(1)	55(1)	57(1)	24(1)	27(1)	28(1)
C(1S)	38(1)	32(1)	34(1)	4(1)	14(1)	8(1)
C(2S)	33(1)	30(1)	34(1)	5(1)	12(1)	6(1)
N(2S)	106(3)	93(3)	122(3)	39(2)	12(2)	5(2)
C(3S)	103(3)	62(2)	92(3)	33(2)	7(2)	11(2)
C(4S)	86(2)	60(2)	99(3)	41(2)	0(2)	15(2)

Table S5. Hydrogen coordinates (x 10^4) and isotropic displacement parameters ($Å^2$x 10^3) for raj0b.

	x	y	z	U(eq)
H(10A)	14926	10351	3588	30
H(10B)	13949	10271	2809	30
H(13)	15777	9103	3680	32
H(15)	15675	7454	1699	31
H(17A)	13570	10017	711	36
H(17B)	14572	9521	764	36
H(18A)	15647	10509	1959	51
H(18B)	14650	11012	1885	51
H(19A)	16165	11647	1449	60
H(19B)	15015	11298	740	60
H(19C)	16009	10792	811	60
H(17C)	14821	10021	1161	91
H(17D)	14317	10572	1780	91
H(18C)	13013	9654	206	104
H(18D)	13400	10649	499	104
H(19D)	11401	10191	378	116
H(19E)	12074	10662	1266	116
H(19F)	11699	9664	993	116
H(20A)	14645	7615	428	32
H(20B)	13813	8252	406	32
H(23)	14029	6328	869	30
H(25)	10639	5471	593	28
H(27A)	10723	7277	-914	55
H(27B)	12053	7620	-863	55
H(28A)	11777	9018	-594	76
H(28B)	10433	8645	-706	76
H(29A)	10714	8873	-1921	127
H(29B)	10212	7901	-2036	127
H(29C)	11556	8246	-1926	127
H(27C)	10657	7333	-883	55

H(27D)	11988	7492	-846	55
H(28C)	11025	8226	-1616	76
H(28D)	12026	8860	-892	76
H(29D)	10242	9350	-914	230
H(29E)	10554	9018	-158	230
H(29F)	9573	8448	-918	230
H(30A)	9229	6293	-1	28
H(30B)	9746	7274	160	28
H(33)	9231	5715	1163	28
H(35)	9627	7301	3246	29
H(37A)	8344	7920	501	45
H(37B)	9005	8793	430	45
H(38A)	7922	8622	1638	43
H(38B)	8622	9498	1597	43
H(39A)	6644	9369	1045	66
H(39B)	6552	8493	456	66
H(39C)	7269	9336	370	66
H(37C)	8528	7917	390	45
H(37D)	8944	8899	571	45
H(38C)	7006	8357	641	54
H(38D)	7613	8205	1455	54
H(39D)	7174	9520	1663	83
H(39E)	7936	9781	1118	83
H(39F)	8543	9630	1930	83
H(40A)	9599	8794	3311	29
H(40B)	9920	9166	2637	29
H(43)	11149	8464	4262	29
H(45)	14483	9404	4428	30
H(47A)	10976	10605	2198	48
H(47B)	10921	10457	3018	48
H(48A)	12894	11379	2764	73
H(48B)	12813	11246	3587	73
H(49A)	12477	12574	3509	102
H(49B)	11328	12004	3561	102
H(49C)	11424	12144	2743	102
H(47C)	12285	10868	3042	42

H(47D)	10937	10513	2607	42
H(48C)	12711	10878	1913	47
H(48D)	11691	11347	1947	47
H(49D)	11354	10465	696	64
H(49E)	10352	10164	1066	64
H(49F)	11359	9675	1044	64
H(53)	17319	6122	3532	37
H(56)	17617	8983	3993	35
H(57A)	14852	5317	2006	54
H(57B)	15159	5603	2933	54
H(57C)	16126	5382	2523	54
H(58A)	18881	7093	5359	66
H(58B)	19792	7136	4863	66
H(58C)	18807	6304	4669	66
H(59A)	19524	9728	5805	81
H(59B)	18161	9351	5468	81
H(59C)	18875	9707	4936	81
H(53A)	18752	7831	4781	37
H(56A)	15584	6502	2591	35
H(57D)	18707	9902	4927	54
H(57E)	19081	9076	5080	54
H(57F)	17848	9224	5144	54
H(58D)	18157	5606	4285	66
H(58E)	17884	6130	5030	66
H(58F)	19082	6423	4850	66
H(59D)	15294	4454	2607	81
H(59E)	15648	5179	2213	81
H(59F)	14778	5266	2738	81
H(63)	13643	3371	286	37
H(66)	11783	4999	1739	33
H(67A)	14000	4435	-1075	60
H(67B)	13152	3674	-953	60
H(67C)	14455	3996	-414	60
H(68A)	13366	2677	1851	67
H(68B)	13508	2367	999	67
H(68C)	12255	2204	1134	67

H(69A)	11053	3767	2910	70
H(69B)	10605	4125	2186	70
H(69C)	11787	4611	2837	70
H(73)	10153	4898	4031	37
H(76)	7829	4977	1667	30
H(77A)	12161	6779	4605	54
H(77B)	11932	5794	4241	54
H(77C)	11150	6156	4756	54
H(78A)	9267	3484	3829	66
H(78B)	8434	2965	2990	66
H(78C)	7915	3451	3627	66
H(79A)	5872	3065	1106	52
H(79B)	6875	3699	960	52
H(79C)	6062	4049	1455	52
H(73A)	7573	3820	2453	37
H(76A)	10457	6223	3544	30
H(77D)	6135	4191	701	54
H(77E)	6218	3958	1507	54
H(77F)	7026	3656	986	54
H(78D)	8159	3258	3461	66
H(78E)	9070	3864	4237	66
H(78F)	9504	3281	3609	66
H(79D)	12041	5743	5173	52
H(79E)	11377	6297	4706	52
H(79F)	12137	5744	4320	52
H(83)	12973	8340	6794	37
H(86)	14427	8016	4655	33
H(87A)	11319	9768	6494	70
H(87B)	12478	9572	6953	70
H(87C)	11331	8842	6569	70
H(88A)	14136	7532	7357	66
H(88B)	15351	7604	7164	66
H(88C)	14328	6792	6725	66
H(89A)	16338	6781	5074	82
H(89B)	16192	7684	4992	82
H(89C)	15158	6880	4547	82

H(83A)	14883	7501	6029	37
H(86A)	12092	9069	5712	33
H(87D)	15420	7170	4193	70
H(87E)	14978	6881	4868	70
H(87F)	16033	7674	5081	70
H(88D)	13957	7221	7202	66
H(88E)	13910	8116	7693	66
H(88F)	15080	7974	7500	66
H(89D)	11307	9203	7390	82
H(89E)	11018	8836	6470	82
H(89F)	11938	9705	6900	82
H(2S1)	12060(50)	7020(30)	2120(30)	50
H(2S2)	13290(40)	7590(30)	2460(30)	50
H(2S3)	12250(50)	7620(30)	2920(30)	50
H(2S4)	12820(60)	7200(40)	2120(40)	50
H(2S5)	11810(50)	7130(40)	2490(40)	50
H(2S6)	12950(60)	7840(40)	2930(30)	50
H(4S1)	16059	5134	6472	125
H(4S2)	16019	6097	6614	125
H(4S3)	16235	5610	5829	125

Table S1. Crystal data and structure refinement for raj0ea.

Identification code	raj0ea	
Empirical formula	C77.75 H91.50 Cl15.50 O12 Sb2	
Formula weight	2010.98	
Temperature	100(2) K	
Wavelength	1.54178 Å	
Crystal system	Triclinic	
Space group	P -1	
Unit cell dimensions	a = 15.8677(5) Å	α= 109.7000(10)°.
	b = 17.4783(5) Å	β= 107.0050(10)°.
	c = 18.9410(5) Å	γ = 97.749(2)°.
Volume	4567.7(2) $Å^3$	
Z	2	
Density (calculated)	1.462 Mg/m^3	
Absorption coefficient	9.301 mm^{-1}	
F(000)	2039	
Crystal size	0.55 x 0.12 x 0.12 mm^3	
Theta range for data collection	2.66 to 66.97°.	
Index ranges	-18<=h<=17, -20<=k<=19, 0<=l<=22	
Reflections collected	37158	
Independent reflections	14945 [R(int) = 0.0286]	
Completeness to theta = 66.97°	91.9 %	
Absorption correction	Numerical	
Max. and min. transmission	0.4015 and 0.0798	
Refinement method	Full-matrix least-squares on F^2	
Data / restraints / parameters	14945 / 93 / 1061	
Goodness-of-fit on F^2	1.086	
Final R indices [I>2sigma(I)]	R1 = 0.1296, wR2 = 0.3220	
R indices (all data)	R1 = 0.1348, wR2 = 0.3237	
Largest diff. peak and hole	2.393 and -1.707 e.$Å^{-3}$	

Table S2. Atomic coordinates (x 10^4) and equivalent isotropic displacement parameters ($Å^2$x 10^3) for raj0ea. U(eq) is defined as one third of the trace of the orthogonalized U^{ij} tensor.

	x	y	z	U(eq)
Sb(1)	-711(1)	28(1)	-3211(1)	31(1)
Cl(1)	638(3)	782(3)	-3229(3)	68(1)
Cl(2)	-2066(3)	-751(3)	-3243(3)	61(1)
Cl(3)	-1266(3)	-595(2)	-4639(2)	43(1)
Cl(4)	-185(3)	661(3)	-1796(2)	54(1)
Cl(5)	-6(3)	-1058(2)	-3164(2)	49(1)
Cl(6)	-1409(3)	1136(2)	-3269(2)	57(1)
Sb(2)	-7565(2)	-4377(2)	-6682(1)	67(1)
Cl(7)	-9005(5)	-4726(6)	-7697(4)	53(2)
Cl(8)	-6157(9)	-4141(13)	-5695(8)	122(8)
Cl(9)	-8270(9)	-4945(7)	-5967(6)	101(5)
Cl(10)	-6857(7)	-3855(13)	-7410(6)	176(11)
Cl(11)	-7732(11)	-3055(6)	-6037(7)	114(5)
Cl(12)	-7435(15)	-5736(11)	-7401(9)	250(18)
Sb(3)	-6355(2)	-3493(1)	-6704(1)	46(1)
Cl(13)	-6066(9)	-3652(7)	-5489(5)	62(3)
Cl(14)	-6651(5)	-3286(5)	-7917(4)	45(2)
Cl(15)	-7338(7)	-2641(6)	-6364(6)	69(3)
Cl(16)	-5376(7)	-4300(7)	-7057(5)	74(3)
Cl(17)	-7588(7)	-4688(6)	-7357(6)	77(3)
Cl(18)	-5103(8)	-2284(6)	-6063(5)	78(3)
C(10)	2891(11)	-4613(9)	-3831(9)	42(4)
C(11)	1819(10)	-3764(8)	-3373(7)	36(3)
C(12)	2488(9)	-3845(8)	-3720(7)	31(3)
C(13)	2806(9)	-3202(8)	-3919(7)	30(3)
C(14)	2474(9)	-2498(8)	-3812(7)	30(3)
C(15)	1859(8)	-2383(8)	-3403(7)	25(3)
C(16)	1541(9)	-3004(9)	-3165(7)	32(3)
O(1)	1460(7)	-4388(6)	-3196(6)	38(2)
C(17)	609(15)	-4954(13)	-3869(10)	88(8)
C(18)	258(19)	-5612(15)	-3630(14)	108(10)

C(19)	-660(30)	-6190(30)	-4350(20)	270(40)
C(20)	952(9)	-2861(9)	-2661(8)	32(3)
C(21)	2217(9)	-2074(8)	-1318(8)	28(3)
C(22)	1468(9)	-2742(9)	-1816(8)	33(3)
C(23)	1257(8)	-3359(9)	-1545(8)	34(3)
C(24)	1774(9)	-3282(9)	-755(7)	29(3)
C(25)	2536(9)	-2599(8)	-294(8)	28(3)
C(26)	2785(9)	-2011(8)	-545(7)	29(3)
O(2)	2407(6)	-1399(6)	-1538(6)	37(2)
C(27)	1926(14)	-757(10)	-1275(10)	57(5)
C(28)	2172(16)	-29(12)	-1495(11)	73(6)
C(29)	3019(14)	587(11)	-864(13)	69(5)
C(30)	3688(8)	-1313(8)	-62(8)	27(3)
C(31)	4713(9)	-2201(8)	-539(8)	30(3)
C(32)	4388(8)	-1497(8)	-474(7)	27(3)
C(33)	4657(9)	-969(8)	-815(7)	27(3)
C(34)	5275(8)	-1136(8)	-1220(7)	26(3)
C(35)	5568(8)	-1874(9)	-1298(8)	33(3)
C(36)	5286(8)	-2408(8)	-993(8)	29(3)
O(3)	4476(6)	-2747(6)	-205(5)	30(2)
C(37)	5027(10)	-2483(9)	616(8)	37(3)
C(38)	4773(13)	-3149(11)	904(11)	53(4)
C(39)	5170(20)	-2877(14)	1790(12)	88(8)
C(40)	5508(9)	-3256(9)	-1183(9)	38(3)
C(41)	4261(10)	-4056(8)	-2533(8)	33(3)
C(42)	4691(10)	-3964(8)	-1751(8)	33(3)
C(43)	4299(10)	-4546(8)	-1495(8)	34(3)
C(44)	3502(10)	-5191(8)	-2012(8)	36(3)
C(45)	3070(11)	-5199(9)	-2788(9)	40(3)
C(46)	3406(10)	-4628(8)	-3050(8)	35(3)
O(4)	4668(7)	-3533(7)	-2822(6)	47(3)
C(47)	5160(20)	-3920(20)	-3320(20)	119(12)
C(48)	5640(20)	-3400(40)	-3530(20)	230(30)
C(49)	6270(20)	-2810(30)	-3250(30)	200(30)
C(51)	2798(9)	-1862(8)	-4081(7)	26(3)
C(52)	2194(9)	-1456(8)	-4456(7)	28(3)

C(53)	2507(10)	-856(9)	-4725(8)	35(3)
C(54)	3393(9)	-650(9)	-4653(8)	36(3)
C(55)	3992(9)	-1097(8)	-4339(7)	30(3)
C(56)	3698(9)	-1660(8)	-4029(8)	29(3)
O(5)	1323(6)	-1701(6)	-4547(5)	30(2)
O(6)	4842(6)	-882(6)	-4307(6)	38(2)
C(57)	665(10)	-1333(10)	-4947(10)	43(4)
C(58)	3761(11)	-2(10)	-4921(10)	49(4)
C(59)	5470(9)	-1325(11)	-4019(10)	43(4)
C(61)	1496(9)	-3942(9)	-479(8)	33(3)
C(62)	1517(9)	-3705(9)	342(8)	33(3)
C(63)	1304(10)	-4336(10)	613(9)	42(4)
C(64)	1043(10)	-5175(11)	96(10)	44(4)
C(65)	974(10)	-5407(10)	-714(10)	42(4)
C(66)	1204(9)	-4787(9)	-983(9)	38(3)
O(7)	1713(9)	-2888(7)	779(7)	59(3)
O(8)	674(7)	-6220(7)	-1161(8)	54(3)
C(67)	1490(13)	-2592(12)	1490(10)	63(5)
C(68)	870(11)	-5856(11)	394(11)	53(4)
C(69)	421(17)	-6480(14)	-2039(13)	97(9)
C(71)	5539(10)	-570(8)	-1599(8)	31(3)
C(72)	6439(10)	-295(9)	-1544(8)	36(3)
C(73)	6637(10)	238(9)	-1918(8)	39(3)
C(74)	5952(10)	518(9)	-2346(8)	36(3)
C(75)	5085(10)	273(8)	-2378(8)	34(3)
C(76)	4870(9)	-281(8)	-2017(7)	31(3)
O(9)	7085(7)	-562(7)	-1095(7)	50(3)
O(10)	4441(7)	558(7)	-2812(6)	44(2)
C(77)	3596(11)	522(10)	-2696(10)	45(4)
C(78)	6191(12)	1052(11)	-2788(11)	56(5)
C(79)	8011(12)	-205(17)	-958(14)	81(7)
C(81)	3133(10)	-5801(9)	-1735(9)	37(3)
C(82)	2715(12)	-6662(10)	-2263(9)	49(4)
C(83)	2379(13)	-7211(10)	-1956(12)	60(5)
C(84)	2436(11)	-6990(10)	-1199(10)	44(4)
C(85)	2849(9)	-6133(10)	-673(9)	40(3)

C(86)	3203(10)	-5559(9)	-933(8)	36(3)
O(11)	2708(10)	-6877(7)	-3004(7)	64(4)
O(12)	2874(7)	-5930(6)	118(6)	44(2)
C(87)	2216(15)	-7714(10)	-3584(10)	67(6)
C(88)	2069(12)	-7582(10)	-852(10)	51(4)
C(89)	3363(10)	-5100(9)	704(9)	40(3)
Cl(1S)	-5620(9)	-7686(7)	-3917(6)	76(3)
Cl(2S)	-6273(9)	-6493(8)	-4558(6)	86(3)
C(1S)	-5590(20)	-6523(12)	-3636(13)	52(8)
Cl(3S)	-8949(12)	-6820(13)	-5257(13)	44(6)
Cl(4S)	-7037(10)	-6750(9)	-4846(13)	69(6)
C(2S)	-8022(18)	-6970(40)	-5713(16)	44(14)
Cl(3A)	-9047(12)	-6793(11)	-5437(12)	36(5)
Cl(4A)	-8094(12)	-6038(10)	-6198(13)	66(5)
C(2SA)	-8360(40)	-7027(18)	-6130(30)	38(13)
Cl(5S)	-808(11)	-2003(10)	-1894(9)	112(4)
Cl(6S)	-335(16)	-1764(10)	-148(7)	146(8)
C(3S)	-500(14)	-2456(11)	-1113(9)	78(12)
Cl(5A)	-532(14)	-1776(11)	-1634(9)	112(4)
Cl(6A)	-1863(14)	-2623(11)	-1117(9)	117(9)
C(3SA)	-1075(14)	-2903(11)	-1648(9)	56(17)

Table S3. Bond lengths [Å] and angles [°] for raj0ea.

Sb(1)-Cl(5)	2.343(4)
Sb(1)-Cl(4)	2.356(4)
Sb(1)-Cl(2)	2.358(4)
Sb(1)-Cl(1)	2.373(4)
Sb(1)-Cl(6)	2.375(4)
Sb(1)-Cl(3)	2.375(3)
Sb(2)-Cl(11)	2.313(10)
Sb(2)-Cl(8)	2.333(11)
Sb(2)-Cl(10)	2.340(10)
Sb(2)-Cl(7)	2.355(7)
Sb(2)-Cl(9)	2.358(9)
Sb(2)-Cl(12)	2.377(11)
Sb(3)-Cl(16)	2.327(8)
Sb(3)-Cl(13)	2.333(9)
Sb(3)-Cl(17)	2.337(8)
Sb(3)-Cl(14)	2.366(7)
Sb(3)-Cl(18)	2.370(8)
Sb(3)-Cl(15)	2.373(8)
C(10)-C(46)	1.48(2)
C(10)-C(12)	1.541(19)
C(11)-O(1)	1.349(16)
C(11)-C(12)	1.40(2)
C(11)-C(16)	1.42(2)
C(12)-C(13)	1.380(18)
C(13)-C(14)	1.379(18)
C(14)-C(15)	1.408(18)
C(14)-C(51)	1.462(17)
C(15)-C(16)	1.396(18)
C(16)-C(20)	1.508(19)
O(1)-C(17)	1.485(18)
C(17)-C(18)	1.47(3)
C(18)-C(19)	1.59(4)
C(20)-C(22)	1.496(18)
C(21)-C(22)	1.376(19)

C(21)-O(2)	1.403(15)
C(21)-C(26)	1.434(18)
C(22)-C(23)	1.385(19)
C(23)-C(24)	1.430(18)
C(24)-C(25)	1.393(18)
C(24)-C(61)	1.483(18)
C(25)-C(26)	1.330(18)
C(26)-C(30)	1.545(17)
O(2)-C(27)	1.47(2)
C(27)-C(28)	1.50(2)
C(28)-C(29)	1.48(3)
C(30)-C(32)	1.543(17)
C(31)-O(3)	1.378(15)
C(31)-C(32)	1.379(18)
C(31)-C(36)	1.427(18)
C(32)-C(33)	1.382(18)
C(33)-C(34)	1.414(17)
C(34)-C(35)	1.406(18)
C(34)-C(71)	1.488(17)
C(35)-C(36)	1.343(19)
C(36)-C(40)	1.519(18)
O(3)-C(37)	1.417(15)
C(37)-C(38)	1.50(2)
C(38)-C(39)	1.48(3)
C(40)-C(42)	1.492(19)
C(41)-C(42)	1.38(2)
C(41)-O(4)	1.391(16)
C(41)-C(46)	1.41(2)
C(42)-C(43)	1.411(19)
C(43)-C(44)	1.41(2)
C(44)-C(45)	1.42(2)
C(44)-C(81)	1.464(19)
C(45)-C(46)	1.37(2)
O(4)-C(47)	1.45(3)
C(47)-C(48)	1.35(4)
C(48)-C(49)	1.18(5)

C(51)-C(56)	1.390(18)
C(51)-C(52)	1.433(18)
C(52)-O(5)	1.331(15)
C(52)-C(53)	1.406(18)
C(53)-C(54)	1.36(2)
C(54)-C(55)	1.43(2)
C(54)-C(58)	1.503(19)
C(55)-O(6)	1.330(16)
C(55)-C(56)	1.399(18)
O(5)-C(57)	1.457(17)
O(6)-C(59)	1.438(17)
C(61)-C(66)	1.38(2)
C(61)-C(62)	1.458(18)
C(62)-O(7)	1.324(18)
C(62)-C(63)	1.404(19)
C(63)-C(64)	1.38(2)
C(64)-C(65)	1.42(2)
C(64)-C(68)	1.51(2)
C(65)-O(8)	1.317(19)
C(65)-C(66)	1.39(2)
O(7)-C(67)	1.436(18)
O(8)-C(69)	1.48(2)
C(71)-C(76)	1.39(2)
C(71)-C(72)	1.405(19)
C(72)-O(9)	1.370(18)
C(72)-C(73)	1.402(19)
C(73)-C(74)	1.40(2)
C(74)-C(75)	1.36(2)
C(74)-C(78)	1.527(19)
C(75)-O(10)	1.375(17)
C(75)-C(76)	1.420(18)
O(9)-C(79)	1.426(19)
O(10)-C(77)	1.416(19)
C(81)-C(86)	1.40(2)
C(81)-C(82)	1.43(2)
C(82)-O(11)	1.321(19)

C(82)-C(83)	1.40(2)
C(83)-C(84)	1.32(2)
C(84)-C(85)	1.42(2)
C(84)-C(88)	1.54(2)
C(85)-C(86)	1.38(2)
C(85)-O(12)	1.405(18)
O(11)-C(87)	1.436(19)
O(12)-C(89)	1.431(18)
Cl(1S)-C(1S)	1.909(17)
Cl(2S)-C(1S)	1.788(17)
Cl(3S)-C(2S)	1.915(18)
Cl(4S)-C(2S)	1.788(19)
Cl(3A)-C(2SA)	1.914(18)
Cl(4A)-C(2SA)	1.779(18)
Cl(5S)-C(3S)	1.877(16)
Cl(6S)-C(3S)	1.737(15)
Cl(5A)-C(3SA)	2.0288
Cl(6A)-C(3SA)	1.8386

Cl(5)-Sb(1)-Cl(4)	91.03(15)
Cl(5)-Sb(1)-Cl(2)	90.96(16)
Cl(4)-Sb(1)-Cl(2)	90.37(16)
Cl(5)-Sb(1)-Cl(1)	88.65(17)
Cl(4)-Sb(1)-Cl(1)	91.67(16)
Cl(2)-Sb(1)-Cl(1)	177.93(17)
Cl(5)-Sb(1)-Cl(6)	179.04(16)
Cl(4)-Sb(1)-Cl(6)	89.41(15)
Cl(2)-Sb(1)-Cl(6)	89.89(17)
Cl(1)-Sb(1)-Cl(6)	90.48(18)
Cl(5)-Sb(1)-Cl(3)	90.16(13)
Cl(4)-Sb(1)-Cl(3)	178.72(15)
Cl(2)-Sb(1)-Cl(3)	89.14(15)
Cl(1)-Sb(1)-Cl(3)	88.83(15)
Cl(6)-Sb(1)-Cl(3)	89.41(12)
Cl(11)-Sb(2)-Cl(8)	93.3(6)
Cl(11)-Sb(2)-Cl(10)	90.0(6)

Cl(8)-Sb(2)-Cl(10)	91.3(5)
Cl(11)-Sb(2)-Cl(7)	90.5(4)
Cl(8)-Sb(2)-Cl(7)	175.6(6)
Cl(10)-Sb(2)-Cl(7)	90.9(3)
Cl(11)-Sb(2)-Cl(9)	91.8(5)
Cl(8)-Sb(2)-Cl(9)	88.5(5)
Cl(10)-Sb(2)-Cl(9)	178.2(6)
Cl(7)-Sb(2)-Cl(9)	89.3(3)
Cl(11)-Sb(2)-Cl(12)	177.4(5)
Cl(8)-Sb(2)-Cl(12)	88.9(6)
Cl(10)-Sb(2)-Cl(12)	88.7(7)
Cl(7)-Sb(2)-Cl(12)	87.3(5)
Cl(9)-Sb(2)-Cl(12)	89.5(7)
Cl(16)-Sb(3)-Cl(13)	92.2(4)
Cl(16)-Sb(3)-Cl(17)	90.8(4)
Cl(13)-Sb(3)-Cl(17)	91.2(4)
Cl(16)-Sb(3)-Cl(14)	89.3(3)
Cl(13)-Sb(3)-Cl(14)	178.2(3)
Cl(17)-Sb(3)-Cl(14)	89.8(3)
Cl(16)-Sb(3)-Cl(18)	88.8(5)
Cl(13)-Sb(3)-Cl(18)	89.7(4)
Cl(17)-Sb(3)-Cl(18)	179.0(4)
Cl(14)-Sb(3)-Cl(18)	89.3(3)
Cl(16)-Sb(3)-Cl(15)	178.2(4)
Cl(13)-Sb(3)-Cl(15)	89.3(4)
Cl(17)-Sb(3)-Cl(15)	90.2(4)
Cl(14)-Sb(3)-Cl(15)	89.2(3)
Cl(18)-Sb(3)-Cl(15)	90.2(4)
C(46)-C(10)-C(12)	111.6(12)
O(1)-C(11)-C(12)	120.7(13)
O(1)-C(11)-C(16)	118.8(13)
C(12)-C(11)-C(16)	120.4(12)
C(13)-C(12)-C(11)	118.3(12)
C(13)-C(12)-C(10)	121.2(13)
C(11)-C(12)-C(10)	120.4(12)
C(14)-C(13)-C(12)	122.7(13)

C(13)-C(14)-C(15)	118.9(12)
C(13)-C(14)-C(51)	120.1(12)
C(15)-C(14)-C(51)	120.9(12)
C(16)-C(15)-C(14)	120.2(12)
C(15)-C(16)-C(11)	118.8(12)
C(15)-C(16)-C(20)	121.0(13)
C(11)-C(16)-C(20)	120.1(12)
C(11)-O(1)-C(17)	110.8(11)
C(18)-C(17)-O(1)	107.5(16)
C(17)-C(18)-C(19)	107(2)
C(22)-C(20)-C(16)	111.3(11)
C(22)-C(21)-O(2)	119.7(12)
C(22)-C(21)-C(26)	122.3(12)
O(2)-C(21)-C(26)	117.9(11)
C(21)-C(22)-C(23)	117.9(12)
C(21)-C(22)-C(20)	121.9(12)
C(23)-C(22)-C(20)	119.9(12)
C(22)-C(23)-C(24)	120.9(12)
C(25)-C(24)-C(23)	117.7(12)
C(25)-C(24)-C(61)	123.4(12)
C(23)-C(24)-C(61)	118.9(12)
C(26)-C(25)-C(24)	123.2(13)
C(25)-C(26)-C(21)	117.9(12)
C(25)-C(26)-C(30)	122.1(12)
C(21)-C(26)-C(30)	119.9(11)
C(21)-O(2)-C(27)	112.3(10)
O(2)-C(27)-C(28)	110.9(16)
C(29)-C(28)-C(27)	110.3(16)
C(32)-C(30)-C(26)	110.0(10)
O(3)-C(31)-C(32)	122.5(12)
O(3)-C(31)-C(36)	117.4(11)
C(32)-C(31)-C(36)	120.1(12)
C(31)-C(32)-C(33)	119.8(12)
C(31)-C(32)-C(30)	120.9(11)
C(33)-C(32)-C(30)	119.2(11)
C(32)-C(33)-C(34)	120.6(12)

C(35)-C(34)-C(33)	118.1(11)
C(35)-C(34)-C(71)	122.0(11)
C(33)-C(34)-C(71)	119.7(11)
C(36)-C(35)-C(34)	121.9(12)
C(35)-C(36)-C(31)	119.3(12)
C(35)-C(36)-C(40)	120.8(12)
C(31)-C(36)-C(40)	119.7(12)
C(31)-O(3)-C(37)	113.0(10)
O(3)-C(37)-C(38)	109.0(12)
C(39)-C(38)-C(37)	113.9(16)
C(42)-C(40)-C(36)	112.0(11)
C(42)-C(41)-O(4)	118.6(12)
C(42)-C(41)-C(46)	123.6(12)
O(4)-C(41)-C(46)	117.8(12)
C(41)-C(42)-C(43)	117.3(13)
C(41)-C(42)-C(40)	121.7(12)
C(43)-C(42)-C(40)	120.9(13)
C(44)-C(43)-C(42)	121.6(13)
C(43)-C(44)-C(45)	117.1(13)
C(43)-C(44)-C(81)	120.1(13)
C(45)-C(44)-C(81)	122.7(13)
C(46)-C(45)-C(44)	123.2(14)
C(45)-C(46)-C(41)	116.5(13)
C(45)-C(46)-C(10)	121.3(14)
C(41)-C(46)-C(10)	122.2(13)
C(41)-O(4)-C(47)	114.2(14)
C(48)-C(47)-O(4)	115(3)
C(49)-C(48)-C(47)	141(4)
C(56)-C(51)-C(52)	116.6(11)
C(56)-C(51)-C(14)	121.5(12)
C(52)-C(51)-C(14)	121.8(12)
O(5)-C(52)-C(53)	122.9(12)
O(5)-C(52)-C(51)	115.6(11)
C(53)-C(52)-C(51)	121.5(12)
C(54)-C(53)-C(52)	121.2(13)
C(53)-C(54)-C(55)	118.2(13)

C(53)-C(54)-C(58)	123.0(14)
C(55)-C(54)-C(58)	118.8(13)
O(6)-C(55)-C(56)	124.0(12)
O(6)-C(55)-C(54)	115.1(12)
C(56)-C(55)-C(54)	120.7(12)
C(51)-C(56)-C(55)	121.5(12)
C(52)-O(5)-C(57)	118.5(10)
C(55)-O(6)-C(59)	117.7(10)
C(66)-C(61)-C(62)	118.2(12)
C(66)-C(61)-C(24)	121.9(12)
C(62)-C(61)-C(24)	119.9(12)
O(7)-C(62)-C(63)	124.3(13)
O(7)-C(62)-C(61)	116.2(11)
C(63)-C(62)-C(61)	119.5(13)
C(64)-C(63)-C(62)	120.4(14)
C(63)-C(64)-C(65)	120.3(14)
C(63)-C(64)-C(68)	120.7(15)
C(65)-C(64)-C(68)	118.9(16)
O(8)-C(65)-C(66)	125.2(15)
O(8)-C(65)-C(64)	115.1(14)
C(66)-C(65)-C(64)	119.7(15)
C(61)-C(66)-C(65)	121.7(14)
C(62)-O(7)-C(67)	120.3(12)
C(65)-O(8)-C(69)	116.9(14)
C(76)-C(71)-C(72)	117.8(12)
C(76)-C(71)-C(34)	118.8(12)
C(72)-C(71)-C(34)	123.4(12)
O(9)-C(72)-C(73)	123.8(13)
O(9)-C(72)-C(71)	116.0(12)
C(73)-C(72)-C(71)	120.2(13)
C(72)-C(73)-C(74)	121.4(13)
C(75)-C(74)-C(73)	118.4(12)
C(75)-C(74)-C(78)	122.1(14)
C(73)-C(74)-C(78)	119.4(13)
C(74)-C(75)-O(10)	116.0(12)
C(74)-C(75)-C(76)	120.9(14)

O(10)-C(75)-C(76)	123.1(13)
C(71)-C(76)-C(75)	121.3(13)
C(72)-O(9)-C(79)	116.1(13)
C(75)-O(10)-C(77)	118.5(11)
C(86)-C(81)-C(82)	117.7(13)
C(86)-C(81)-C(44)	120.7(12)
C(82)-C(81)-C(44)	121.6(13)
O(11)-C(82)-C(83)	125.5(15)
O(11)-C(82)-C(81)	116.0(13)
C(83)-C(82)-C(81)	118.5(15)
C(84)-C(83)-C(82)	124.5(16)
C(83)-C(84)-C(85)	117.2(14)
C(83)-C(84)-C(88)	125.2(14)
C(85)-C(84)-C(88)	117.5(14)
C(86)-C(85)-O(12)	123.9(14)
C(86)-C(85)-C(84)	121.3(14)
O(12)-C(85)-C(84)	114.8(12)
C(85)-C(86)-C(81)	120.7(13)
C(82)-O(11)-C(87)	118.4(13)
C(85)-O(12)-C(89)	118.0(11)
Cl(2S)-C(1S)-Cl(1S)	103.6(11)
Cl(4S)-C(2S)-Cl(3S)	101.9(14)
Cl(4A)-C(2SA)-Cl(3A)	100.9(13)
Cl(6S)-C(3S)-Cl(5S)	114.1(12)
Cl(6A)-C(3SA)-Cl(5A)	101.2

Symmetry transformations used to generate equivalent atoms:

Table S4. Anisotropic displacement parameters ($Å^2$x 10^3)for raj0ea. The anisotropic displacement factor exponent takes the form: $-2\pi^2[h^2a^{*2}U^{11} + ... + 2 h k a^* b^* U^{12}]$

	U^{11}	U^{22}	U^{33}	U^{23}	U^{13}	U^{12}
Sb(1)	31(1)	30(1)	27(1)	12(1)	8(1)	5(1)
Cl(1)	69(3)	54(2)	66(3)	13(2)	32(2)	-16(2)
Cl(2)	34(2)	73(3)	71(3)	28(2)	18(2)	3(2)
Cl(3)	60(2)	32(2)	28(2)	6(1)	10(2)	21(2)
Cl(4)	47(2)	77(3)	31(2)	18(2)	11(2)	11(2)
Cl(5)	50(2)	55(2)	59(2)	36(2)	22(2)	26(2)
Cl(6)	91(3)	45(2)	35(2)	10(2)	22(2)	36(2)
Sb(2)	53(1)	121(2)	31(1)	30(1)	14(1)	40(2)
Cl(7)	48(4)	74(5)	32(4)	22(4)	11(3)	11(4)
Cl(8)	72(9)	230(20)	50(8)	52(12)	5(6)	46(14)
Cl(9)	138(11)	91(8)	45(5)	43(6)	-3(6)	-20(7)
Cl(10)	45(6)	410(30)	46(6)	96(12)	5(5)	-11(10)
Cl(11)	192(15)	53(6)	72(7)	12(5)	46(9)	-9(8)
Cl(12)	320(30)	310(30)	95(11)	21(15)	22(15)	290(30)
Sb(3)	62(1)	48(1)	36(1)	17(1)	22(1)	22(1)
Cl(13)	89(8)	80(7)	26(5)	22(5)	29(5)	24(6)
Cl(14)	54(4)	42(4)	41(4)	17(3)	16(3)	17(3)
Cl(15)	101(8)	73(6)	62(5)	32(5)	50(6)	52(6)
Cl(16)	104(8)	99(7)	53(5)	42(5)	44(5)	71(7)
Cl(17)	89(7)	59(6)	79(7)	31(5)	31(6)	-4(5)
Cl(18)	90(8)	68(6)	41(5)	24(4)	-9(5)	-19(6)
C(10)	53(9)	36(8)	38(8)	14(7)	18(7)	10(7)
C(11)	42(8)	29(7)	14(6)	4(5)	-7(5)	-19(6)
C(12)	40(8)	18(6)	17(6)	-4(5)	3(5)	-2(5)
C(13)	30(7)	31(7)	25(6)	10(5)	5(5)	7(6)
C(14)	31(7)	26(7)	24(6)	9(5)	4(5)	-1(5)
C(15)	25(6)	27(6)	19(6)	7(5)	2(5)	8(5)
C(16)	25(7)	40(8)	19(6)	13(6)	-3(5)	-2(6)
O(1)	42(6)	27(5)	35(5)	7(4)	12(4)	-5(4)
C(17)	92(15)	79(14)	31(9)	3(9)	-2(9)	-69(12)
C(18)	120(20)	85(17)	62(14)	1(12)	31(14)	-57(15)

C(19)	310(60)	240(50)	90(20)	70(30)	-60(30)	-220(50)
C(20)	27(7)	36(7)	34(7)	21(6)	8(6)	0(6)
C(21)	27(7)	35(7)	30(7)	18(6)	15(6)	9(6)
C(22)	25(7)	50(8)	28(7)	19(6)	12(6)	5(6)
C(23)	17(6)	47(8)	38(7)	16(7)	14(6)	2(6)
C(24)	26(7)	41(8)	26(6)	16(6)	14(5)	9(6)
C(25)	28(7)	28(7)	27(7)	7(5)	13(5)	6(5)
C(26)	28(7)	36(7)	23(6)	6(6)	16(5)	9(6)
O(2)	38(5)	43(6)	40(5)	28(5)	15(4)	7(4)
C(27)	94(14)	34(8)	43(9)	19(7)	20(9)	18(9)
C(28)	100(16)	54(11)	40(10)	21(9)	-6(10)	-1(11)
C(29)	76(14)	40(10)	84(14)	22(10)	25(11)	10(9)
C(30)	29(7)	26(6)	28(7)	10(5)	13(5)	7(5)
C(31)	26(7)	23(6)	31(7)	7(5)	8(5)	-4(5)
C(32)	22(6)	24(6)	26(6)	4(5)	5(5)	1(5)
C(33)	28(7)	23(6)	25(6)	6(5)	7(5)	5(5)
C(34)	26(6)	25(6)	22(6)	6(5)	7(5)	3(5)
C(35)	18(6)	43(8)	38(7)	12(6)	14(6)	12(6)
C(36)	23(6)	29(7)	37(7)	14(6)	12(6)	7(5)
O(3)	29(5)	35(5)	28(5)	18(4)	9(4)	5(4)
C(37)	34(8)	43(8)	33(7)	20(6)	6(6)	4(6)
C(38)	66(11)	63(11)	63(11)	45(9)	40(9)	26(9)
C(39)	170(30)	73(14)	57(12)	36(11)	59(15)	61(16)
C(40)	24(7)	37(8)	51(9)	17(7)	8(6)	10(6)
C(41)	37(8)	29(7)	37(8)	14(6)	17(6)	11(6)
C(42)	37(8)	30(7)	39(8)	13(6)	22(6)	10(6)
C(43)	44(8)	33(7)	29(7)	9(6)	22(6)	14(6)
C(44)	47(8)	27(7)	30(7)	6(6)	19(6)	2(6)
C(45)	46(9)	28(7)	42(8)	12(6)	15(7)	7(6)
C(46)	48(9)	26(7)	33(7)	8(6)	19(7)	11(6)
O(4)	52(7)	46(6)	41(6)	25(5)	11(5)	-2(5)
C(47)	130(20)	160(30)	190(30)	130(30)	140(20)	80(20)
C(48)	80(20)	470(80)	100(20)	150(40)	-4(17)	-100(30)
C(49)	100(30)	260(50)	330(60)	260(50)	40(30)	20(30)
C(51)	36(7)	24(6)	18(6)	4(5)	13(5)	8(5)
C(52)	36(7)	29(7)	23(6)	12(5)	12(5)	10(6)

C(53)	46(8)	38(8)	32(7)	20(6)	21(6)	15(6)
C(54)	33(8)	37(8)	31(7)	10(6)	11(6)	0(6)
C(55)	30(7)	28(7)	24(6)	5(5)	7(5)	3(5)
C(56)	26(7)	33(7)	30(7)	13(6)	10(5)	7(5)
O(5)	25(5)	41(5)	31(5)	21(4)	10(4)	11(4)
O(6)	30(5)	36(5)	44(6)	18(5)	6(4)	-1(4)
C(57)	27(7)	58(10)	54(9)	34(8)	16(7)	14(7)
C(58)	43(9)	51(9)	51(9)	35(8)	3(7)	-4(7)
C(59)	18(7)	64(10)	58(10)	41(8)	9(6)	11(7)
C(61)	20(6)	47(8)	30(7)	19(6)	6(5)	3(6)
C(62)	34(7)	40(8)	35(7)	26(6)	15(6)	11(6)
C(63)	36(8)	60(10)	37(8)	31(8)	10(6)	6(7)
C(64)	29(8)	63(10)	54(9)	39(9)	16(7)	11(7)
C(65)	28(7)	45(9)	52(9)	22(7)	11(7)	5(6)
C(66)	25(7)	45(9)	49(9)	27(7)	10(6)	5(6)
O(7)	89(9)	48(7)	49(7)	15(6)	46(7)	7(6)
O(8)	44(6)	38(6)	79(8)	27(6)	22(6)	0(5)
C(67)	66(12)	74(12)	34(9)	4(8)	29(8)	-6(10)
C(68)	35(9)	58(10)	71(12)	44(9)	7(8)	4(7)
C(69)	103(18)	72(14)	77(15)	-11(12)	55(14)	-45(13)
C(71)	42(8)	20(6)	25(6)	5(5)	13(6)	1(6)
C(72)	37(8)	37(8)	24(7)	10(6)	5(6)	4(6)
C(73)	35(8)	45(8)	29(7)	11(6)	13(6)	-6(6)
C(74)	47(9)	32(7)	26(7)	10(6)	15(6)	-1(6)
C(75)	49(9)	25(7)	25(7)	8(6)	13(6)	0(6)
C(76)	36(7)	31(7)	20(6)	6(5)	7(5)	0(6)
O(9)	29(5)	70(8)	57(7)	34(6)	15(5)	5(5)
O(10)	49(6)	49(6)	44(6)	30(5)	17(5)	14(5)
C(77)	48(9)	42(9)	45(9)	24(7)	8(7)	15(7)
C(78)	55(10)	61(11)	65(11)	38(9)	31(9)	2(8)
C(79)	34(9)	140(20)	100(16)	83(16)	35(10)	19(11)
C(81)	34(8)	33(7)	41(8)	11(6)	15(6)	5(6)
C(82)	59(10)	35(8)	40(9)	15(7)	10(8)	-8(7)
C(83)	68(12)	33(9)	71(12)	21(8)	21(10)	-4(8)
C(84)	44(9)	37(8)	47(9)	20(7)	13(7)	-1(7)
C(85)	28(7)	53(9)	47(9)	30(8)	10(6)	9(7)

C(86)	37(8)	31(7)	36(8)	16(6)	6(6)	3(6)
O(11)	98(10)	30(6)	48(7)	11(5)	20(7)	-7(6)
O(12)	44(6)	46(6)	51(6)	29(5)	18(5)	13(5)
C(87)	95(15)	34(9)	41(10)	7(8)	2(9)	-12(9)
C(88)	52(10)	42(9)	49(10)	18(8)	14(8)	-7(7)
C(89)	41(8)	45(9)	38(8)	22(7)	14(7)	13(7)
Cl(1S)	99(9)	71(6)	63(6)	21(5)	33(6)	37(6)
Cl(2S)	104(9)	117(9)	63(6)	49(6)	36(6)	67(8)
Cl(4S)	29(8)	19(7)	116(16)	17(8)	-19(9)	-1(6)
Cl(4A)	50(10)	33(8)	113(16)	33(9)	21(10)	14(7)
Cl(6S)	260(20)	97(10)	65(8)	28(7)	50(11)	19(12)

Table S5. Hydrogen coordinates (x 10^4) and isotropic displacement parameters ($Å^2$x 10^3) for raj0ea.

	x	y	z	U(eq)
H(10A)	2391	-5135	-4151	51
H(10B)	3302	-4596	-4134	51
H(13)	3273	-3245	-4138	36
H(15)	1659	-1882	-3289	30
H(17A)	153	-4632	-3974	106
H(17B)	739	-5210	-4365	106
H(18A)	140	-5356	-3127	129
H(18B)	705	-5945	-3538	129
H(19A)	-914	-6653	-4218	412
H(19B)	-539	-6430	-4842	412
H(19C)	-1104	-5860	-4420	412
H(20A)	731	-2356	-2656	38
H(20B)	413	-3349	-2903	38
H(23)	761	-3841	-1888	41
H(25)	2895	-2550	226	34
H(27A)	2091	-552	-685	68
H(27B)	1259	-1011	-1532	68
H(28A)	2261	-237	-2016	88
H(28B)	1667	249	-1556	88
H(29A)	3184	1047	-1024	103
H(29B)	3515	307	-796	103
H(29C)	2920	813	-355	103
H(30A)	3573	-762	-30	33
H(30B)	3935	-1287	492	33
H(33)	4425	-489	-777	33
H(35)	5978	-1999	-1574	39
H(37A)	4932	-1947	939	45
H(37B)	5679	-2390	680	45
H(38A)	4099	-3326	725	64
H(38B)	4979	-3645	648	64

H(39A)	4974	-3342	1934	132
H(39B)	4952	-2396	2049	132
H(39C)	5834	-2713	1972	132
H(40A)	5987	-3262	-1422	46
H(40B)	5754	-3338	-677	46
H(43)	4581	-4499	-960	40
H(45)	2523	-5622	-3141	48
H(47A)	5580	-4178	-3029	143
H(47B)	4714	-4384	-3817	143
H(48A)	5819	-3802	-3938	281
H(48B)	5158	-3216	-3843	281
H(49A)	6361	-2649	-3673	305
H(49B)	6817	-2936	-2963	305
H(49C)	6142	-2337	-2864	305
H(53)	2091	-590	-4962	42
H(56)	4123	-1909	-3778	35
H(57A)	58	-1568	-4967	64
H(57B)	654	-1463	-5496	64
H(57C)	842	-722	-4648	64
H(58A)	4014	-264	-5329	74
H(58B)	4241	455	-4458	74
H(58C)	3267	224	-5150	74
H(59A)	6071	-1100	-4016	65
H(59B)	5248	-1925	-4373	65
H(59C)	5518	-1253	-3472	65
H(63)	1340	-4185	1154	51
H(66)	1160	-4949	-1527	46
H(67A)	1686	-1976	1740	94
H(67B)	1803	-2819	1870	94
H(67C)	829	-2779	1344	94
H(68A)	402	-6346	-43	80
H(68B)	658	-5647	844	80
H(68C)	1436	-6019	576	80
H(69A)	211	-7094	-2306	146
H(69B)	954	-6282	-2154	146
H(69C)	-69	-6234	-2238	146

H(73)	7247	414	-1881	46
H(76)	4257	-456	-2060	38
H(77A)	3204	734	-3050	68
H(77B)	3703	869	-2134	68
H(77C)	3298	-62	-2824	68
H(78A)	5663	933	-3272	84
H(78B)	6708	916	-2941	84
H(78C)	6353	1649	-2434	84
H(79A)	8414	-437	-633	121
H(79B)	8160	407	-673	121
H(79C)	8094	-341	-1475	121
H(83)	2089	-7781	-2316	72
H(86)	3498	-4994	-562	44
H(87A)	2252	-7778	-4107	101
H(87B)	1575	-7812	-3630	101
H(87C)	2484	-8123	-3409	101
H(88A)	1793	-8150	-1276	77
H(88B)	1608	-7377	-645	77
H(88C)	2572	-7598	-414	77
H(89A)	3324	-5045	1225	61
H(89B)	3096	-4687	536	61
H(89C)	4004	-5000	753	61
H(1S1)	-5850	-6320	-3214	63
H(1S2)	-4957	-6175	-3443	63
H(2S1)	-8164	-7551	-6114	53
H(2S2)	-7942	-6568	-5968	53
H(2S3)	-7804	-7183	-5887	46
H(2S4)	-8733	-7479	-6662	46
H(3S1)	-992	-2962	-1269	94
H(3S2)	65	-2638	-1106	94
H(3S3)	-1403	-3327	-2204	67
H(3S4)	-601	-3116	-1357	67

Table S1 Crystal data and structure refinement for rathf.

Identification code	rathf
Empirical formula	$C_{77}H_{90}Cl_2O_{12}$
Formula weight	1278.39
Temperature/K	173(2)
Crystal system	triclinic
Space group	P-1
a/Å	12.8595(7)
b/Å	16.9450(10)
c/Å	17.3999(10)
α/°	94.4670(10)
β/°	92.1880(10)
γ/°	107.8300(10)
Volume/Å^3	3590.7(4)
Z	2
ρ_{calc}g/cm^3	1.182
μ/mm^{-1}	0.150
F(000)	1364.0
Crystal size/mm^3	0.4 × 0.3 × 0.26
Radiation	MoKα (λ = 0.71073)
2Θ range for data collection/°	4.2 to 50
Index ranges	-15 ≤ h ≤ 15, -20 ≤ k ≤ 20, -20 ≤ l ≤ 20
Reflections collected	26830
Independent reflections	12608 [R_{int} = 0.0299, R_{sigma} = 0.0478]
Data/restraints/parameters	12608/0/809
Goodness-of-fit on F^2	1.025
Final R indexes [I>=2σ (I)]	R_1 = 0.0586, wR_2 = 0.1454
Final R indexes [all data]	R_1 = 0.0866, wR_2 = 0.1565
Largest diff. peak/hole / e Å^{-3}	0.70/-0.25

Table S2 Fractional Atomic Coordinates (×10^4) and Equivalent Isotropic Displacement Parameters (Å^2×10^3) for rathf. U_{eq} is defined as 1/3 of of the trace of the orthogonalised U_{IJ} tensor.

Atom	*x*	*y*	*z*	U(eq)
O1	800.0(12)	2160.8(9)	6183.8(8)	37.3(4)
O2	1286.4(17)	803.9(13)	9373.4(10)	68.3(6)
O3	5538.7(16)	2779.0(13)	10159.7(11)	61.0(5)
O4	3241.6(13)	523.4(9)	6405.2(8)	40.2(4)

Table S2 Fractional Atomic Coordinates (×10^4) and Equivalent Isotropic Displacement Parameters (Å^2×10^3) for rathf. U_{eq} is defined as 1/3 of of the trace of the orthogonalised U_{IJ} tensor.

Atom	*x*	*y*	*z*	U(eq)
O5	1936.5(14)	45.8(10)	2963.6(9)	46.3(4)
O6	-429.4(14)	2269.6(11)	2860.2(10)	50.9(4)
O7	5025.3(11)	2748.6(9)	4885.3(8)	34.5(4)
O8	8258.9(12)	3193.6(10)	7702.4(9)	44.6(4)
O9	5642.1(15)	799.5(11)	9380.3(9)	52.7(5)
O10	4208.9(12)	4017.6(9)	7301.2(8)	35.3(4)
O11	2390.7(15)	3292.4(11)	2284.3(9)	52.7(5)
O12	1647.3(13)	5176.7(9)	4816.6(9)	42.2(4)
C1	1887.7(18)	3658.9(14)	7128.4(13)	37.1(5)
C2	2623.3(17)	3893.9(12)	6466.7(12)	31.9(5)
C3	3745.3(17)	4013.5(12)	6566.1(12)	29.7(5)
C4	4406.9(16)	4101.4(12)	5941.1(12)	29.5(5)
C5	3929.2(17)	4133.9(12)	5219.4(12)	29.8(5)
C6	2828.0(17)	4073.4(12)	5105.3(12)	29.7(5)
C7	2183.8(17)	3939.8(12)	5735.8(12)	32.3(5)
C8	4603(2)	4835.9(14)	7698.9(13)	44.0(6)
C9	5094(2)	4786.3(16)	8480.2(15)	54.1(7)
C10	5517(3)	5632.0(19)	8945.4(16)	69.3(9)
C11	2377.5(16)	4137.2(13)	4317.1(13)	31.7(5)
C12	1813.3(17)	4703.6(13)	4181.0(13)	34.3(5)
C13	1498(2)	4797.2(15)	3430.5(14)	45.7(6)
C14	1709(2)	4329.2(17)	2801.8(15)	49.8(6)
C15	2247.3(19)	3751.9(14)	2935.6(13)	40.3(6)
C16	2581.0(17)	3662.4(13)	3680.1(13)	34.9(5)
C17	1173(2)	5813.1(16)	4685.0(17)	54.9(7)
C18	1372(3)	4434(2)	1986.7(17)	88.0(11)
C19	2968(2)	2714.7(16)	2363.6(15)	51.9(7)
C20	5594.6(17)	4128.6(13)	6042.8(13)	32.3(5)
C21	5701.0(16)	3287.4(13)	6195.3(12)	29.3(5)
C22	5328.6(16)	2612.1(13)	5629.1(12)	29.3(5)
C23	5236.5(17)	1802.8(13)	5792.3(12)	30.7(5)
C24	5635.2(17)	1694.3(13)	6514.0(12)	31.0(5)
C25	6104.7(16)	2352.5(13)	7066.9(12)	30.8(5)
C26	6111.5(16)	3146.8(13)	6903.9(12)	31.0(5)
C27	5958(2)	3021.2(16)	4434.2(13)	43.4(6)
C28	5609(2)	3202.9(19)	3655.0(15)	57.0(7)
C29	6592(3)	3566(2)	3185.4(17)	72.4(9)
C30	6546.5(18)	2194.1(13)	7829.3(12)	32.8(5)

Table S2 Fractional Atomic Coordinates (×10⁴) and Equivalent Isotropic Displacement Parameters (Å²×10³) for rathf. U_{eq} is defined as 1/3 of of the trace of the orthogonalised U_{IJ} tensor.

Atom	*x*	*y*	*z*	U(eq)
C31	7613.2(18)	2604.1(15)	8130.0(13)	38.8(5)
C32	7985(2)	2404.7(16)	8829.4(14)	46.4(6)
C33	7319(2)	1792.5(17)	9239.0(14)	47.3(6)
C34	6257(2)	1380.5(15)	8936.1(13)	41.5(6)
C35	5882.8(19)	1575.5(14)	8239.6(12)	36.5(5)
C36	9346.8(19)	3598.9(17)	8005.0(16)	53.4(7)
C37	7723(2)	1590(2)	10003.0(16)	71.1(9)
C38	4569(2)	328.0(16)	9079.6(15)	50.9(7)
C39	4682.7(17)	1066.3(13)	5209.8(13)	34.3(5)
C40	3460.3(18)	923.1(12)	5108.5(12)	32.0(5)
C41	2795.9(18)	714.0(12)	5729.2(12)	32.0(5)
C42	1713.8(18)	707.7(12)	5697.5(12)	32.3(5)
C43	1269.2(18)	839.0(12)	4996.2(12)	32.4(5)
C44	1872.7(17)	984.9(12)	4345.7(12)	31.3(5)
C45	2977.5(17)	1045.2(12)	4420.4(12)	31.2(5)
C46	3091(3)	-347.4(16)	6391.0(16)	59.0(7)
C47	3499(3)	-522(2)	7144.0(19)	80.0(10)
C48	3416(3)	-1390.8(19)	7214(2)	83.6(10)
C49	1331.0(17)	1086.0(13)	3607.0(12)	32.4(5)
C50	1347.4(18)	598.9(13)	2920.0(12)	36.1(5)
C51	755(2)	662.1(15)	2260.7(13)	45.3(6)
C52	150(2)	1216.4(16)	2250.4(13)	45.2(6)
C53	154.7(19)	1713.5(14)	2919.2(13)	38.8(5)
C54	728.9(17)	1642.5(13)	3589.4(13)	34.4(5)
C55	1916(3)	-496.5(18)	2292.8(15)	61.4(8)
C56	-505(3)	1276(2)	1527.0(16)	76.5(10)
C57	-521(2)	2752.3(16)	3537.3(15)	49.6(6)
C58	1049.9(18)	580.3(13)	6405.9(12)	36.3(5)
C59	1431.3(17)	1314.4(13)	7015.4(12)	33.5(5)
C60	1353.1(17)	2091.4(14)	6862.1(12)	32.9(5)
C61	1839.7(17)	2807.1(13)	7365.3(12)	33.0(5)
C62	2337.8(18)	2713.9(14)	8060.1(12)	35.0(5)
C63	2354.7(18)	1939.5(14)	8262.4(12)	35.6(5)
C64	1921.4(18)	1251.7(14)	7727.0(12)	35.8(5)
C65	-344.5(19)	1974.3(16)	6261.8(14)	46.0(6)
C66	-884(2)	2022.0(18)	5492.4(15)	52.1(7)
C67	-2106(2)	1806(2)	5525.3(18)	68.6(8)
C68	2883(2)	1874.9(15)	9024.0(13)	40.1(6)

Table S2 Fractional Atomic Coordinates (×10⁴) and Equivalent Isotropic Displacement Parameters (Å²×10³) for rathf. U_{eq} is defined as 1/3 of of the trace of the orthogonalised U_{IJ} tensor.

Atom	*x*	*y*	*z*	U(eq)
C69	2347(2)	1322.6(17)	9542.0(14)	48.9(6)
C70	2889(3)	1274.6(18)	10237.8(14)	56.8(7)
C71	3950(3)	1751.8(18)	10436.2(14)	54.7(7)
C72	4479(2)	2322.4(17)	9928.1(14)	48.8(6)
C73	3946(2)	2375.6(15)	9237.8(13)	43.0(6)
C74	451(3)	1177(3)	9438(2)	127.7(18)
C75	4556(3)	1664(2)	11173.0(16)	73.6(9)
C76	6103(2)	3378(2)	9677.7(17)	65.4(8)

Table S3 Anisotropic Displacement Parameters (Å²×10³) for rathf. The Anisotropic displacement factor exponent takes the form: $-2\pi^2[h^2a^{*2}U_{11}+2hka^*b^*U_{12}+\ldots]$.

Atom	U_{11}	U_{22}	U_{33}	U_{23}	U_{13}	U_{12}
O1	33.2(9)	44.4(9)	33.9(8)	10.6(7)	1.1(7)	9.8(7)
O2	61.7(13)	91.8(15)	47.8(11)	17.6(11)	11.3(10)	15.1(12)
O3	63.7(13)	74.9(13)	48.6(11)	-6.1(10)	-15.2(10)	32.9(11)
O4	46.3(10)	40.8(9)	34.2(9)	10.0(7)	-0.7(7)	13.7(7)
O5	61.8(11)	45.8(10)	36.7(9)	-0.1(7)	0.4(8)	25.8(9)
O6	62.5(12)	54.4(11)	43.2(10)	6.1(8)	-5.3(8)	29.6(9)
O7	32.7(8)	41.8(9)	31.0(8)	9.7(7)	0.2(6)	13.2(7)
O8	31.1(9)	57.8(10)	43.7(9)	13.3(8)	1.4(7)	10.1(8)
O9	60.3(12)	61.0(11)	39.6(10)	24.0(9)	3.8(8)	18.1(9)
O10	39.6(9)	32.2(8)	33.9(8)	4.9(6)	-0.7(7)	10.8(7)
O11	62.1(12)	61.4(11)	39.0(10)	-0.2(8)	0.2(8)	27.3(9)
O12	46.1(10)	40.6(9)	46.5(10)	3.9(7)	-2.3(7)	23.8(8)
C1	37.2(13)	39.1(13)	37.5(13)	5.7(10)	5.7(10)	14.7(10)
C2	32.8(12)	26.8(11)	38.4(12)	6.6(9)	5.4(10)	11.6(9)
C3	34.4(12)	23.3(11)	33.4(12)	5.8(9)	0.8(9)	11.3(9)
C4	28.4(11)	22.8(10)	36.7(12)	6.5(9)	1.4(9)	6.0(9)
C5	32.0(12)	23.9(11)	35.8(12)	8.8(9)	7.4(9)	10.0(9)
C6	29.2(11)	22.6(10)	37.9(12)	7.6(9)	1.0(9)	8.0(9)
C7	27.6(11)	29.9(11)	42.3(13)	9.0(10)	4.0(10)	11.8(9)
C8	49.1(15)	39.4(13)	42.5(14)	-1.4(11)	1.8(11)	13.5(11)
C9	59.7(17)	55.5(16)	42.0(15)	0.7(12)	-4.1(12)	11.9(13)
C10	81(2)	67.5(19)	46.8(17)	-6.7(14)	0.2(15)	8.3(17)
C11	24.7(11)	28.1(11)	39.5(13)	7.7(9)	0.6(9)	3.1(9)
C12	27.8(12)	33.3(12)	41.8(13)	6.5(10)	-0.9(10)	9.2(10)

Table S3 Anisotropic Displacement Parameters (Å^2×10^3) for rathf. The Anisotropic displacement factor exponent takes the form: $-2\pi^2[h^2a^{*2}U_{11}+2hka^*b^*U_{12}+\ldots]$.

Atom	U_{11}	U_{22}	U_{33}	U_{23}	U_{13}	U_{12}
C13	45.0(15)	47.4(15)	49.0(15)	6.5(12)	-10.2(11)	21.7(12)
C14	52.5(16)	55.7(16)	43.6(15)	7.6(12)	-7.7(12)	21.0(13)
C15	37.6(13)	43.6(14)	37.6(13)	1.5(11)	0.2(10)	10.4(11)
C16	28.0(12)	32.6(12)	43.2(13)	8.9(10)	3.5(10)	6.9(9)
C17	60.3(17)	46.3(15)	67.4(18)	3.5(13)	-2.9(14)	31.5(13)
C18	121(3)	121(3)	47.3(18)	4.0(18)	-17.2(18)	78(3)
C19	58.6(17)	49.4(15)	49.1(16)	2.5(12)	12.8(13)	18.1(13)
C20	29.1(12)	30.8(11)	36.2(12)	8.3(9)	1.9(9)	7.0(9)
C21	22.0(11)	32.1(11)	34.0(12)	8.6(9)	3.1(9)	7.1(9)
C22	24.8(11)	38.0(12)	28.1(11)	7.4(9)	0.4(9)	13.3(9)
C23	30.8(12)	33.8(12)	31.1(12)	4.6(9)	3.9(9)	14.6(10)
C24	31.4(12)	30.0(11)	34.2(12)	6.9(9)	3.5(9)	12.4(9)
C25	27.0(11)	38.7(12)	30.1(11)	8.4(10)	4.2(9)	13.6(10)
C26	27.9(11)	33.7(12)	31.5(12)	2.6(9)	1.2(9)	9.7(9)
C27	45.1(14)	55.0(15)	36.7(13)	13.1(11)	9.2(11)	22.2(12)
C28	69.9(19)	76.0(19)	44.4(15)	23.5(14)	17.1(13)	45.1(16)
C29	101(3)	79(2)	58.6(19)	34.1(16)	39.6(17)	48.3(19)
C30	37.0(13)	39.5(12)	28.0(11)	5.1(10)	2.1(9)	20.5(10)
C31	38.4(13)	48.0(14)	35.1(13)	7.3(11)	4.8(10)	19.9(11)
C32	38.3(14)	66.8(17)	38.0(14)	6.3(12)	-3.6(11)	22.5(13)
C33	49.8(16)	67.8(17)	33.8(13)	12.2(12)	1.5(11)	30.5(14)
C34	49.8(15)	48.1(14)	33.5(13)	12.3(11)	5.6(11)	23.1(12)
C35	40.7(13)	39.9(13)	33.8(12)	6.0(10)	1.5(10)	19.3(11)
C36	35.0(14)	67.0(18)	57.2(17)	9.4(14)	1.7(12)	13.7(13)
C37	65(2)	106(3)	48.8(17)	29.5(17)	-6.5(14)	32.7(18)
C38	62.6(18)	50.7(15)	41.7(14)	16.9(12)	6.4(12)	17.4(13)
C39	37.9(13)	34.3(12)	35.0(12)	4.8(10)	2.9(10)	17.3(10)
C40	37.9(12)	25.2(11)	32.8(12)	1.4(9)	2.4(10)	9.7(9)
C41	38.2(13)	27.8(11)	27.7(12)	2.0(9)	-3.3(9)	7.8(10)
C42	37.0(13)	26.3(11)	32.0(12)	5.3(9)	4.2(9)	6.4(9)
C43	32.1(12)	29.3(11)	34.4(12)	2.5(9)	0.5(9)	7.5(9)
C44	35.8(12)	25.3(11)	31.1(12)	2.8(9)	2.5(9)	7.1(9)
C45	36.4(13)	29.3(11)	27.1(11)	2.6(9)	4.0(9)	8.8(10)
C46	78(2)	48.5(16)	56.6(17)	14.3(13)	-2.3(14)	27.1(15)
C47	104(3)	73(2)	72(2)	30.7(18)	-6.5(19)	34.6(19)
C48	104(3)	60(2)	87(2)	13.4(17)	-22(2)	26.9(19)
C49	32.9(12)	29.0(11)	32.6(12)	8.3(9)	3.4(9)	4.2(9)
C50	41.7(13)	34.4(12)	33.5(13)	9.2(10)	5.3(10)	11.9(10)
C51	62.1(17)	46.1(14)	28.8(13)	5.3(11)	3.3(11)	17.9(13)

Table S3 Anisotropic Displacement Parameters (Å^2×10^3) for rathf. The Anisotropic displacement factor exponent takes the form: $-2\pi^2[h^2a^{*2}U_{11}+2hka^*b^*U_{12}+\ldots]$.

Atom	U_{11}	U_{22}	U_{33}	U_{23}	U_{13}	U_{12}
C52	55.1(16)	50.5(15)	33.6(13)	11.5(11)	-0.2(11)	20.4(13)
C53	40.4(13)	39.2(13)	39.8(14)	11.2(11)	3.5(10)	15.1(11)
C54	32.6(12)	33.6(12)	34.2(12)	5.7(10)	2.9(9)	5.2(10)
C55	93(2)	60.5(17)	43.4(16)	0.3(13)	7.9(14)	43.2(17)
C56	114(3)	92(2)	39.8(16)	3.1(15)	-15.8(16)	60(2)
C57	43.8(15)	48.9(15)	59.2(17)	3.2(13)	-1.7(12)	20.0(12)
C58	37.7(13)	34.1(12)	34.5(12)	9.2(10)	4.4(10)	5.5(10)
C59	30.9(12)	37.9(12)	30.3(12)	6.2(10)	7.4(9)	7.1(10)
C60	28.9(12)	40.6(13)	30.3(12)	11.5(10)	8.0(9)	9.7(10)
C61	30.9(12)	38.6(13)	32.0(12)	9.4(10)	11.3(9)	12.1(10)
C62	34.7(12)	38.8(13)	31.6(12)	1.6(10)	6.4(10)	11.4(10)
C63	37.5(13)	42.2(13)	30.0(12)	5.4(10)	6.4(10)	15.7(10)
C64	38.5(13)	38.9(13)	32.8(12)	9.3(10)	9.0(10)	14.1(10)
C65	36.5(14)	56.4(16)	46.1(15)	12.2(12)	3.1(11)	13.9(12)
C66	44.7(15)	67.0(18)	50.7(16)	9.6(13)	1.5(12)	25.6(13)
C67	45.1(17)	87(2)	74(2)	6.9(17)	-2.6(14)	21.6(16)
C68	50.5(15)	46.2(14)	30.0(12)	2.7(10)	4.1(11)	24.2(12)
C69	56.8(17)	59.3(16)	33.8(14)	8.6(12)	7.3(12)	21.1(14)
C70	81(2)	66.4(18)	34.2(14)	11.4(13)	11.2(14)	37.7(17)
C71	75(2)	71.2(18)	31.7(14)	-1.6(13)	-4.0(13)	45.9(17)
C72	56.2(17)	61.5(17)	37.0(14)	-6.5(12)	-5.2(12)	34.1(14)
C73	54.2(16)	45.9(14)	35.0(13)	0.0(11)	1.6(11)	25.4(12)
C74	71(3)	212(5)	86(3)	-29(3)	16(2)	31(3)
C75	98(2)	101(2)	40.6(16)	4.5(16)	-5.8(15)	60(2)
C76	53.3(18)	81(2)	60.5(19)	-13.3(17)	-9.7(14)	25.3(16)

Table S4 Bond Lengths for rathf.

Atom	Atom	Length/Å	Atom	Atom	Length/Å
O1	C60	1.386(2)	C23	C39	1.515(3)
O1	C65	1.422(3)	C24	C25	1.382(3)
O2	C69	1.384(3)	C25	C26	1.394(3)
O2	C74	1.408(5)	C25	C30	1.499(3)
O3	C72	1.371(3)	C27	C28	1.496(3)
O3	C76	1.410(4)	C28	C29	1.524(4)
O4	C41	1.392(2)	C30	C31	1.394(3)
O4	C46	1.426(3)	C30	C35	1.397(3)
O5	C50	1.378(3)	C31	C32	1.393(3)

Table S4 Bond Lengths for rathf.

Atom	Atom	Length/Å	Atom	Atom	Length/Å
O5	C55	1.422(3)	C32	C33	1.391(4)
O6	C53	1.380(3)	C33	C34	1.391(4)
O6	C57	1.411(3)	C33	C37	1.511(3)
O7	C22	1.394(2)	C34	C35	1.387(3)
O7	C27	1.434(3)	C39	C40	1.516(3)
O8	C31	1.377(3)	C40	C41	1.402(3)
O8	C36	1.416(3)	C40	C45	1.387(3)
O9	C34	1.371(3)	C41	C42	1.387(3)
O9	C38	1.421(3)	C42	C43	1.390(3)
O10	C3	1.389(2)	C42	C58	1.518(3)
O10	C8	1.433(3)	C43	C44	1.392(3)
O11	C15	1.375(3)	C44	C45	1.393(3)
O11	C19	1.410(3)	C44	C49	1.485(3)
O12	C12	1.377(3)	C46	C47	1.478(4)
O12	C17	1.421(3)	C47	C48	1.457(4)
C1	C2	1.518(3)	C49	C50	1.403(3)
C1	C61	1.515(3)	C49	C54	1.393(3)
C2	C3	1.396(3)	C50	C51	1.382(3)
C2	C7	1.389(3)	C51	C52	1.391(3)
C3	C4	1.395(3)	C52	C53	1.381(3)
C4	C5	1.389(3)	C52	C56	1.515(3)
C4	C20	1.517(3)	C53	C54	1.389(3)
C5	C6	1.393(3)	C58	C59	1.515(3)
C6	C7	1.391(3)	C59	C60	1.396(3)
C6	C11	1.493(3)	C59	C64	1.394(3)
C8	C9	1.496(3)	C60	C61	1.399(3)
C9	C10	1.521(4)	C61	C62	1.389(3)
C11	C12	1.397(3)	C62	C63	1.391(3)
C11	C16	1.399(3)	C63	C64	1.389(3)
C12	C13	1.388(3)	C63	C68	1.493(3)
C13	C14	1.385(4)	C65	C66	1.504(3)
C14	C15	1.389(3)	C66	C67	1.505(4)
C14	C18	1.507(4)	C68	C69	1.395(3)
C15	C16	1.383(3)	C68	C73	1.389(3)
C20	C21	1.516(3)	C69	C70	1.394(4)
C21	C22	1.401(3)	C70	C71	1.372(4)
C21	C26	1.389(3)	C71	C72	1.399(4)
C22	C23	1.393(3)	C71	C75	1.515(4)
C23	C24	1.385(3)	C72	C73	1.383(3)

Table S5 Bond Angles for rathf.

Atom	Atom	Atom	Angle/°	Atom	Atom	Atom	Angle/°
C60	O1	C65	112.08(16)	C32	C33	C37	121.3(2)
C69	O2	C74	116.4(3)	C34	C33	C37	120.5(2)
C72	O3	C76	117.5(2)	O9	C34	C33	115.0(2)
C41	O4	C46	111.27(17)	O9	C34	C35	124.7(2)
C50	O5	C55	117.72(18)	C35	C34	C33	120.4(2)
C53	O6	C57	118.00(18)	C34	C35	C30	121.5(2)
C22	O7	C27	111.51(15)	C23	C39	C40	111.59(17)
C31	O8	C36	116.68(18)	C41	C40	C39	120.50(19)
C34	O9	C38	117.38(18)	C45	C40	C39	121.36(19)
C3	O10	C8	112.57(15)	C45	C40	C41	118.0(2)
C15	O11	C19	118.61(19)	O4	C41	C40	119.02(19)
C12	O12	C17	117.63(18)	C42	C41	O4	118.84(18)
C61	C1	C2	111.48(17)	C42	C41	C40	122.12(19)
C3	C2	C1	120.70(19)	C41	C42	C43	117.40(19)
C7	C2	C1	120.64(19)	C41	C42	C58	121.22(19)
C7	C2	C3	118.52(19)	C43	C42	C58	121.4(2)
O10	C3	C2	119.27(18)	C42	C43	C44	122.4(2)
O10	C3	C4	119.10(18)	C43	C44	C45	118.09(19)
C2	C3	C4	121.59(19)	C43	C44	C49	119.45(19)
C3	C4	C20	121.05(18)	C45	C44	C49	122.45(19)
C5	C4	C3	117.65(19)	C40	C45	C44	121.58(19)
C5	C4	C20	121.27(19)	O4	C46	C47	108.9(2)
C4	C5	C6	122.36(19)	C48	C47	C46	115.3(3)
C5	C6	C11	119.43(18)	C50	C49	C44	122.05(19)
C7	C6	C5	118.10(19)	C54	C49	C44	120.12(19)
C7	C6	C11	122.46(18)	C54	C49	C50	117.72(20)
C2	C7	C6	121.50(19)	O5	C50	C49	116.01(19)
O10	C8	C9	109.18(19)	O5	C50	C51	123.7(2)
C8	C9	C10	112.4(2)	C51	C50	C49	120.2(2)
C12	C11	C6	122.0(2)	C50	C51	C52	121.6(2)
C12	C11	C16	117.9(2)	C51	C52	C56	121.3(2)
C16	C11	C6	119.94(19)	C53	C52	C51	118.5(2)
O12	C12	C11	116.69(19)	C53	C52	C56	120.3(2)
O12	C12	C13	123.20(19)	O6	C53	C52	115.4(2)
C13	C12	C11	120.0(2)	O6	C53	C54	124.2(2)
C14	C13	C12	121.7(2)	C52	C53	C54	120.4(2)
C13	C14	C15	118.4(2)	C53	C54	C49	121.6(2)
C13	C14	C18	121.7(2)	C59	C58	C42	112.71(17)

Table S5 Bond Angles for rathf.

Atom	Atom	Atom	Angle/°	Atom	Atom	Atom	Angle/°
C15	C14	C18	119.8(2)	C60	C59	C58	120.48(19)
O11	C15	C14	114.8(2)	C64	C59	C58	121.6(2)
O11	C15	C16	124.8(2)	C64	C59	C60	117.8(2)
C16	C15	C14	120.4(2)	O1	C60	C59	119.17(19)
C15	C16	C11	121.5(2)	O1	C60	C61	118.97(19)
C21	C20	C4	111.89(16)	C59	C60	C61	121.8(2)
C22	C21	C20	120.18(18)	C60	C61	C1	120.52(19)
C26	C21	C20	121.81(19)	C62	C61	C1	121.5(2)
C26	C21	C22	117.92(18)	C62	C61	C60	117.8(2)
O7	C22	C21	119.40(17)	C61	C62	C63	122.0(2)
C23	C22	O7	118.92(18)	C62	C63	C68	119.6(2)
C23	C22	C21	121.67(18)	C64	C63	C62	118.4(2)
C22	C23	C39	120.98(18)	C64	C63	C68	121.9(2)
C24	C23	C22	117.66(19)	C63	C64	C59	121.8(2)
C24	C23	C39	121.33(18)	O1	C65	C66	108.50(19)
C25	C24	C23	122.49(19)	C65	C66	C67	111.5(2)
C24	C25	C26	118.24(19)	C69	C68	C63	122.4(2)
C24	C25	C30	119.78(18)	C73	C68	C63	119.9(2)
C26	C25	C30	121.93(19)	C73	C68	C69	117.6(2)
C21	C26	C25	121.5(2)	O2	C69	C68	121.2(2)
O7	C27	C28	110.01(19)	O2	C69	C70	118.8(2)
C27	C28	C29	111.4(2)	C70	C69	C68	120.0(3)
C31	C30	C25	122.98(19)	C71	C70	C69	122.2(3)
C31	C30	C35	118.2(2)	C70	C71	C72	118.0(2)
C35	C30	C25	118.74(19)	C70	C71	C75	122.0(3)
O8	C31	C30	116.93(19)	C72	C71	C75	120.0(3)
O8	C31	C32	123.0(2)	O3	C72	C71	115.1(2)
C32	C31	C30	120.0(2)	O3	C72	C73	124.7(3)
C33	C32	C31	121.6(2)	C73	C72	C71	120.1(3)
C32	C33	C34	118.3(2)	C72	C73	C68	122.0(2)

Table S6 Torsion Angles for rathf.

A	B	C	D	Angle/°	A	B	C	D	Angle/°
O1	C60	C61	C1	-7.7(3)	C31	C32	C33	C37	-179.1(2)
O1	C60	C61	C62	176.59(18)	C32	C33	C34	O9	-178.5(2)
O1	C65	C66	C67	178.3(2)	C32	C33	C34	C35	0.6(4)
O2	C69	C70	C71	178.2(2)	C33	C34	C35	C30	-1.0(3)
O3	C72	C73	C68	177.8(2)	C35	C30	C31	O8	178.17(19)

Table S6 Torsion Angles for rathf.

A	B	C	D	Angle/°	A	B	C	D	Angle/°
O4	C41	C42	C43	-175.27(18)	C35	C30	C31	C32	-1.2(3)
O4	C41	C42	C58	6.0(3)	C36	O8	C31	C30	-179.1(2)
O4	C46	C47	C48	-179.0(3)	C36	O8	C31	C32	0.3(3)
O5	C50	C51	C52	178.4(2)	C37	C33	C34	O9	0.0(4)
O6	C53	C54	C49	-178.0(2)	C37	C33	C34	C35	179.1(2)
O7	C22	C23	C24	174.29(17)	C38	O9	C34	C33	-176.7(2)
O7	C22	C23	C39	-7.8(3)	C38	O9	C34	C35	4.2(3)
O7	C27	C28	C29	175.1(2)	C39	C23	C24	C25	-177.39(19)
O8	C31	C32	C33	-178.5(2)	C39	C40	C41	O4	-9.1(3)
O9	C34	C35	C30	178.0(2)	C39	C40	C41	C42	169.55(19)
O10	C3	C4	C5	-177.05(17)	C39	C40	C45	C44	-174.35(18)
O10	C3	C4	C20	5.1(3)	C40	C41	C42	C43	6.1(3)
O10	C8	C9	C10	-179.1(2)	C40	C41	C42	C58	-172.66(19)
O11	C15	C16	C11	-178.1(2)	C41	O4	C46	C47	-175.6(2)
O12	C12	C13	C14	177.9(2)	C41	C40	C45	C44	1.6(3)
C1	C2	C3	O10	-7.9(3)	C41	C42	C43	C44	-1.0(3)
C1	C2	C3	C4	169.88(19)	C41	C42	C58	C59	69.5(3)
C1	C2	C7	C6	-173.59(19)	C42	C43	C44	C45	-3.6(3)
C1	C61	C62	C63	-176.1(2)	C42	C43	C44	C49	177.54(19)
C2	C1	C61	C60	-66.5(3)	C42	C58	C59	C60	64.0(3)
C2	C1	C61	C62	109.0(2)	C42	C58	C59	C64	-112.6(2)
C2	C3	C4	C5	5.1(3)	C43	C42	C58	C59	-109.2(2)
C2	C3	C4	C20	-172.70(18)	C43	C44	C45	C40	3.2(3)
C3	O10	C8	C9	-179.66(19)	C43	C44	C49	C50	-125.5(2)
C3	C2	C7	C6	2.2(3)	C43	C44	C49	C54	50.7(3)
C3	C4	C5	C6	-0.7(3)	C44	C49	C50	O5	-2.7(3)
C3	C4	C20	C21	70.8(2)	C44	C49	C50	C51	174.4(2)
C4	C5	C6	C7	-2.8(3)	C44	C49	C54	C53	-175.8(2)
C4	C5	C6	C11	178.30(19)	C45	C40	C41	O4	174.91(18)
C4	C20	C21	C22	64.4(2)	C45	C40	C41	C42	-6.5(3)
C4	C20	C21	C26	-112.2(2)	C45	C44	C49	C50	55.7(3)
C5	C4	C20	C21	-106.9(2)	C45	C44	C49	C54	-128.2(2)
C5	C6	C7	C2	2.1(3)	C46	O4	C41	C40	-91.2(2)
C5	C6	C11	C12	-126.6(2)	C46	O4	C41	C42	90.1(2)
C5	C6	C11	C16	48.9(3)	C49	C44	C45	C40	-

Table S6 Torsion Angles for rathf.

A	B	C	D	Angle/˚	A	B	C	D	Angle/˚
									177.95(19)
C6	C11	C12	O12	-2.9(3)	C49	C50	C51	C52	1.5(4)
C6	C11	C12	C13	173.9(2)	C50	C49	C54	C53	0.5(3)
C6	C11	C16	C15	-175.1(2)	C50	C51	C52	C53	0.3(4)
C7	C2	C3	O10	176.30(18)	C50	C51	C52	C56	-179.5(3)
C7	C2	C3	C4	-5.9(3)	C51	C52	C53	O6	177.7(2)
C7	C6	C11	C12	54.6(3)	C51	C52	C53	C54	-1.6(4)
C7	C6	C11	C16	-129.9(2)	C52	C53	C54	C49	1.3(3)
C8	O10	C3	C2	-91.5(2)	C54	C49	C50	O5	-178.94(18)
C8	O10	C3	C4	90.6(2)	C54	C49	C50	C51	-1.8(3)
C11	C6	C7	C2	-179.10(19)	C55	O5	C50	C49	176.1(2)
C11	C12	C13	C14	1.3(4)	C55	O5	C50	C51	-1.0(3)
C12	C11	C16	C15	0.6(3)	C56	C52	C53	O6	-2.5(4)
C12	C13	C14	C15	0.2(4)	C56	C52	C53	C54	178.1(2)
C12	C13	C14	C18	-179.6(3)	C57	O6	C53	C52	175.5(2)
C13	C14	C15	O11	177.8(2)	C57	O6	C53	C54	-5.2(3)
C13	C14	C15	C16	-1.2(4)	C58	C42	C43	C44	177.80(19)
C14	C15	C16	C11	0.8(3)	C58	C59	C60	O1	8.0(3)
C16	C11	C12	O12	-178.50(18)	C58	C59	C60	C61	-170.51(19)
C16	C11	C12	C13	-1.7(3)	C58	C59	C64	C63	174.5(2)
C17	O12	C12	C11	173.9(2)	C59	C60	C61	C1	170.76(19)
C17	O12	C12	C13	-2.8(3)	C59	C60	C61	C62	-4.9(3)
C18	C14	C15	O11	-2.4(4)	C60	O1	C65	C66	-177.2(2)
C18	C14	C15	C16	178.6(3)	C60	C59	C64	C63	-2.1(3)
C19	O11	C15	C14	177.5(2)	C60	C61	C62	C63	-0.4(3)
C19	O11	C15	C16	-3.5(3)	C61	C1	C2	C3	-64.9(3)
C20	C4	C5	C6	177.14(18)	C61	C1	C2	C7	110.7(2)
C20	C21	C22	O7	10.3(3)	C61	C62	C63	C64	4.3(3)
C20	C21	C22	C23	-168.66(18)	C61	C62	C63	C68	-178.8(2)
C20	C21	C26	C25	173.41(19)	C62	C63	C64	C59	-3.0(3)
C21	C22	C23	C24	-6.7(3)	C62	C63	C68	C69	127.8(2)
C21	C22	C23	C39	171.16(18)	C62	C63	C68	C73	-52.4(3)
C22	O7	C27	C28	-177.1(2)	C63	C68	C69	O2	-0.2(4)
C22	C21	C26	C25	-3.3(3)	C63	C68	C69	C70	178.5(2)
C22	C23	C24	C25	0.5(3)	C63	C68	C73	C72	-178.3(2)
C22	C23	C39	C40	-69.2(2)	C64	C59	C60	O1	-175.36(18)

Table S6 Torsion Angles for rathf.

A	B	C	D	Angle/°	A	B	C	D	Angle/°
C23	C24	C25	C26	4.1(3)	C64	C59	C60	C61	6.2(3)
C23	C24	C25	C30	-178.42(19)	C64	C63	C68	C69	-55.4(3)
C23	C39	C40	C41	-63.1(2)	C64	C63	C68	C73	124.5(2)
C23	C39	C40	C45	112.8(2)	C65	O1	C60	C59	85.7(2)
C24	C23	C39	C40	108.6(2)	C65	O1	C60	C61	-95.7(2)
C24	C25	C26	C21	-2.6(3)	C68	C63	C64	C59	-179.8(2)
C24	C25	C30	C31	126.2(2)	C68	C69	C70	C71	-0.6(4)
C24	C25	C30	C35	-50.1(3)	C69	C68	C73	C72	1.6(3)
C25	C30	C31	O8	1.8(3)	C69	C70	C71	C72	2.3(4)
C25	C30	C31	C32	-177.6(2)	C69	C70	C71	C75	-176.6(2)
C25	C30	C35	C34	177.9(2)	C70	C71	C72	O3	-179.9(2)
C26	C21	C22	O7	-172.93(17)	C70	C71	C72	C73	-2.0(4)
C26	C21	C22	C23	8.1(3)	C71	C72	C73	C68	0.1(4)
C26	C25	C30	C31	-56.4(3)	C73	C68	C69	O2	179.9(2)
C26	C25	C30	C35	127.3(2)	C73	C68	C69	C70	-1.4(4)
C27	O7	C22	C21	82.7(2)	C74	O2	C69	C68	-75.5(3)
C27	O7	C22	C23	-98.3(2)	C74	O2	C69	C70	105.7(3)
C30	C25	C26	C21	179.94(19)	C75	C71	C72	O3	-1.0(3)
C30	C31	C32	C33	0.9(4)	C75	C71	C72	C73	176.9(2)
C31	C30	C35	C34	1.3(3)	C76	O3	C72	C71	-178.5(2)
C31	C32	C33	C34	-0.5(4)	C76	O3	C72	C73	3.7(3)

Table S7 Hydrogen Atom Coordinates (Å×10^4) and Isotropic Displacement Parameters (Å^2×10^3) for rathf.

Atom	*x*	*y*	*z*	U(eq)
H1A	2167	4080	7576	45
H1B	1140	3659	6970	45
H5	4369	4200	4788	36
H7	1426	3879	5665	39
H8A	3992	5073	7753	53
H8B	5162	5204	7400	53
H9A	5705	4551	8419	65
H9B	4535	4404	8768	65
H10A	6128	5993	8692	104
H10B	5767	5562	9467	104
H10C	4927	5885	8976	104
H13	1128	5193	3346	55

Table S7 Hydrogen Atom Coordinates (Å×10^4) and Isotropic Displacement Parameters (Å^2×10^3) for rathf.

Atom	*x*	*y*	*z*	U(eq)
H16	2957	3270	3761	42
H17A	1627	6199	4349	82
H17B	1129	6117	5179	82
H17C	436	5563	4436	82
H18A	2025	4704	1717	132
H18B	886	4781	1997	132
H18C	988	3887	1715	132
H19A	3727	3011	2550	78
H19B	2958	2406	1862	78
H19C	2621	2325	2735	78
H20A	5968	4311	5571	39
H20B	5962	4542	6480	39
H24	5584	1147	6634	37
H26	6404	3602	7286	37
H27A	6319	2583	4375	52
H27B	6492	3529	4702	52
H28A	5189	3603	3718	68
H28B	5123	2683	3373	68
H29A	7037	4107	3439	109
H29B	6336	3637	2665	109
H29C	7033	3186	3150	109
H32	8710	2694	9031	56
H35	5159	1282	8037	44
H36A	9337	3890	8512	80
H36B	9726	4002	7654	80
H36C	9730	3186	8058	80
H37A	7257	1689	10408	107
H37B	8479	1944	10132	107
H37C	7697	1004	9965	107
H38A	4147	706	8976	76
H38B	4208	-48	9457	76
H38C	4614	2	8599	76
H39A	4813	560	5384	41
H39B	5008	1166	4706	41
H43	526	829	4959	39
H45	3410	1173	3989	37
H46A	3496	-521	5972	71
H46B	2304	-664	6292	71
H47A	4277	-182	7240	96

Table S7 Hydrogen Atom Coordinates (Å×10^4) and Isotropic Displacement Parameters (Å^2×10^3) for rathf.

Atom	*x*	*y*	*z*	U(eq)
H47B	3086	-340	7554	96
H48A	2643	-1728	7177	125
H48B	3757	-1439	7714	125
H48C	3792	-1589	6797	125
H51	760	319	1803	54
H54	710	1982	4047	41
H55A	1157	-823	2134	92
H55B	2335	-873	2408	92
H55C	2241	-167	1874	92
H56A	-167	1813	1323	115
H56B	-1256	1233	1652	115
H56C	-516	823	1138	115
H57A	-939	2387	3903	74
H57B	-900	3151	3406	74
H57C	211	3055	3773	74
H58A	271	487	6247	44
H58B	1103	75	6632	44
H62	2677	3193	8408	42
H64	1960	724	7849	43
H65A	-484	2378	6655	55
H65B	-647	1409	6431	55
H66A	-714	1633	5100	62
H66B	-586	2592	5334	62
H67A	-2405	1238	5672	103
H67B	-2433	1845	5017	103
H67C	-2277	2196	5908	103
H70	2510	899	10586	68
H73	4319	2766	8899	52
H74A	648	1613	9871	192
H74B	-237	755	9529	192
H74C	360	1424	8959	192
H75A	4062	1255	11469	110
H75B	4814	2204	11483	110
H75C	5184	1478	11044	110
H76A	6104	3108	9159	98
H76B	6859	3637	9891	98
H76C	5738	3806	9649	98

Experimental

Single crystals of $C_{77}H_{90}Cl_2O_{12}$ **[rathf]** were **[]**. A suitable crystal was selected and **[]** on a **CCD area detector** diffractometer. The crystal was kept at 173(2) K during data collection. Using Olex2 [1], the structure was solved with the Unknown [2] structure solution program using Unknown and refined with the Unknown [3] refinement package using Unknown minimisation.

1. Dolomanov, O.V., Bourhis, L.J., Gildea, R.J, Howard, J.A.K. & Puschmann, H. (2009), J. Appl. Cryst. 42, 339-341.
2.
3.

Crystal structure determination of [rathf]

Crystal Data for $C_{77}H_{90}Cl_2O_{12}$ (M =1278.39 g/mol): triclinic, space group P-1 (no. 2), a = 12.8595(7) Å, b = 16.9450(10) Å, c = 17.3999(10) Å, α = 94.4670(10)°, β = 92.1880(10)°, γ = 107.8300(10)°, V = 3590.7(4) $Å^3$, Z = 2, T = 173(2) K, μ(MoKα) = 0.150 mm^{-1}, $Dcalc$ = 1.182 g/cm^3, 26830 reflections measured (4.2° ≤ 2Θ ≤ 50°), 12608 unique (R_{int} = 0.0299, R_{sigma} = 0.0478) which were used in all calculations. The final R_1 was 0.0586 (>2sigma(I)) and wR_2 was 0.1565 (all data).

Reference

(1) Rathore, R.; Lindeman, S. V.; Rao, K. S. S. P.; Sun, D.; Kochi, J. K. Guest Penetration Deep within the Cavity of Calix[4]arene Hosts: The Tight Binding of Nitric Oxide to Distal (Cofacial) Aromatic Groups. *Angewandte Chemie International Edition* **2000**, *39* (12), 2123-2127. DOI: https://doi.org/10.1002/1521-3773(20000616)39:12<2123::AID-ANIE2123>3.0.CO;2-4.

(2) Rathore, R.; Abdelwahed, S. H.; Guzei, I. A. Synthesis of a Calix[4]arene Derivative for Isolation of a Stable Cation Radical Salt for Use as a Colorimetric Sensor of Nitric Oxide. *Journal of the American Chemical Society* **2004**, *126* (42), 13582-13583. DOI: 10.1021/ja0454900.

(3) Culotta, E.; Koshland, D. E. NO News Is Good News. *Science* **1992**, *258* (5090), 1862-1865. DOI: doi:10.1126/science.1361684.

(4) Tipson, R. S. ON ESTERS OF p-TOLUENESULFONIC ACID. *The Journal of Organic Chemistry* **1944**, *09* (3), 235-241. DOI: 10.1021/jo01185a005.

(5) McHale, D.; Mamalis, P.; Green, J.; Marcinkiewicz, S. 319. Tocopherols. Part I. Synthesis of 7-methyltocol (η-tocopherol). *Journal of the Chemical Society (Resumed)* **1958**, (0), 1600-1603, 10.1039/JR9580001600. DOI: 10.1039/JR9580001600.
